# Identification of hub genes related to the progression of type 1 diabetes by computational analysis

**DOI:** 10.1186/s12902-021-00709-6

**Published:** 2021-04-07

**Authors:** G. Prashanth, Basavaraj Vastrad, Anandkumar Tengli, Chanabasayya Vastrad, Iranna Kotturshetti

**Affiliations:** 1 Department of General Medicine, Basaveshwara Medical College, Chitradurga, Karnataka 577501 India; 2 Department of Biochemistry, Basaveshwar College of Pharmacy, Gadag, Karnataka 582103 India; 3 Department of Pharmaceutical Chemistry, JSS College of Pharmacy, Mysuru and JSS Academy of Higher Education & Research, Mysuru, Karnataka 570015 India; 4 Biostatistics and Bioinformatics, Chanabasava Nilaya, Bharthinagar, Dharwad, Karanataka 580001 India; 5 Department of Ayurveda, Rajiv Gandhi Education Society’s Ayurvedic Medical College, Ron, Karanataka 582209 India

**Keywords:** bioinformatics, type 1 diabetes, differentially expressed genes, enrichment analysis, pathways

## Abstract

**Background:**

Type 1 diabetes (T1D) is a serious threat to childhood life and has fairly complicated pathogenesis. Profound attempts have been made to enlighten the pathogenesis, but the molecular mechanisms of T1D are still not well known.

**Methods:**

To identify the candidate genes in the progression of T1D, expression profiling by high throughput sequencing dataset GSE123658 was downloaded from Gene Expression Omnibus (GEO) database. The differentially expressed genes (DEGs) were identified, and gene ontology (GO) and pathway enrichment analyses were performed. The protein-protein interaction network (PPI), modules, target gene - miRNA regulatory network and target gene - TF regulatory network analysis were constructed and analyzed using HIPPIE, miRNet, NetworkAnalyst and Cytoscape. Finally, validation of hub genes was conducted by using ROC (Receiver operating characteristic) curve and RT-PCR analysis. A molecular docking study was performed.

**Results:**

A total of 284 DEGs were identified, consisting of 142 up regulated genes and 142 down regulated genes. The gene ontology (GO) and pathways of the DEGs include cell-cell signaling, vesicle fusion, plasma membrane, signaling receptor activity, lipid binding, signaling by GPCR and innate immune system. Four hub genes were identified and biological process analysis revealed that these genes were mainly enriched in cell-cell signaling, cytokine signaling in immune system, signaling by GPCR and innate immune system. ROC curve and RT-PCR analysis showed that EGFR, GRIN2B, GJA1, CAP2, MIF, POLR2A, PRKACA, GABARAP, TLN1 and PXN might be involved in the advancement of T1D. Molecular docking studies showed high docking score.

**Conclusions:**

DEGs and hub genes identified in the present investigation help us understand the molecular mechanisms underlying the advancement of T1D, and provide candidate targets for diagnosis and treatment of T1D.

## Introduction

Type 1 diabetes (T1D) (insulin-dependent) is a core challenge for endocrine research around the world [1]. Approximately 5 to 10% of the childhood population is affected with T1D worldwide [2]. T1D affects the eyes, kidneys, heart, peripheral and autonomic nervous systems [3]. Pancreatic cells, particularly β-cells, play a key role in the occurrence and progression of T1D [4]. Treatment for T1D includes targeting β-cells and β-cells regeneration [5]. However, T1D is a complex disease and its biology remains poorly understood [6].

There are several important risk factors for T1D, such as genetic and environmental factors [7, 8]. Previous studies identified aspects of the molecular mechanism of T1D advancement. T1D has been genetically associated with genes and signaling pathways, CTLA-4 [9], SUMO4 [10], CYP27B1 [11], PD-1 [12], KIAA0350 [13], tumor necrosis factor alpha signaling pathways [14], NLRP3 and NLRP1 inflammasomes signaling pathways [15], HIF-1/VEGF signaling pathway [16], l-arginine/NO pathway [17], and CaMKII/NF-κB/TGF-β1 and PPAR-γ signaling pathway [18]. Next-generation sequencing (NGS) has drastically increased the understanding mechanism of T1D, and analyses of these data can provide insight into effective diagnostic and therapeutic T1D treatments [19]. Thus, identifying key molecular biomarkers is essential for early diagnosis, prevention, and treatment of T1D.

It worth a lot of money and time to identify disease related molecular biomarkers by experiment alone. With the wide application of expression profiling by high throughput sequencing data, there were huge genomics data deposited in public databases [20]. The progression of computational tools gives us an alternative method to diagnose novel molecular biomarkers.

In this investigation, we employed the bioinformatics approach to discover the differentially expressed genes between T1D patients and healthy donors. Original expression profiling by high throughput sequencing dataset GSE123658 was downloaded. 39 T1D patients’ samples and 43 healthy donors’ samples were analyzed in our investigation. Commonly altered DEGs were isolated from integrated data. Additionally, GO/ REACTOME pathway analysis, construction of protein–protein interaction network, modules, target gene - miRNA regulatory network and target gene - TF regulatory network analysis were performed to analyze these data. Four hub genes (EGFR, GRIN2B, GJA1, CAP2, MIF, POLR2A, PRKACA, GABARAP, TLN1 and PXN) were identified. ROC (receiver operating characteristic) curve and RT-PCR analysis were used to verify clinically relevant hub genes. The aim of this investigation was to gain a better understanding of the underlying molecular mechanisms and to discover molecular biomarkers for T1D.

## Material and methods

### Data resources

Expression profiling by high throughput sequencing dataset GSE123658 was downloaded from the GEO database (http://www.ncbi.nlm.nih.gov/geo/) [21]. CPM count normalization performed on the original dataset GSE123658 from GEO databse using package edgeR package [22], voom function [23], and Limma [24] of R software. The data was produced using a GPL18573 Illumina NextSeq 500 (Homo sapiens). The GSE123658 dataset contained data from 82 samples, including 39 T1D patients’ samples and 43 healthy donors’ samples.

### Identification of DEGs

The identification of DEGs between 39 T1D patients’ samples and 43 healthy donors’ samples was performed using Limma package in R bioconductor. lmFit function in the limma package to construct linear model for individual gene [25]. makeContrasts function in the limma package to compose similarity between T1D and healthy donors groups (log fold-changes) are obtained as contrasts of these fitted linear model. eBayes is a function in limma package which figure out empirical Bayes predicts of DEGs [26]. topTable function in limma package to obtain a table of the most significant Up and down regulated genes from a eBayes model fit. To correct the discovery of statistically important molecular biomarkers and limitations of false-positives, we using the adjusted P-value and Benjamini and Hochberg false discovery rate method [27]. Fold-change (FC) and adjust p-values were used to found DEGs. A |log2FC|  > 0.94 for up regulated genes, |log2FC|  -0.39 for down regulated genes and *P*-value  <  0.05 were used as considered statistically significant. The volcano plot was implemented using ggplot2 package [28], and the heat map was established using gplots package in R language.

### Gene Ontology (GO) and pathway enrichment analyses of DEGs

Gene Ontology (GO) (http://www.geneontology.org) analysis is a routine analysis for annotating genes and determining biological component, including biological process (BP), cellular component (CC) and molecular function (MF) [29]. REACTOME (https://reactome.org/) [30] pathway database is applied for classification by correlating gene sets into their respective pathways. The ToppGene (ToppFun) (https://toppgene.cchmc.org/enrichment.jsp) [31] is a gene functional classification tool that objective to provide a extensive set of functional annotation tools for authors to recognize the biological explanation behind large lists of genes. *P* < 0.05 was find statistically significant.

### PPI network construction and module analysis

The online Human Integrated Protein-Protein Interaction rEference (HIPPIE) (http://cbdm.uni-mainz.de/hippie/) [32] online database was using to predicted the PPI network information. Analyzing the interactions and functions between DEGs may provide information about the mechanisms of generation and development of disease (PPI score  >  0.4). Cytoscape 3.8.0 (http://www.cytoscape.org/) [33] is a bioinformatics platform for constructing and visualizing molecular interaction networks. The Network Analyzer Cytoscape plug-in was used to find hub genes were screened using highest node degree [34], betweenness centrality [35], stress centrality [36] and closeness centrality [37] methods and hub genes were further analyzed for pathway and GO enrichment analysis. The plug-in PEWCC1 (http://apps.cytoscape.org/apps/PEWCC1) [38] of Cytoscape was applied to detect densely connected regions in PPI networks. The PPI networks were constructed using Cytoscape and the most key module in the PPI networks was preferred using PEWCC1. The criteria for selection were set as follows: Max depth = 100, degree cut-off = 2, Node score cut-off = 0.2, PEWCC1 scores >5, and K-score = 2.

### Construction of miRNA - target regulatory network

miRNet database (https://www.mirnet.ca/) [39] online database was used to predict miRNAs that targeted the DEGs associated with T1D. The DEGs were selected according to the screening criterion of *P* value <0.05. The results were exported into the Cytoscape software for analysis. The target genes - miRNA regulatory network was constructed through network topology prosperities. The node degree was determined using the Network analysis plugin, and miRNAs with a node degree >12.

### Construction of TF - target regulatory network

NetworkAnalyst database (https://www.networkanalyst.ca/) [40] online database was used to predict TFs that targeted the DEGs associated with T1D. The DEGs were selected according to the screening criterion of *P* value <0.05. The results were exported into the Cytoscape software for analysis. The target genes - TF regulatory network was constructed through network topology prosperities. The node degree was determined using the Network analysis plugin, and TFs with a node degree >12.

### Validation of hub genes

Receiver operating characteristic (ROC) curve analysis [41] was implemented to calculate the sensitivity and specificity of the DEGs for T1D diagnosis using the R package by using the generalized linear model (GLM) in machine learning algorithms [42]. An area under the curve (AUC) value was determined and used to label the ROC effect. The RIN-m5F (ATCC CRL-11605) cell line procured from ATCC for T1D. RINm5F cell line was grown in RPMI-1640 medium added with 10% fetal bovine serum and penicillin/streptomycin. Incubate this cell line at 37 °C in a 5% CO2 in humidified cell culture incubator. The HIT­T15 (ATCC CRL­1777) cell line procured from ATCC for normal. CRL-1777 cell line was grown in Ham’s F12K medium added with 10% fetal bovine serum,87.5%; dialyzedhorse serum, 2 mM L­glutamine, 1.5 g/L sodium bicarbonate, and penicillin/streptomycin. Incubate the this cell line at 37 °C in a 5% CO2 in humidified cell culture incubator. In RT-PCR analysis, total RNA was isolated from in vitro cultured cells of diabetics and normal using a TRI Reagent (Sigma, USA). cDNA was synthesized using 2.0 μg of total RNA with the FastQuant RT kit (with gDNase; Tiangen Biotech Co., Ltd.). The relative mRNA expression was measured in the QuantStudio 7 Flex real-time PCR system (Thermo Fisher Scientific, Waltham, MA, USA). The relative expression levels were determined by the 2^-ΔΔCt^ method and normalized to internal control β-actin [42]. All RT-PCR reactions were performed in triplicate. The primers used to explore mRNA expression of ten hub genes were shown in Table 1.

**Table 1:** The sequences of primers for quantitative RT-PCR

Genes	Primers	Length of target fragment, bp
EGFR	F: AGGCACGAGTAACAAGCTCAC R: ATGAGGACATAACCAGCCACC	21 21
GRIN2B	F: TCTGACCGGAAGATCCAGGG R: TCCATGATGTTGAGCATTACGG	20 22
GJA1	F: GGTGACTGGAGCGCCTTAG R: GCGCACATGAGAGATTGGGA	19 20
CAP2	F: CCCTGCCCTTGGATGGATAG R: ACGCTGATACTGTGGATGCTA	20 21
MIF	F: CTCTCCGAGCTCACCCAGCAG R: CGCGTTCATGTCGTAATAGTT	21 21
POLR2A	F: GGGTGGCATCAAATACCCAGA R: AGACACAGCGCAAAACTTTCA	21 21
PRKACA	F: AGCCCACTTGGATCAGTTTGA R: GTTCCCGGTCTCCTTGTGT	21 19
GABARAP	F: AGAAGAGCATCCGTTCGAGAA R: CCAGGTCTCCTATCCGAGCTT	21 21
TLN1	F: GACGATGCAGTTTGAGCCG R: GGTCATCATCTGACAGAAAGAG	19 23
PXN	F: CTGCTGGAACTGAACGCTGTA R: GGGGCTGTTAGTCTCTGGGA	21 20

*F* Forward Primers, *R* Reverse Primers

### Molecular Docking studies

The surflex-docking studies of the designed molecule, standard on over expressed genes of PDB protein were performed using SYBYL-X 2.0 perpetual software. All the designed molecules and standard were outlined using ChemDraw Software, imported and saved in sdf. templet using open babel free software. The protein structures of EGFR (epidermal growth factor receptor) and its co-crystallised protein of PDB code 2XYJ, 2XYX and GRIN2B its co-crystallised protein of PDB code and its co-crystallised protein of PDB code 5EWL, 6E7V were extracted from Protein Data Bank [43–46]. Together with the TRIPOS force field, GasteigerHuckel (GH) charges were added to all designed molecules and standard for the structure optimization process. Additionally, using MMFF94s and MMFF94 algorithm methods, energy minimization was done. The protein preparation was carried out after incorporation of protein. The co-crystallized ligand and all water molecules were removed from the crystal structure; more hydrogen’s were added and the side chain was set. TRIPOS force field was used for the minimization of structure. The compounds’ interaction efficiency with the receptor was represented by the Surflex-Dock score in kcal / mol units. The interaction between the protein and the ligand, the best pose was incorporated into the molecular area. The visualization of ligand interaction with receptor is done by using discovery studio visualizer.

## Results

### Identification of DEGs

**Fig. 1:**
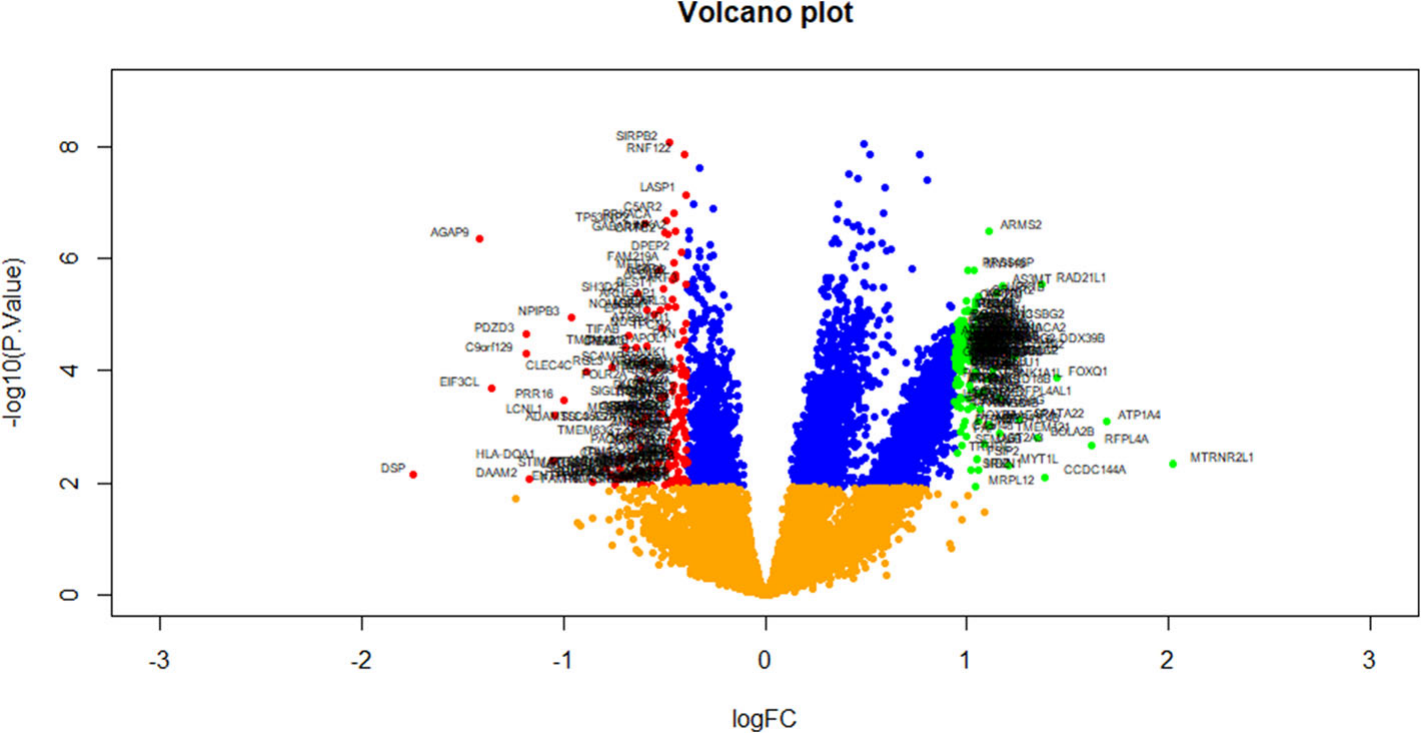
Volcano plot of differentially expressed genes. Genes with a significant change of more than two-fold were selected. Green dot represented up regulated significant genes and red dot represented down regulated significant genes

**Fig. 2:**
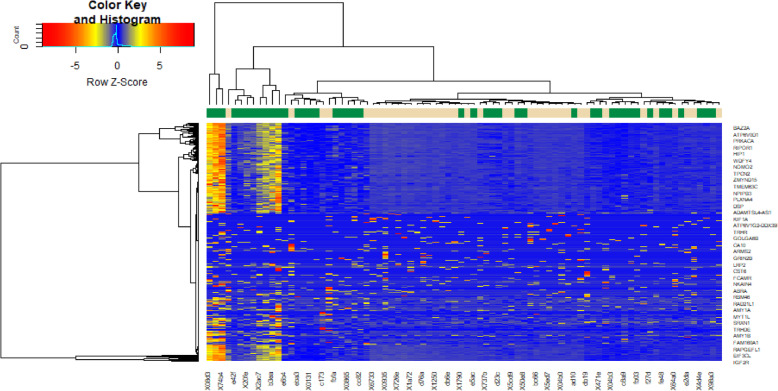
Heat map of differentially expressed genes. Legend on the top left indicate log fold change of genes. (A1 – A43 = healthy donors’ samples; B1 – B14 = T1D samples)

With a threshold of *P*-value <0.05 and absolute value of |log2FC|  > 0.94 for up regulated genes, |log2FC|  -0.39 for down regulated genes, DEGs were identified from dataset. 284 DEGs were screened from the GSE123658 dataset, which consisted of 142 up regulated genes and 142 down regulated genes and only top ten up regulated genes and down regulated genes are listed in Table 2. Volcano plots were used to visualize differential expression of genes between the T1D group and healthy donors group (Fig.1). A heat map showed expression profiling of DEGs in the analysis result (Fig.2).

**Table 2:** The statistical metrics for key differentially expressed genes (DEGs)

GeneSymbol	logFC	***p*** Value	adj. P.Val	t value	Regulations	GeneName	
ARMS2	1.105122	3.28E-07	0.000198	5.53328	Up	age-related maculopathy susceptibility 2	
PRSS46P	1.005863	1.59E-06	0.000459	5.153257	Up	serine protease 46, pseudogene	
MYH13	1.03327	1.64E-06	0.00046	5.144666	Up	myosin heavy chain 13	
RAD21L1	1.367492	2.87E-06	0.000592	5.007555	Up	RAD21 cohesin complex component like 1	
AS3MT	1.172185	2.97E-06	0.000593	4.998809	Up	arsenitemethyltransferase	
UPK1B	1.142189	4.25E-06	0.000663	4.909531	Up	uroplakin 1B	
CRHR2	1.057435	4.66E-06	0.000686	4.886267	Up	corticotropin releasing hormone receptor 2	
KRT20	1.041956	5.47E-06	0.000738	4.845569	Up	keratin 20	
CYP2F1	0.996571	5.49E-06	0.000738	4.844967	Up	cytochrome P450 family 2 subfamily F member 1	
TBX20	0.915545	6.65E-06	0.000852	4.796325	Up	T-box transcription factor 20	
TEX15	1.040483	6.7E-06	0.000852	4.794446	Up	testis expressed 15, meiosis and synapsis associated	
REN	0.918655	7.4E-06	0.00091	4.769059	Up	renin	
RDH8	1.031642	8.2E-06	0.000925	4.743027	Up	retinol dehydrogenase 8	
PADI3	1.004196	8.23E-06	0.000925	4.742198	Up	peptidyl arginine deiminase 3	
RIMS4	1.005408	8.44E-06	0.000925	4.735751	Up	regulating synaptic membrane exocytosis 4	
MS4A5	0.988204	8.45E-06	0.000925	4.735279	Up	membrane spanning 4-domains A5	
HFM1	1.083591	1.04E-05	0.001038	4.683383	Up	helicase for meiosis 1	
EGFR	1.045618	1.16E-05	0.001078	4.655195	Up	epidermal growth factor receptor	
C10orf113	0.984302	1.27E-05	0.001122	4.629975	Up	chromosome 10 open reading frame 113	
RGS4	0.968679	1.28E-05	0.001122	4.629106	Up	regulator of G protein signaling 4	
C2orf73	0.965222	1.29E-05	0.001122	4.626793	Up	chromosome 2 open reading frame 73	
ACSBG2	1.181896	1.27E-05	0.001122	4.631273	Up	acyl-CoA synthetasebubblegum family member 2	
BTBD18	0.963267	1.41E-05	0.001181	4.604073	Up	BTB domain containing 18	
DPPA5	0.954617	1.44E-05	0.001195	4.598614	Up	developmental pluripotency associated 5	
OR10J3	0.985446	1.56E-05	0.001237	4.578119	Up	olfactory receptor family 10 subfamily J member 3	
KCNK4	1.033357	1.57E-05	0.001237	4.575705	Up	potassium two pore domain channel subfamily K member 4	
LRAT	0.925599	1.61E-05	0.001259	4.568727	Up	lecithin retinol acyltransferase	
IL17B	0.958598	1.72E-05	0.001302	4.551641	Up	interleukin 17B	
FBXO10	1.012141	1.76E-05	0.001319	4.545579	Up	F-box protein 10	
LRRC74A	0.894289	1.78E-05	0.001319	4.54289	Up	leucine rich repeat containing 74A	
TM4SF5	0.988935	1.86E-05	0.001335	4.531217	Up	transmembrane 4 L six family member 5	
OR2T29	0.956232	1.89E-05	0.001335	4.527962	Up	olfactory receptor family 2 subfamily T member 29	
RNF180	1.050934	1.89E-05	0.001335	4.526775	Up	ring finger protein 180	
MYOT	0.998153	1.9E-05	0.001335	4.525922	Up	myotilin	
SLC22A25	0.905085	2.1E-05	0.001407	4.499193	Up	solute carrier family 22 member 25	
CMA1	0.871927	2.12E-05	0.001407	4.497403	Up	chymase 1	
PCDHB10	1.017337	2.14E-05	0.001407	4.494598	Up	protocadherin beta 10	
HRCT1	0.931584	2.16E-05	0.001407	4.492152	Up	histidine rich carboxyl terminus 1	
TRHR	0.967196	2.17E-05	0.001407	4.491435	Up	thyrotropin releasing hormone receptor	
NACA2	1.235555	2.15E-05	0.001407	4.494057	Up	nascent polypeptide associated complex subunit alpha 2	
CCL19	1.05675	2.17E-05	0.001407	4.490985	Up	C-C motif chemokine ligand 19	
DMRTA2	0.919949	2.31E-05	0.001446	4.474549	Up	DMRT like family A2	
NRAP	0.970444	2.42E-05	0.001479	4.46211	Up	nebulin related anchoring protein	
DNAH3	0.948597	2.46E-05	0.001483	4.458656	Up	dynein axonemal heavy chain 3	
CCL13	1.008793	2.46E-05	0.001483	4.458373	Up	C-C motif chemokine ligand 13	
OSR1	0.99949	2.47E-05	0.001483	4.456576	Up	odd-skipped related transcription factor 1	
TMEM145	0.966024	2.46E-05	0.001483	4.457613	Up	transmembrane protein 145	
AVP	0.940076	2.5E-05	0.00149	4.453465	Up	arginine vasopressin	
CSHL1	0.84012	2.55E-05	0.001496	4.44882	Up	chorionic somatomammotropin hormone like 1	
LHFPL3	1.029874	2.56E-05	0.001497	4.44723	Up	LHFPL tetraspan subfamily member 3	
SNAP91	1.023292	2.57E-05	0.001497	4.446795	Up	synaptosome associated protein 91	
SOX18	1.029443	2.6E-05	0.001504	4.44371	Up	SRY-box transcription factor 18	
CAP2	1.0769	2.63E-05	0.001511	4.440701	Up	cyclase associated actin cytoskeleton regulatory protein 2	
C1orf146	0.912546	2.69E-05	0.001529	4.434806	Up	chromosome 1 open reading frame 146	
PDE6C	0.880944	2.82E-05	0.001574	4.421773	Up	phosphodiesterase 6C	
CRYAA	0.894932	2.89E-05	0.001596	4.41523	Up	crystallin alpha A	
NR0B2	0.903675	2.9E-05	0.001596	4.414618	Up	nuclear receptor subfamily 0 group B member 2	
ANKRD30B	0.953544	2.93E-05	0.001599	4.411547	Up	ankyrin repeat domain 30B	
CST6	0.946705	3.04E-05	0.001632	4.401733	Up	cystatin E/M	
CAMK1G	0.956887	3.05E-05	0.001632	4.401395	Up	calcium/calmodulin dependent protein kinase IG	
CYP39A1	0.879266	3.08E-05	0.001632	4.399046	Up	cytochrome P450 family 39 subfamily A member 1	
ZNF214	0.948008	3.14E-05	0.001653	4.393435	Up	zinc finger protein 214	
MRGPRF	0.936902	3.18E-05	0.001666	4.390434	Up	MAS related GPR family member F	
PTPRT	1.102577	3.22E-05	0.001674	4.386782	Up	protein tyrosine phosphatase receptor type T	
KRT39	1.081629	3.21E-05	0.001674	4.387502	Up	keratin 39	
PHF21B	0.897853	3.26E-05	0.001681	4.383695	Up	PHD finger protein 21B	
ABRA	0.967897	3.26E-05	0.001681	4.383147	Up	actin binding Rho activating protein	
ADGRF4	0.958187	3.3E-05	0.001693	4.380467	Up	adhesion G protein-coupled receptor F4	
ABCC12	0.932702	3.35E-05	0.001709	4.376353	Up	ATP binding cassette subfamily C member 12	
SLC16A8	0.908131	3.38E-05	0.001712	4.374274	Up	solute carrier family 16 member 8	
ATP6V1G2-DDX39B	0.990995	3.4E-05	0.001715	4.372164	Up	ATP6V1G2-DDX39B readthrough (NMD candidate)	
LEMD1	0.846889	3.51E-05	0.001738	4.363856	Up	LEM domain containing 1	
KPNA7	0.953213	3.51E-05	0.001738	4.363815	Up	karyopherin subunit alpha 7	
MYH6	0.952519	3.56E-05	0.001753	4.359936	Up	myosin heavy chain 6	
FRMPD2	0.957963	3.68E-05	0.001757	4.351517	Up	FERM and PDZ domain containing 2	
SULT6B1	0.965243	3.69E-05	0.001757	4.350796	Up	sulfotransferase family 6B member 1	
HSD3B2	0.933208	3.7E-05	0.001757	4.3496	Up	hydroxy-delta-5-steroid dehydrogenase, 3 beta- and steroid delta-isomerase 2	
SULF1	0.9309	3.71E-05	0.001757	4.349307	Up	sulfatase 1	
G6PC	1.038462	3.71E-05	0.001757	4.349175	Up	glucose-6-phosphatase catalytic subunit	
MTRNR2L6	1.015308	3.72E-05	0.001757	4.348247	Up	MT-RNR2 like 6	
IZUMO1	0.863468	3.73E-05	0.001757	4.347643	Up	izumo sperm-egg fusion 1	
CFAP77	0.854205	3.75E-05	0.001757	4.346388	Up	cilia and flagella associated protein 77	
GPR37	0.969013	3.75E-05	0.001757	4.346128	Up	G protein-coupled receptor 37	
OR6C70	0.996132	3.78E-05	0.001757	4.344383	Up	olfactory receptor family 6 subfamily C member 70	
URGCP-MRPS24	0.929538	3.8E-05	0.001759	4.342672	Up	URGCP-MRPS24 readthrough	
SLC26A3	0.912109	3.84E-05	0.001766	4.33951	Up	solute carrier family 26 member 3	
ITGBL1	0.89074	3.88E-05	0.001766	4.337347	Up	integrin subunit beta like 1	
LOC102725072	0.93312	3.88E-05	0.001766	4.336863	Up	Putative uncharacterized protein DKFZp434K191	
BLACE	0.837756	3.94E-05	0.001771	4.332862	Up	B cell acute lymphoblastic leukemia expressed	
LOC100131496	0.873058	3.96E-05	0.001771	4.331895	Up	uncharacterized LOC100131496	
LIPF	0.971728	3.97E-05	0.001771	4.331252	Up	lipase F, gastric type	
FNDC8	0.897073	4E-05	0.001771	4.3287	Up	fibronectin type III domain containing 8	
LOC389199	0.873159	4.1E-05	0.001785	4.322016	Up	uncharacterized LOC389199	
OR2K2	0.856644	4.28E-05	0.001834	4.310597	Up	olfactory receptor family 2 subfamily K member 2	
FABP6	0.940984	4.31E-05	0.001842	4.308644	Up	fatty acid binding protein 6	
SPATA16	0.946274	4.37E-05	0.001845	4.305413	Up	spermatogenesis associated 16	
SMCO1	1.042857	4.36E-05	0.001845	4.305897	Up	single-pass membrane protein with coiled-coil domains 1	
PCDHB2	1.169306	4.41E-05	0.001855	4.302798	Up	protocadherin beta 2	
FAM243A	0.902671	4.49E-05	0.001866	4.29812	Up	family with sequence similarity 243 member A	
HOXB8	0.886424	4.51E-05	0.001866	4.296412	Up	homeobox B8	
LMO1	0.892873	4.53E-05	0.00187	4.29529	Up	LIM domain only 1	
MAS1L	0.89613	4.56E-05	0.001876	4.293436	Up	MAS1 proto-oncogene like, G protein-coupled receptor	
GLYATL3	0.90734	4.59E-05	0.001876	4.291841	Up	glycine-N-acyltransferase like 3	
UGT2A1	0.870199	4.69E-05	0.001883	4.286149	Up	UDP glucuronosyltransferase family 2 member A1 complex locus	
RERG	0.939549	4.69E-05	0.001883	4.286306	Up	RAS like estrogen regulated growth inhibitor	
CELA2A	0.924629	4.7E-05	0.001883	4.285588	Up	chymotrypsin like elastase 2A	
LRFN5	0.946274	4.8E-05	0.001915	4.279702	Up	leucine rich repeat and fibronectin type III domain containing 5	
PAX3	0.826938	4.83E-05	0.001921	4.27827	Up	paired box 3	
KIF1A	0.973095	4.86E-05	0.001927	4.276802	Up	kinesin family member 1A	
MRGPRE	0.86341	4.93E-05	0.001952	4.272623	Up	MAS related GPR family member E	
NPY	0.877892	4.99E-05	0.001962	4.269525	Up	neuropeptide Y	
EFS	1.003465	5E-05	0.001962	4.269001	Up	embryonal Fyn-associated substrate	
ZSCAN1	0.921942	5.02E-05	0.001962	4.26778	Up	zinc finger and SCAN domain containing 1	
MEIOB	0.971239	5.03E-05	0.001962	4.267016	Up	meiosis specific with OB-fold	
TMPRSS15	0.884284	5.02E-05	0.001962	4.267806	Up	transmembrane serine protease 15	
DRD2	0.861436	5.04E-05	0.001962	4.266826	Up	dopamine receptor D2	
CNMD	0.959271	5.05E-05	0.001963	4.266074	Up	chondromodulin	
AMBP	0.883904	5.08E-05	0.001966	4.264596	Up	alpha-1-microglobulin/bikunin precursor	
C8orf34	0.874882	5.16E-05	0.00198	4.260541	Up	chromosome 8 open reading frame 34	
EGR4	0.93485	5.19E-05	0.001987	4.25858	Up	early growth response 4	
LGALS7	0.958985	5.24E-05	0.001987	4.255983	Up	galectin 7	
INHA	0.858348	5.23E-05	0.001987	4.256562	Up	inhibin subunit alpha	
MNX1	0.845332	5.28E-05	0.00199	4.254289	Up	motor neuron and pancreas homeobox 1	
SLC10A1	0.889098	5.41E-05	0.002017	4.247721	Up	solute carrier family 10 member 1	
NPBWR1	0.965049	5.42E-05	0.002017	4.246995	Up	neuropeptides B and W receptor 1	
PMF1-BGLAP	0.976467	5.45E-05	0.002025	4.245385	Up	PMF1-BGLAP readthrough	
LHCGR	0.897295	5.57E-05	0.002041	4.239485	Up	luteinizing hormone/choriogonadotropin receptor	
NT5C1A	0.86506	5.59E-05	0.002041	4.238871	Up	5′-nucleotidase, cytosolic IA	
ASCL1	0.918002	5.6E-05	0.002041	4.238038	Up	achaete-scute family bHLH transcription factor 1	
CCDC140	0.969595	5.61E-05	0.002041	4.237583	Up	coiled-coil domain containing 140	
NEUROG3	0.951564	5.79E-05	0.002073	4.229287	Up	neurogenin 3	
TMC2	1.236136	5.62E-05	0.002041	4.237443	Up	transmembrane channel like 2	
MINDY4B	1.034321	5.81E-05	0.002073	4.228387	Up	MINDY family member 4B	
COL3A1	0.899332	5.86E-05	0.002073	4.226076	Up	collagen type III alpha 1 chain	
SLCO6A1	0.920379	5.89E-05	0.002073	4.224473	Up	solute carrier organic anion transporter family member 6A1	
THRSP	0.891603	5.93E-05	0.002073	4.222727	Up	thyroid hormone responsive	
DBX2	0.883726	5.93E-05	0.002073	4.222452	Up	developing brain homeobox 2	
OR52N5	0.874656	5.94E-05	0.002073	4.221942	Up	olfactory receptor family 52 subfamily N member 5	
ANO4	0.891458	5.95E-05	0.002073	4.22163	Up	anoctamin 4	
RPRML	1.013736	5.96E-05	0.002073	4.22104	Up	reprimo like	
LIPM	0.857463	5.97E-05	0.002073	4.220906	Up	lipase family member M	
EFCAB3	0.930354	5.98E-05	0.002073	4.220334	Up	EF-hand calcium binding domain 3	
LRRC2-AS1	0.891603	5.99E-05	0.002073	4.220002	Up	LRRC2 antisense RNA 1	
GJA1	0.940111	5.94E-05	0.002073	4.222116	Up	gap junction protein alpha 1	
OR51E1	0.966601	6.13E-05	0.002103	4.21374	Up	olfactory receptor family 51 subfamily E member 1	
ANP32D	0.855187	6.14E-05	0.002103	4.213256	Up	acidic nuclear phosphoprotein 32 family member D	
FMC1-LUC7L2	0.914577	6.21E-05	0.002109	4.21018	Up	FMC1-LUC7L2 readthrough	
PRLHR	0.905404	6.26E-05	0.002114	4.207869	Up	prolactin releasing hormone receptor	
PPFIA2	0.962581	6.28E-05	0.002114	4.206985	Up	PTPRF interacting protein alpha 2	
IRX5	0.930915	6.35E-05	0.002124	4.203899	Up	iroquoishomeobox 5	
CSRNP3	0.893691	6.37E-05	0.002125	4.20305	Up	cysteine and serine rich nuclear protein 3	
DAND5	0.869683	6.39E-05	0.002125	4.20215	Up	DAN domain BMP antagonist family member 5	
NKAIN4	0.986106	6.38E-05	0.002125	4.202648	Up	sodium/potassium transporting ATPase interacting 4	
TMPRSS11B	0.859699	6.45E-05	0.002126	4.19958	Up	transmembrane serine protease 11B	
GULP1	0.98403	6.48E-05	0.002126	4.198514	Up	GULP PTB domain containing engulfment adaptor 1	
ADGRL4	0.967777	6.53E-05	0.002135	4.196582	Up	adhesion G protein-coupled receptor L4	
HOXD8	0.897017	6.6E-05	0.002143	4.193495	Up	homeobox D8	
OR52N1	0.839763	6.66E-05	0.002154	4.191133	Up	olfactory receptor family 52 subfamily N member 1	
LINC01555	0.897816	6.66E-05	0.002154	4.190957	Up	long intergenic non-protein coding RNA 1555	
CA10	1.011486	6.89E-05	0.002212	4.181597	Up	carbonic anhydrase 10	
CYP2W1	0.875193	7.07E-05	0.002224	4.174858	Up	cytochrome P450 family 2 subfamily W member 1	
CCDC27	0.885529	7.08E-05	0.002224	4.174477	Up	coiled-coil domain containing 27	
MYH7	0.885529	7.08E-05	0.002224	4.174477	Up	myosin heavy chain 7	
GNAT1	0.885529	7.08E-05	0.002224	4.174477	Up	G protein subunit alpha transducin 1	
NPPC	0.964362	7.36E-05	0.002263	4.163694	Up	natriuretic peptide C	
CBLC	0.891165	7.5E-05	0.002292	4.158584	Up	Cbl proto-oncogene C	
TRIM55	0.945582	7.46E-05	0.002284	4.160085	Up	tripartite motif containing 55	
EEF1G	0.83887	7.57E-05	0.002293	4.155878	Up	eukaryotic translation elongation factor 1 gamma	
ADCYAP1R1	0.896389	7.58E-05	0.002293	4.155685	Up	ADCYAP receptor type I	
TAFA4	0.838908	7.58E-05	0.002293	4.155485	Up	TAFA chemokine like family member 4	
OVOL1	0.884916	7.57E-05	0.002293	4.156118	Up	ovo like transcriptional repressor 1	
EFEMP1	0.913807	7.67E-05	0.002304	4.152284	Up	EGF containing fibulin extracellular matrix protein 1	
SLC36A2	0.897775	7.69E-05	0.002304	4.151795	Up	solute carrier family 36 member 2	
SPINK13	0.860333	7.76E-05	0.002322	4.149133	Up	serine peptidase inhibitor, Kazal type 13 (putative)	
CCDC158	0.832487	7.97E-05	0.002364	4.141709	Up	coiled-coil domain containing 158	
RBAK-RBAKDN	0.916027	8.11E-05	0.002396	4.137123	Up	RBAK-RBAKDN readthrough	
CCL17	0.896315	8.25E-05	0.002434	4.132223	Up	C-C motif chemokine ligand 17	
SELE	0.850167	8.29E-05	0.002443	4.130829	Up	selectin E	
GOLGA6B	0.9457	8.37E-05	0.002446	4.128465	Up	golgin A6 family member B	
IGFL4	0.850147	8.44E-05	0.002452	4.125895	Up	IGF like family member 4	
CNTFR	0.883786	8.48E-05	0.002457	4.124845	Up	ciliaryneurotrophic factor receptor	
C1orf141	0.949302	8.51E-05	0.00246	4.123621	Up	chromosome 1 open reading frame 141	
NOS1	0.932437	8.63E-05	0.002471	4.119973	Up	nitric oxide synthase 1	
REG1B	0.893055	8.76E-05	0.002495	4.115883	Up	regenerating family member 1 beta	
CAPSL	0.894376	8.79E-05	0.002501	4.114845	Up	calcyphosine like	
C1QTNF7	0.883655	8.86E-05	0.002513	4.112561	Up	C1q and TNF related 7	
RHBDL2	0.883638	8.88E-05	0.002513	4.111975	Up	rhomboid like 2	
TEAD4	0.893342	8.91E-05	0.002513	4.111195	Up	TEA domain transcription factor 4	
PRRX1	0.923623	8.93E-05	0.002513	4.110382	Up	paired related homeobox 1	
SERPINB12	0.833849	8.95E-05	0.002513	4.109731	Up	serpin family B member 12	
TBX10	0.833115	9E-05	0.002513	4.108444	Up	T-box transcription factor 10	
COL6A5	0.920311	9.01E-05	0.002513	4.108069	Up	collagen type VI alpha 5 chain	
KLK4	0.837318	9.03E-05	0.002513	4.107393	Up	kallikrein related peptidase 4	
GRIN2B	0.950071	9.06E-05	0.002513	4.106573	Up	glutamate ionotropic receptor NMDA type subunit 2B	
RGS20	0.900519	9.07E-05	0.002513	4.106097	Up	regulator of G protein signaling 20	
ZNF728	0.883605	9.14E-05	0.002515	4.103954	Up	zinc finger protein 728	
SIX4	0.85187	9.18E-05	0.002518	4.102815	Up	SIX homeobox 4	
NPFFR2	0.929088	9.21E-05	0.002521	4.101991	Up	neuropeptide FF receptor 2	
ANKRD62	0.899096	9.23E-05	0.002521	4.101348	Up	ankyrin repeat domain 62	
CLLU1	1.132069	9.03E-05	0.002513	4.10742	Up	chronic lymphocytic leukemia up-regulated 1	
CNTNAP4	0.914215	9.32E-05	0.002528	4.09853	Up	contactin associated protein like 4	
COL12A1	0.912316	9.56E-05	0.002587	4.091597	Up	collagen type XII alpha 1 chain	
EBF3	0.839069	9.7E-05	0.002604	4.087742	Up	EBF transcription factor 3	
GRIA4	0.979336	9.8E-05	0.002614	4.084733	Up	glutamate ionotropic receptor AMPA type subunit 4	
PTHLH	0.963725	9.8E-05	0.002614	4.08484	Up	parathyroid hormone like hormone	
SLC9A2	0.881992	9.82E-05	0.002614	4.084262	Up	solute carrier family 9 member A2	
HEPHL1	0.882607	9.86E-05	0.002619	4.083035	Up	hephaestin like 1	
SCG3	0.893171	9.91E-05	0.00262	4.081758	Up	secretogranin III	
SIX1	0.921197	9.92E-05	0.00262	4.081507	Up	SIX homeobox 1	
TSPYL6	0.891306	9.97E-05	0.002628	4.07987	Up	TSPY like 6	
IRGC	0.839346	0.0001	0.002634	4.078065	Up	immunity related GTPase cinema	
GPR6	0.930096	0.000101	0.00264	4.076739	Up	G protein-coupled receptor 6	
SCN3B	0.832646	0.000101	0.00264	4.076478	Up	sodium voltage-gated channel beta subunit 3	
EMILIN3	0.967257	0.000102	0.002653	4.074049	Up	elastin microfibrilinterfacer 3	
EPN3	0.914055	0.000104	0.002683	4.068937	Up	epsin 3	
PDLIM4	0.933994	0.000104	0.002683	4.068455	Up	PDZ and LIM domain 4	
DCC	1.037614	0.000106	0.002705	4.06329	Up	DCC netrin 1 receptor	
IL9	0.854817	0.000107	0.002723	4.060443	Up	interleukin 9	
GRM5	0.826169	0.000108	0.002744	4.057559	Up	glutamate metabotropic receptor 5	
ODF3L2	0.894219	0.000108	0.002744	4.057227	Up	outer dense fiber of sperm tails 3 like 2	
C5orf60	0.929657	0.000113	0.002843	4.044374	Up	chromosome 5 open reading frame 60	
LRRC3B	0.885916	0.000114	0.002843	4.042437	Up	leucine rich repeat containing 3B	
NLRP14	0.859959	0.000114	0.002843	4.043418	Up	NLR family pyrin domain containing 14	
SLC25A51P4	0.889805	0.000117	0.002902	4.035322	Up	SLC25A51 pseudogene 4	
FOXD4L4	0.839378	0.00012	0.002931	4.028918	Up	forkhead box D4 like 4	
PGR	0.831922	0.00012	0.002931	4.028878	Up	progesterone receptor	
PRL	1.125879	0.000118	0.002906	4.034159	Up	prolactin	
C3orf79	0.857368	0.000121	0.00294	4.025807	Up	chromosome 3 open reading frame 79	
SERPIND1	0.906509	0.000121	0.002937	4.0271	Up	serpin family D member 1	
S100A7A	0.847703	0.000122	0.002953	4.023133	Up	S100 calcium binding protein A7A	
EN1	0.9169	0.000124	0.002964	4.018471	Up	engrailed homeobox 1	
KRT38	1.042088	0.000125	0.002971	4.016678	Up	keratin 38	
LINC02108	0.846143	0.000126	0.002975	4.014499	Up	long intergenic non-protein coding RNA 2108	
GPR158	0.831736	0.000126	0.002975	4.013975	Up	G protein-coupled receptor 158	
ACSM6	0.908554	0.000128	0.002993	4.011439	Up	acyl-CoA synthetase medium chain family member 6	
ASPA	0.871659	0.000129	0.003	4.007633	Up	aspartoacylase	
SH3GL2	0.918924	0.000132	0.003024	4.001385	Up	SH3 domain containing GRB2 like 2, endophilin A1	
HAO1	0.826311	0.000133	0.00303	4.000253	Up	hydroxyacid oxidase 1	
PDPN	0.889264	0.000134	0.003031	3.996743	Up	podoplanin	
CWH43	0.839103	0.000135	0.003032	3.996334	Up	cell wall biogenesis 43 C-terminal homolog	
OR6S1	0.835524	0.000135	0.003042	3.994961	Up	olfactory receptor family 6 subfamily S member 1	
OR51B4	0.827743	0.000137	0.003053	3.992138	Up	olfactory receptor family 51 subfamily B member 4	
NPBWR2	0.868277	0.000138	0.003069	3.989344	Up	neuropeptides B and W receptor 2	
THSD4	0.846128	0.000138	0.003069	3.988521	Up	thrombospondin type 1 domain containing 4	
ADAMTS9	0.834461	0.000139	0.003069	3.988039	Up	ADAM metallopeptidase with thrombospondin type 1 motif 9	
FOXQ1	1.443796	0.000136	0.003044	3.993401	Up	forkhead box Q1	
PLN	0.949551	0.000139	0.00307	3.986876	Up	phospholamban	
MAP3K19	0.87061	0.000141	0.00309	3.982479	Up	mitogen-activated protein kinase kinasekinase 19	
CSNK1A1L	1.098186	0.000141	0.00309	3.983183	Up	casein kinase 1 alpha 1 like	
BARX2	0.902302	0.000142	0.003091	3.981995	Up	BARX homeobox 2	
ISY1-RAB43	0.930431	0.000143	0.003092	3.980065	Up	ISY1-RAB43 readthrough	
NELL1	0.869267	0.000146	0.003138	3.972653	Up	neural EGFL like 1	
WT1	0.833065	0.000148	0.003154	3.968739	Up	WT1 transcription factor	
CTCFL	0.834806	0.000151	0.003178	3.963845	Up	CCCTC-binding factor like	
REG1A	0.848322	0.000151	0.003178	3.962989	Up	regenerating family member 1 alpha	
CELA2B	0.861666	0.000153	0.003182	3.960649	Up	chymotrypsin like elastase 2B	
PRELP	0.854017	0.000153	0.003182	3.960358	Up	proline and arginine rich end leucine rich repeat protein	
CNTN2	0.848239	0.000154	0.003184	3.958439	Up	contactin 2	
CRP	0.836218	0.000154	0.003184	3.958316	Up	C-reactive protein	
GGTLC2	0.890003	0.000157	0.003207	3.953599	Up	gamma-glutamyltransferase light chain 2	
FSD2	0.856494	0.000156	0.003207	3.953925	Up	fibronectin type III and SPRY domain containing 2	
MYH4	0.862217	0.000157	0.003207	3.953188	Up	myosin heavy chain 4	
TUBA3C	0.881504	0.00016	0.003247	3.947266	Up	tubulin alpha 3c	
HSD11B2	0.865869	0.000164	0.003268	3.941252	Up	hydroxysteroid 11-beta dehydrogenase 2	
MKRN2OS	0.852903	0.000164	0.003273	3.940266	Up	MKRN2 opposite strand	
SYT4	0.859298	0.000164	0.003275	3.939713	Up	synaptotagmin 4	
TM4SF20	0.900136	0.000167	0.003292	3.934626	Up	transmembrane 4 L six family member 20	
LRP2	0.963595	0.000167	0.003292	3.935053	Up	LDL receptor related protein 2	
ADAM7	0.872506	0.00017	0.00331	3.930757	Up	ADAM metallopeptidase domain 7	
GPR15	0.902774	0.000212	0.003705	3.867416	Up	G protein-coupled receptor 15	
SMTNL2	0.867183	0.000173	0.00334	3.92602	Up	smoothelin like 2	
APOH	0.921926	0.000173	0.00334	3.924941	Up	apolipoprotein H	
FCAMR	0.946932	0.000174	0.003343	3.924287	Up	Fc fragment of IgA and IgM receptor	
TRPA1	0.914295	0.000175	0.003346	3.922484	Up	transient receptor potential cation channel subfamily A member 1	
WIF1	0.847809	0.000176	0.00336	3.919958	Up	WNT inhibitory factor 1	
CLDN20	0.883524	0.000177	0.003361	3.919207	Up	claudin 20	
OPRM1	0.913422	0.000177	0.003361	3.919346	Up	opioid receptor mu 1	
CCDC198	0.938346	0.000177	0.003364	3.918639	Up	coiled-coil domain containing 198	
FREM3	0.956335	0.000182	0.003437	3.910621	Up	FRAS1 related extracellular matrix 3	
ANKRD18B	1.013694	0.000183	0.003441	3.908794	Up	ankyrin repeat domain 18B	
AMY1A	0.94182	0.000183	0.003441	3.908943	Up	amylase alpha 1A	
C2orf80	0.907847	0.000188	0.003491	3.902387	Up	chromosome 2 open reading frame 80	
SP9	0.90324	0.000188	0.003492	3.901414	Up	Sp9 transcription factor	
SLCO1C1	0.9077	0.000194	0.003562	3.89294	Up	solute carrier organic anion transporter family member 1C1	
RFX6	0.897237	0.000195	0.003562	3.891995	Up	regulatory factor X6	
MRO	0.892061	0.000195	0.003563	3.891612	Up	maestro	
THSD7B	0.829227	0.000196	0.003567	3.890001	Up	thrombospondin type 1 domain containing 7B	
C11orf44	0.825633	0.000197	0.003573	3.888583	Up	chromosome 11 open reading frame 44	
PDE11A	0.882109	0.0002	0.003608	3.88364	Up	phosphodiesterase 11A	
BTC	0.842319	0.000202	0.003619	3.881898	Up	betacellulin	
C14orf39	0.852061	0.000202	0.003625	3.881117	Up	chromosome 14 open reading frame 39	
KCNIP1	0.831954	0.000203	0.003634	3.879927	Up	potassium voltage-gated channel interacting protein 1	
DDC	0.899141	0.000205	0.003655	3.876928	Up	dopa decarboxylase	
KRTAP10–5	0.83581	0.000206	0.003664	3.875023	Up	keratin associated protein 10–5	
B3GALT1	0.865828	0.000207	0.003672	3.874106	Up	beta-1,3-galactosyltransferase 1	
KCNT2	0.882533	0.000209	0.003692	3.871085	Up	potassium sodium-activated channel subfamily T member 2	
OTOGL	1.017886	0.000211	0.003705	3.868493	Up	otogelin like	
ELAVL3	0.837778	0.000213	0.00371	3.86572	Up	ELAV like RNA binding protein 3	
CFHR1	0.865906	0.000214	0.003716	3.864405	Up	complement factor H related 1	
PLA2G5	0.83062	0.000216	0.00373	3.862104	Up	phospholipase A2 group V	
FOLH1	0.917208	0.000217	0.003735	3.861207	Up	folate hydrolase 1	
LPA	0.875646	0.000221	0.003777	3.856187	Up	lipoprotein(a)	
LOC100130449	0.831124	0.000221	0.003777	3.855362	Up	uncharacterized LOC100130449	
LINC00923	0.918504	0.000222	0.003777	3.854828	Up	long intergenic non-protein coding RNA 923	
TERB2	0.876286	0.000222	0.003777	3.854536	Up	telomere repeat binding bouquet formation protein 2	
HHIP	0.825721	0.000222	0.003777	3.854168	Up	hedgehog interacting protein	
DRGX	0.842289	0.000228	0.003835	3.846389	Up	dorsal root ganglia homeobox	
SERPINA9	0.84755	0.000228	0.003835	3.846199	Up	serpin family A member 9	
PRKAA2	0.894063	0.000233	0.003871	3.840763	Up	protein kinase AMP-activated catalytic subunit alpha 2	
MMP27	0.867836	0.000236	0.003895	3.837287	Up	matrix metallopeptidase 27	
NPNT	0.90569	0.000236	0.003899	3.836429	Up	nephronectin	
SLC51B	0.839976	0.000238	0.003904	3.834684	Up	solute carrier family 51 beta subunit	
C3orf80	0.904281	0.000236	0.003895	3.836993	Up	chromosome 3 open reading frame 80	
SOX2	0.869644	0.000238	0.003904	3.834656	Up	SRY-box transcription factor 2	
EYA1	1.078018	0.000237	0.003901	3.835456	Up	EYA transcriptional coactivator and phosphatase 1	
SI	0.880135	0.00024	0.003926	3.831805	Up	sucrase-isomaltase	
TIMP4	0.90339	0.000241	0.00393	3.83074	Up	TIMP metallopeptidase inhibitor 4	
LARP6	0.890316	0.00024	0.003926	3.831332	Up	La ribonucleoprotein 6, translational regulator	
MOGAT2	0.833587	0.000243	0.003943	3.828618	Up	monoacylglycerol O-acyltransferase 2	
OR1L3	0.835533	0.000243	0.003943	3.82828	Up	olfactory receptor family 1 subfamily L member 3	
CACNA1G	0.912907	0.000243	0.003943	3.828145	Up	calcium voltage-gated channel subunit alpha1 G	
CRX	0.89157	0.000243	0.003944	3.8278	Up	cone-rod homeobox	
PPP2R2C	0.882852	0.000247	0.00397	3.824007	Up	protein phosphatase 2 regulatory subunit Bgamma	
SLC30A3	0.892961	0.000248	0.003978	3.822534	Up	solute carrier family 30 member 3	
RHOJ	0.860054	0.000252	0.004016	3.81792	Up	ras homolog family member J	
KCNJ13	0.853986	0.000256	0.004054	3.813553	Up	potassium inwardly rectifying channel subfamily J member 13	
HAS2	0.882356	0.000257	0.004062	3.812592	Up	hyaluronan synthase 2	
KRTAP10–6	0.879999	0.000257	0.004062	3.811877	Up	keratin associated protein 10–6	
FFAR1	0.88097	0.000257	0.004062	3.812033	Up	free fatty acid receptor 1	
FGF6	0.835818	0.000259	0.004077	3.810292	Up	fibroblast growth factor 6	
OTX2	1.0534	0.000267	0.004169	3.800608	Up	orthodenticlehomeobox 2	
CLRN2	0.823406	0.000269	0.004182	3.798898	Up	clarin 2	
C19orf81	0.847143	0.000272	0.004205	3.795697	Up	chromosome 19 open reading frame 81	
NUPR2	0.908781	0.000272	0.004205	3.796129	Up	nuclear protein 2, transcriptional regulator	
CCN1	0.860705	0.000276	0.00423	3.791036	Up	cellular communication network factor 1	
HECW1	0.933254	0.000278	0.004243	3.789303	Up	HECT, C2 and WW domain containing E3 ubiquitin protein ligase 1	
PTGER1	0.840067	0.000279	0.004243	3.788337	Up	prostaglandin E receptor 1	
CDH15	0.86729	0.00028	0.004248	3.787447	Up	cadherin 15	
CLVS2	0.851515	0.000281	0.004253	3.786814	Up	clavesin 2	
LY6G6F-LY6G6D	0.856601	0.000281	0.004261	3.78581	Up	LY6G6F-LY6G6D readthrough	
CYP4X1	0.824815	0.000288	0.004307	3.779012	Up	cytochrome P450 family 4 subfamily X member 1	
MYOG	0.879018	0.000289	0.004307	3.778098	Up	myogenin	
RFPL4AL1	1.159429	0.000295	0.00436	3.772636	Up	ret finger protein like 4A like 1	
LRRC8E	0.830259	0.0003	0.004418	3.767782	Up	leucine rich repeat containing 8 VRAC subunit E	
NXPE1	0.860153	0.000302	0.004436	3.765215	Up	neurexophilin and PC-esterase domain family member 1	
KIF25	0.853635	0.000304	0.004441	3.763906	Up	kinesin family member 25	
CBLN2	0.966722	0.000305	0.004454	3.762634	Up	cerebellin 2 precursor	
AREG	0.901451	0.000308	0.004485	3.759795	Up	amphiregulin	
SPATA8	0.878422	0.000311	0.004508	3.7569	Up	spermatogenesis associated 8	
GCGR	0.86834	0.000311	0.004508	3.756813	Up	glucagon receptor	
OR51E2	1.003339	0.000313	0.004529	3.754696	Up	olfactory receptor family 51 subfamily E member 2	
RPE65	0.835844	0.000315	0.004549	3.752653	Up	retinoid isomerohydrolase RPE65	
LAMA4	0.925931	0.000318	0.004572	3.750191	Up	laminin subunit alpha 4	
GABRA4	0.9215	0.000322	0.004599	3.747009	Up	gamma-aminobutyric acid type A receptor alpha4 subunit	
MFAP2	0.972218	0.000324	0.004611	3.74445	Up	microfibril associated protein 2	
WFDC5	0.84266	0.000328	0.004639	3.740917	Up	WAP four-disulfide core domain 5	
IGSF21	0.851515	0.000331	0.004652	3.738722	Up	immunoglobin superfamily member 21	
C17orf102	0.839208	0.000332	0.004669	3.737396	Up	chromosome 17 open reading frame 102	
A2ML1	0.846661	0.000334	0.004675	3.73642	Up	alpha-2-macroglobulin like 1	
PLA2G3	0.828347	0.000334	0.004675	3.73607	Up	phospholipase A2 group III	
C1orf229	0.851752	0.000335	0.004681	3.735201	Up	chromosome 1 open reading frame 229	
PLA2G10	0.831528	0.000345	0.004783	3.726918	Up	phospholipase A2 group X	
UTF1	0.940212	0.000346	0.004796	3.725564	Up	undifferentiated embryonic cell transcription factor 1	
CELF4	0.871614	0.00035	0.004827	3.722421	Up	CUGBP Elav-like family member 4	
BTBD16	0.859346	0.000354	0.004868	3.71863	Up	BTB domain containing 16	
TMPRSS12	0.87545	0.000356	0.004868	3.717707	Up	transmembrane serine protease 12	
SPRY4	0.899144	0.000369	0.004997	3.707011	Up	sprouty RTK signaling antagonist 4	
FUT6	0.88066	0.000369	0.004997	3.706933	Up	fucosyltransferase 6	
LINC01551	0.87545	0.000372	0.005023	3.704451	Up	long intergenic non-protein coding RNA 1551	
GNG12	0.937701	0.000372	0.005023	3.704226	Up	G protein subunit gamma 12	
RD3	0.880776	0.000374	0.005038	3.703076	Up	retinal degeneration 3, GUCY2D regulator	
DKK1	0.950344	0.000383	0.005138	3.695639	Up	dickkopf WNT signaling pathway inhibitor 1	
NUTM2F	0.890116	0.000384	0.005147	3.694933	Up	NUT family member 2F	
NPAP1	0.841125	0.000385	0.00515	3.694524	Up	nuclear pore associated protein 1	
PDE10A	0.838498	0.000402	0.005283	3.681647	Up	phosphodiesterase 10A	
BDKRB2	0.859999	0.000401	0.005283	3.681948	Up	bradykinin receptor B2	
HES5	0.898473	0.000404	0.005292	3.680429	Up	hes family bHLH transcription factor 5	
SCEL	0.834584	0.000413	0.005347	3.673264	Up	sciellin	
TMIGD1	0.827595	0.000413	0.005347	3.673264	Up	transmembrane and immunoglobulin domain containing 1	
PDE1A	0.866557	0.000413	0.005347	3.673886	Up	phosphodiesterase 1A	
GOLGA8G	1.023375	0.000419	0.005389	3.669375	Up	golgin A8 family member G	
C12orf77	0.836295	0.000436	0.005501	3.657361	Up	chromosome 12 open reading frame 77	
PRSS12	0.917236	0.000437	0.005502	3.65691	Up	serine protease 12	
RBM46	0.951241	0.000438	0.005502	3.6564	Up	RNA binding motif protein 46	
KRT78	0.835349	0.000439	0.005502	3.655735	Up	keratin 78	
TAC4	0.866135	0.00044	0.005508	3.654937	Up	tachykinin precursor 4	
KRBOX1	0.865226	0.000439	0.005502	3.655612	Up	KRAB box domain containing 1	
PERM1	0.85926	0.000442	0.005508	3.6534	Up	PPARGC1 and ESRR induced regulator, muscle 1	
CAPN9	0.968118	0.000455	0.005612	3.644617	Up	calpain 9	
MAS1	0.874605	0.000469	0.005723	3.635607	Up	MAS1 proto-oncogene, G protein-coupled receptor	
ALDOB	0.840212	0.000481	0.005821	3.628603	Up	aldolase, fructose-bisphosphate B	
FAM131C	0.843355	0.000483	0.005829	3.627366	Up	family with sequence similarity 131 member C	
FAM71D	1.002051	0.000482	0.005825	3.628064	Up	family with sequence similarity 71 member D	
TMEM72	0.847662	0.000505	0.005976	3.613798	Up	transmembrane protein 72	
GSX2	0.884597	0.000505	0.005976	3.613792	Up	GS homeobox 2	
RASA4B	1.064804	0.000499	0.005946	3.617563	Up	RAS p21 protein activator 4B	
HSPB2	0.843999	0.000508	0.005984	3.611888	Up	heat shock protein family B (small) member 2	
FSTL5	0.877677	0.000523	0.006098	3.603482	Up	follistatin like 5	
RAX2	0.84501	0.000525	0.006112	3.60243	Up	retina and anterior neural fold homeobox 2	
BCO1	0.833952	0.000536	0.006202	3.59581	Up	beta-carotene oxygenase 1	
GPR62	0.86578	0.000536	0.006202	3.595965	Up	G protein-coupled receptor 62	
CDH18	0.829682	0.00054	0.006224	3.593699	Up	cadherin 18	
HSPE1-MOB4	0.902692	0.000545	0.006264	3.590982	Up	HSPE1-MOB4 readthrough	
MEGF10	0.828489	0.000545	0.006264	3.590973	Up	multiple EGF like domains 10	
RASSF9	0.833952	0.000582	0.006473	3.571637	Up	Ras association domain family member 9	
PALM2AKAP2	0.841084	0.000608	0.006683	3.558351	Up	PALM2 and AKAP2 fusion	
ZFPM2	0.83487	0.000614	0.00671	3.555258	Up	zinc finger protein, FOG family member 2	
IL21	0.872983	0.00063	0.006805	3.547506	Up	interleukin 21	
TCHH	0.913257	0.000641	0.006828	3.542218	Up	trichohyalin	
DNAI1	0.825247	0.000645	0.006844	3.540376	Up	dynein axonemal intermediate chain 1	
LHX2	0.825638	0.000664	0.006981	3.531404	Up	LIM homeobox 2	
TCIM	0.880336	0.000669	0.007015	3.529277	Up	transcriptional and immune response regulator	
CHKB-CPT1B	0.908843	0.000671	0.007024	3.52834	Up	CHKB-CPT1B readthrough (NMD candidate)	
FAXC	0.832545	0.000693	0.007167	3.518441	Up	failed axon connections homolog, metaxin like GST domain containing	
SPECC1L-ADORA2A	0.828726	0.000728	0.00737	3.503843	Up	SPECC1L-ADORA2A readthrough (NMD candidate)	
DPP10	0.834719	0.000736	0.007433	3.500365	Up	dipeptidyl peptidase like 10	
AMER2	0.854851	0.000749	0.00752	3.494939	Up	APC membrane recruitment protein 2	
SPATA22	1.255195	0.000761	0.007595	3.490127	Up	spermatogenesis associated 22	
TMEM151A	0.881109	0.000781	0.007713	3.482345	Up	transmembrane protein 151A	
ATP1A4	1.689421	0.000778	0.007691	3.483567	Up	ATPase Na+/K+ transporting subunit alpha 4	
HOXB7	0.989446	0.000781	0.007714	3.482117	Up	homeobox B7	
CCDC169	0.897611	0.000815	0.007946	3.469141	Up	coiled-coil domain containing 169	
SPINK4	0.843362	0.000821	0.007979	3.466965	Up	serine peptidase inhibitor, Kazal type 4	
LKAAEAR1	1.056911	0.000821	0.007979	3.467037	Up	LKAAEAR motif containing 1	
BPIFC	0.838473	0.000822	0.007982	3.466596	Up	BPI fold containing family C	
EIF4E1B	0.823457	0.000847	0.008124	3.457562	Up	eukaryotic translation initiation factor 4E family member 1B	
COX8C	0.823798	0.000853	0.008147	3.455114	Up	cytochrome c oxidase subunit 8C	
MIF	1.110365	0.000941	0.008696	3.425057	Up	macrophage migration inhibitory factor	
LRRC71	0.833583	0.000886	0.008347	3.443592	Up	leucine rich repeat containing 71	
EPB41L4B	1.108212	0.000896	0.008413	3.440042	Up	erythrocyte membrane protein band 4.1 like 4B	
MYRIP	0.847925	0.000903	0.008457	3.437595	Up	myosin VIIA and Rab interacting protein	
CORO7-PAM16	0.904171	0.000939	0.008696	3.425726	Up	CORO7-PAM16 readthrough	
ZSWIM2	0.880153	0.000983	0.008909	3.411475	Up	zinc finger SWIM-type containing 2	
TPPP2	0.85002	0.000983	0.008909	3.411351	Up	tubulin polymerization promoting protein family member 2	
PTPN20	0.978815	0.000982	0.008909	3.411957	Up	protein tyrosine phosphatase non-receptor type 20	
CST8	0.828046	0.000998	0.008992	3.40695	Up	cystatin 8	
KCNH5	0.840209	0.001002	0.009002	3.405458	Up	potassium voltage-gated channel subfamily H member 5	
WFDC1	0.88007	0.001062	0.009383	3.387406	Up	WAP four-disulfide core domain 1	
OR3A1	0.842916	0.001063	0.009383	3.387314	Up	olfactory receptor family 3 subfamily A member 1 (gene/pseudogene)	
EXOC3L4	0.847958	0.001109	0.009648	3.374087	Up	exocyst complex component 3 like 4	
GHSR	0.849126	0.001113	0.009673	3.372795	Up	growth hormone secretagogue receptor	
GDA	0.889672	0.001119	0.009709	3.371151	Up	guanine deaminase	
SHISA8	0.861981	0.001118	0.009704	3.371469	Up	shisa family member 8	
ZSCAN10	0.86053	0.001192	0.01015	3.351508	Up	zinc finger and SCAN domain containing 10	
GDPD4	0.825987	0.001228	0.010386	3.342107	Up	glycerophosphodiesterphosphodiesterase domain containing 4	
C4orf48	0.963154	0.001242	0.010457	3.338544	Up	chromosome 4 open reading frame 48	
SPDEF	0.881194	0.001231	0.010399	3.341393	Up	SAM pointed domain containing ETS transcription factor	
PMCH	0.938262	0.001273	0.010644	3.330774	Up	pro-melanin concentrating hormone	
TMEM121	1.161269	0.001281	0.010665	3.328878	Up	transmembrane protein 121	
ESRP2	0.86225	0.001323	0.010867	3.318668	Up	epithelial splicing regulatory protein 2	
GOLGA6L9	0.913468	0.001369	0.011101	3.30796	Up	golgin A6 family like 9	
LRRTM2	0.922665	0.001414	0.011348	3.297763	Up	leucine rich repeat transmembrane neuronal 2	
RFX4	0.831273	0.001445	0.011513	3.290825	Up	regulatory factor X4	
FAP	0.99642	0.001437	0.011476	3.292583	Up	fibroblast activation protein alpha	
CTAGE6	0.885238	0.001484	0.011748	3.282458	Up	CTAGE family member 6	
GPC6	0.897005	0.001603	0.012397	3.257934	Up	glypican 6	
BOLA2B	1.348547	0.001587	0.012335	3.261001	Up	bolA family member 2B	
ANGPT4	0.829222	0.001678	0.012731	3.24332	Up	angiopoietin 4	
GLYATL1	0.859167	0.001744	0.013066	3.230845	Up	glycine-N-acyltransferase like 1	
ALPI	0.83854	0.001782	0.013284	3.223964	Up	alkaline phosphatase, intestinal	
LINC02312	0.846436	0.00182	0.013468	3.217256	Up	long intergenic non-protein coding RNA 2312	
MAGI1	0.834329	0.001979	0.014332	3.190204	Up	membrane associated guanylate kinase, WW and PDZ domain containing 1	
LOC105377590	0.827061	0.002067	0.014767	3.176133	Up	uncharacterized LOC105377590	
UGT2A3	1.084825	0.002072	0.014775	3.175415	Up	UDP glucuronosyltransferase family 2 member A3	
SEMA6B	0.97473	0.002148	0.015121	3.163707	Up	semaphorin 6B	
RFPL4A	1.618683	0.002173	0.015241	3.159916	Up	ret finger protein like 4A	
P2RX2	0.823852	0.002237	0.015538	3.150517	Up	purinergic receptor P2X 2	
COL1A2	0.873234	0.002262	0.015671	3.14683	Up	collagen type I alpha 2 chain	
CDKL2	0.900439	0.002301	0.015857	3.141203	Up	cyclin dependent kinase like 2	
CLUL1	0.934087	0.002373	0.016257	3.131195	Up	clusterin like 1	
MIPOL1	0.862616	0.002732	0.017911	3.084864	Up	mirror-image polydactyly 1	
PKNOX2	0.859428	0.002743	0.017952	3.083472	Up	PBX/knotted 1 homeobox 2	
TRHDE	0.950234	0.002983	0.019018	3.05568	Up	thyrotropin releasing hormone degrading enzyme	
BCAR1	0.894146	0.002995	0.019072	3.054351	Up	BCAR1 scaffold protein, Cas family member	
ZPLD1	0.83027	0.003108	0.019559	3.042048	Up	zonapellucida like domain containing 1	
ECM2	0.881103	0.003166	0.019806	3.035863	Up	extracellular matrix protein 2	
C8B	0.83507	0.003179	0.019849	3.034511	Up	complement C8 beta chain	
ZSCAN4	0.824589	0.003335	0.020502	3.018496	Up	zinc finger and SCAN domain containing 4	
DUSP15	0.835463	0.003583	0.021602	2.994384	Up	dual specificity phosphatase 15	
FSIP2	1.051501	0.003654	0.021884	2.987718	Up	fibrous sheath interacting protein 2	
GYPA	0.838823	0.003832	0.022535	2.971603	Up	glycophorin A (MNS blood group)	
MICU3	0.836643	0.004058	0.023381	2.952197	Up	mitochondrial calcium uptake family member 3	
ABCB5	0.828852	0.00415	0.023751	2.944524	Up	ATP binding cassette subfamily B member 5	
MTRNR2L1	2.018372	0.004535	0.025279	2.914228	Up	MT-RNR2 like 1	
TRAPPC5	0.871477	0.004667	0.025812	2.904327	Up	trafficking protein particle complex 5	
MYT1L	1.20911	0.004898	0.026721	2.88766	Up	myelin transcription factor 1 like	
DKK2	0.89525	0.005299	0.028207	2.860463	Up	dickkopf WNT signaling pathway inhibitor 2	
SRXN1	1.018618	0.005734	0.029851	2.832953	Up	sulfiredoxin 1	
HBZ	1.056697	0.005869	0.030365	2.824809	Up	hemoglobin subunit zeta	
GABRB3	0.916767	0.005973	0.030651	2.818616	Up	gamma-aminobutyric acid type A receptor beta3 subunit	
TDRD15	0.860035	0.006628	0.033041	2.781952	Up	tudor domain containing 15	
PDF	0.908183	0.00747	0.036155	2.739388	Up	peptide deformylase, mitochondrial	
CCDC144A	1.387575	0.007676	0.036885	2.729663	Up	coiled-coil domain containing 144A	
MRPL12	1.043343	0.011635	0.049872	2.57764	Up	mitochondrial ribosomal protein L12	
SIRPB2	−0.47534	8.44E-09	4.63E-05	−6.37763	Down	signal regulatory protein beta 2	
RNF122	−0.40229	1.36E-08	4.63E-05	−6.26932	Down	ring finger protein 122	
SYK	−0.32942	2.38E-08	6.66E-05	−6.1427	Down	spleen associated tyrosine kinase	
LASP1	−0.39444	7.15E-08	0.000109	−5.89021	Down	LIM and SH3 protein 1	
SLC44A2	−0.3592	1.09E-07	0.000141	−5.79236	Down	solute carrier family 44 member 2	
C5AR2	−0.45692	1.58E-07	0.000165	−5.70624	Down	complement component 5a receptor 2	
PRKACA	−0.49178	2.12E-07	0.000197	−5.63649	Down	protein kinase cAMP-activated catalytic subunit alpha	
TP53INP2	−0.59653	2.35E-07	0.000198	−5.61183	Down	tumor protein p53 inducible nuclear protein 2	
F11R	−0.37662	3.27E-07	0.000198	−5.53422	Down	F11 receptor	
INKA2	−0.4438	3.25E-07	0.000198	−5.5354	Down	inka box actin regulator 2	
GABARAP	−0.49797	3.53E-07	0.000205	−5.5156	Down	GABA type A receptor-associated protein	
CRTC2	−0.48092	3.66E-07	0.000205	−5.50724	Down	CREB regulated transcription coactivator 2	
RXRB	−0.3761	4.52E-07	0.00023	−5.45685	Down	retinoid X receptor beta	
CASP9	−0.27139	5.84E-07	0.000258	−5.39557	Down	caspase 9	
C6orf136	−0.3863	7.96E-07	0.000297	−5.321	Down	chromosome 6 open reading frame 136	
CTDSP2	−0.32959	7.48E-07	0.000288	−5.3362	Down	CTD small phosphatase 2	
DPEP2	−0.41998	7.56E-07	0.000288	−5.33349	Down	dipeptidase 2	
TMEM234	−0.38446	8.95E-07	0.000313	−5.29278	Down	transmembrane protein 234	
TMEM179B	−0.27682	9.14E-07	0.000313	−5.28769	Down	transmembrane protein 179B	
SNX11	−0.32558	9.47E-07	0.000318	−5.27902	Down	sorting nexin 11	
AGAP9	−1.41788	4.42E-07	0.00023	−5.46256	Down	ArfGAP with GTPase domain, ankyrin repeat and PH domain 9	
FAM219A	−0.45019	1.17E-06	0.000376	−5.22858	Down	family with sequence similarity 219 member A	
CSNK2B	−0.29639	1.41E-06	0.00043	−5.18303	Down	casein kinase 2 beta	
TMEM127	−0.34378	1.43E-06	0.00043	−5.1782	Down	transmembrane protein 127	
MEFV	−0.52752	1.62E-06	0.00046	−5.14816	Down	MEFV innate immuity regulator, pyrin	
KCTD21	−0.31051	1.92E-06	0.000504	−5.1065	Down	potassium channel tetramerization domain containing 21	
TMBIM1	−0.32832	1.86E-06	0.0005	−5.11415	Down	transmembrane BAX inhibitor motif containing 1	
IL17RA	−0.4439	1.99E-06	0.000507	−5.09788	Down	interleukin 17 receptor A	
RAB37	−0.31838	2.04E-06	0.00051	−5.09207	Down	RAB37, member RAS oncogene family	
GAB2	−0.44824	2.15E-06	0.000532	−5.07836	Down	GRB2 associated binding protein 2	
BLOC1S3	−0.28757	2.43E-06	0.000544	−5.04845	Down	biogenesis of lysosomal organelles complex 1 subunit 3	
APOL2	−0.46372	2.38E-06	0.000544	−5.05359	Down	apolipoprotein L2	
KIAA0319L	−0.35586	2.4E-06	0.000544	−5.05141	Down	KIAA0319 like	
SIPA1L1	−0.37533	2.84E-06	0.000592	−5.00972	Down	signal induced proliferation associated 1 like 1	
ARF3	−0.39406	2.86E-06	0.000592	−5.00779	Down	ADP ribosylation factor 3	
CASC3	−0.28443	2.89E-06	0.000592	−5.00572	Down	CASC3 exon junction complex subunit	
ARHGAP30	−0.2905	3.04E-06	0.000593	−4.99258	Down	Rho GTPase activating protein 30	
TOP3A	−0.35048	3.21E-06	0.000593	−4.97967	Down	DNA topoisomerase III alpha	
C16orf54	−0.26747	3.24E-06	0.000593	−4.97743	Down	chromosome 16 open reading frame 54	
BEST1	−0.50351	3.37E-06	0.000593	−4.96756	Down	bestrophin 1	
C6orf89	−0.28041	3.39E-06	0.000593	−4.96553	Down	chromosome 6 open reading frame 89	
SUSD6	−0.37933	3.37E-06	0.000593	−4.96707	Down	sushi domain containing 6	
STAT6	−0.386	3.46E-06	0.000594	−4.96042	Down	signal transducer and activator of transcription 6	
SH3D21	−0.62962	4.05E-06	0.000648	−4.92115	Down	SH3 domain containing 21	
FAM214B	−0.36064	3.72E-06	0.000615	−4.94269	Down	family with sequence similarity 214 member B	
AGPAT1	−0.37351	3.97E-06	0.000641	−4.92639	Down	1-acylglycerol-3-phosphate O-acyltransferase 1	
CPSF7	−0.33791	4.39E-06	0.00067	−4.90105	Down	cleavage and polyadenylation specific factor 7	
EGLN2	−0.33487	4.97E-06	0.00072	−4.86981	Down	egl-9 family hypoxia inducible factor 2	
VAMP2	−0.32282	5.23E-06	0.000738	−4.85729	Down	vesicle associated membrane protein 2	
ARHGAP1	−0.46187	5.32E-06	0.000738	−4.85289	Down	Rho GTPase activating protein 1	
CPT2	−0.30399	6.25E-06	0.00082	−4.81205	Down	carnitinepalmitoyltransferase 2	
OSCAR	−0.4824	7.26E-06	0.000909	−4.77404	Down	osteoclast associated Ig-like receptor	
EXTL3	−0.44258	7.37E-06	0.00091	−4.77025	Down	exostosin like glycosyltransferase 3	
NOMO2	−0.58975	8.27E-06	0.000925	−4.74093	Down	NODAL modulator 2	
PIK3R5	−0.28302	8.01E-06	0.000925	−4.74887	Down	phosphoinositide-3-kinase regulatory subunit 5	
IGF2R	−0.52404	8.21E-06	0.000925	−4.74277	Down	insulin like growth factor 2 receptor	
EPHX1	−0.55423	9.97E-06	0.001014	−4.6931	Down	epoxide hydrolase 1	
MAPKAPK2	−0.29705	8.55E-06	0.000925	−4.73251	Down	MAPK activated protein kinase 2	
STAT3	−0.36483	9.1E-06	0.000967	−4.71639	Down	signal transducer and activator of transcription 3	
NPIPB3	−0.96437	1.13E-05	0.001075	−4.66109	Down	nuclear pore complex interacting protein family member B3	
RNF19B	−0.29623	1.04E-05	0.001038	−4.681	Down	ring finger protein 19B	
PBX2	−0.36625	1.07E-05	0.001047	−4.67424	Down	PBX homeobox 2	
MTMR3	−0.30993	1.16E-05	0.001078	−4.65496	Down	myotubularin related protein 3	
PI4KB	−0.32556	1.17E-05	0.001078	−4.65218	Down	phosphatidylinositol 4-kinase beta	
GLYR1	−0.33045	1.23E-05	0.001111	−4.63863	Down	glyoxylatereductase 1 homolog	
TK2	−0.30672	1.33E-05	0.001135	−4.61837	Down	thymidine kinase 2	
RAF1	−0.26209	1.34E-05	0.001139	−4.61618	Down	Raf-1 proto-oncogene, serine/threonine kinase	
PPP1R10	−0.32024	1.44E-05	0.001195	−4.59736	Down	protein phosphatase 1 regulatory subunit 10	
ARRB2	−0.26361	1.45E-05	0.001195	−4.59727	Down	arrestin beta 2	
ATP6V0D1	−0.39381	1.45E-05	0.001196	−4.59576	Down	ATPase H+ transporting V0 subunit d1	
SMAP2	−0.35835	1.47E-05	0.001201	−4.59347	Down	small ArfGAP2	
KDELR1	−0.34331	1.53E-05	0.001232	−4.58193	Down	KDEL endoplasmic reticulum protein retention receptor 1	
ATP6V0A1	−0.3471	1.64E-05	0.00127	−4.5643	Down	ATPase H+ transporting V0 subunit a1	
NDST1	−0.51316	1.68E-05	0.001284	−4.55772	Down	N-deacetylase and N-sulfotransferase 1	
SF3A1	−0.36624	1.79E-05	0.001319	−4.54121	Down	splicing factor 3a subunit 1	
TPCN2	−0.41036	1.93E-05	0.001352	−4.52153	Down	two pore segment channel 2	
TAPBP	−0.35224	1.89E-05	0.001335	−4.52755	Down	TAP binding protein	
TRPC4AP	−0.27784	1.95E-05	0.001354	−4.51955	Down	transient receptor potential cation channel subfamily C member 4 associated protein	
NDE1	−0.3477	1.98E-05	0.001369	−4.51459	Down	nudE neurodevelopment protein 1	
PIGS	−0.26132	2.15E-05	0.001407	−4.49392	Down	phosphatidylinositol glycan anchor biosynthesis class S	
TIFAB	−0.67622	2.29E-05	0.001441	−4.47698	Down	TIFA inhibitor	
DGKG	−0.35931	2.61E-05	0.001504	−4.44284	Down	diacylglycerol kinase gamma	
CYTH2	−0.27	2.65E-05	0.001515	−4.43896	Down	cytohesin 2	
PDZD3	−1.1876	2.23E-05	0.001411	−4.48422	Down	PDZ domain containing 3	
PXN	−0.40274	2.8E-05	0.00157	−4.42421	Down	paxillin	
MOB3A	−0.38776	2.99E-05	0.001621	−4.40616	Down	MOB kinase activator 3A	
CHKB	−0.69168	3.78E-05	0.001757	−4.34418	Down	choline kinase beta	
TMEM121B	−0.58616	3.6E-05	0.001757	−4.35738	Down	transmembrane protein 121B	
PEAK3	−0.64308	3.86E-05	0.001766	−4.33852	Down	PEAK family member 3	
APOL1	−0.43281	3.39E-05	0.001712	−4.3734	Down	apolipoprotein L1	
IRF1	−0.27784	3.51E-05	0.001738	−4.36395	Down	interferon regulatory factor 1	
MINDY1	−0.37272	3.76E-05	0.001757	−4.34574	Down	MINDY lysine 48 deubiquitinase 1	
RNF24	−0.38862	3.8E-05	0.001759	−4.34233	Down	ring finger protein 24	
PRCC	−0.26648	4.35E-05	0.001845	−4.30641	Down	proline rich mitotic checkpoint control factor	
BORCS8	−0.36271	4.62E-05	0.001879	−4.28997	Down	BLOC-1 related complex subunit 8	
ITGA5	−0.35353	4.34E-05	0.001845	−4.30671	Down	integrin subunit alpha 5	
SMPD2	−0.28647	5.33E-05	0.001995	−4.25178	Down	sphingomyelinphosphodiesterase 2	
SMARCD1	−0.33626	5.11E-05	0.001972	−4.26291	Down	SWI/SNF related, matrix associated, actin dependent regulator of chromatin, subfamily d, member 1	
SIK3	−0.26974	5.36E-05	0.002002	−4.25023	Down	SIK family kinase 3	
C9orf129	−1.18267	5.01E-05	0.001962	−4.26853	Down	chromosome 9 open reading frame 129	
SCAMP5	−0.60055	7.19E-05	0.00225	−4.17021	Down	secretory carrier membrane protein 5	
IP6K1	−0.32228	5.67E-05	0.002052	−4.23471	Down	inositol hexakisphosphate kinase 1	
TRANK1	−0.42013	5.8E-05	0.002073	−4.22853	Down	tetratricopeptide repeat and ankyrin repeat containing 1	
PRR14L	−0.29747	6.19E-05	0.002108	−4.21102	Down	proline rich 14 like	
SETDB1	−0.28227	6.25E-05	0.002114	−4.20826	Down	SET domain bifurcated histone lysine methyltransferase 1	
ZNFX1	−0.32602	6.25E-05	0.002114	−4.20844	Down	zinc finger NFX1-type containing 1	
GSK3A	−0.35215	6.47E-05	0.002126	−4.19898	Down	glycogen synthase kinase 3 alpha	
GPSM3	−0.38027	6.28E-05	0.002114	−4.20685	Down	G protein signaling modulator 3	
CLN3	−0.31966	7.41E-05	0.002275	−4.16164	Down	CLN3 lysosomal/endosomaltransmembrane protein, battenin	
CYB561D1	−0.30277	7.06E-05	0.002224	−4.17511	Down	cytochrome b561 family member D1	
GBA2	−0.26836	7.06E-05	0.002224	−4.17523	Down	glucosylceramidase beta 2	
SEC14L1	−0.31028	7.06E-05	0.002224	−4.17493	Down	SEC14 like lipid binding 1	
KCTD2	−0.28549	7.52E-05	0.002293	−4.15781	Down	potassium channel tetramerization domain containing 2	
RGL3	−0.75662	8.74E-05	0.002495	−4.11629	Down	ral guanine nucleotide dissociation stimulator like 3	
DTX4	−0.40937	9.03E-05	0.002513	−4.1075	Down	deltex E3 ubiquitin ligase 4	
CLEC4C	−0.88455	0.000102	0.002653	−4.07383	Down	C-type lectin domain family 4 member C	
GTPBP1	−0.41546	8.09E-05	0.002396	−4.13758	Down	GTP binding protein 1	
RNF135	−0.27712	8.49E-05	0.002457	−4.12445	Down	ring finger protein 135	
ASF1B	−0.45437	9.24E-05	0.002521	−4.10114	Down	anti-silencing function 1B histone chaperone	
ARAP3	−0.52053	8.66E-05	0.002477	−4.11882	Down	ArfGAP with RhoGAP domain, ankyrin repeat and PH domain 3	
ST6GALNAC2	−0.38956	9.6E-05	0.002588	−4.09059	Down	ST6 N-acetylgalactosaminide alpha-2,6-sialyltransferase 2	
VPS37C	−0.3927	0.000106	0.002705	−4.0635	Down	VPS37C subunit of ESCRT-I	
TNFRSF1B	−0.32737	9.6E-05	0.002588	−4.09033	Down	TNF receptor superfamily member 1B	
LY6G5B	−0.41293	0.000115	0.002866	−4.03965	Down	lymphocyte antigen 6 family member G5B	
PTAFR	−0.29634	9.8E-05	0.002614	−4.08471	Down	platelet activating factor receptor	
MYADM	−0.41938	0.000102	0.00266	−4.07268	Down	myeloid associated differentiation marker	
MBD6	−0.55341	0.000105	0.002693	−4.06584	Down	methyl-CpG binding domain protein 6	
RETREG2	−0.2614	0.000107	0.002723	−4.06108	Down	reticulophagy regulator family member 2	
LMBR1L	−0.27058	0.000114	0.002843	−4.04291	Down	limb development membrane protein 1 like	
BET1L	−0.32294	0.000118	0.002909	−4.03319	Down	Bet1 golgi vesicular membrane trafficking protein like	
SORT1	−0.34943	0.000117	0.002904	−4.03473	Down	sortilin 1	
MTF1	−0.29397	0.000123	0.002957	−4.02199	Down	metal regulatory transcription factor 1	
C19orf54	−0.34173	0.000147	0.003138	−3.97225	Down	chromosome 19 open reading frame 54	
CXCL16	−0.35275	0.000124	0.002964	−4.01814	Down	C-X-C motif chemokine ligand 16	
RUBCN	−0.3292	0.000126	0.002975	−4.01499	Down	rubicon autophagy regulator	
SNX27	−0.26986	0.000124	0.002964	−4.01952	Down	sorting nexin 27	
RAB11FIP1	−0.29163	0.000123	0.002964	−4.02057	Down	RAB11 family interacting protein 1	
LGALS9	−0.3923	0.000126	0.002975	−4.01527	Down	galectin 9	
PTPN18	−0.27467	0.000129	0.003	−4.00738	Down	protein tyrosine phosphatase non-receptor type 18	
SIRPA	−0.2873	0.00013	0.003	−4.00656	Down	signal regulatory protein alpha	
CHST15	−0.27829	0.000133	0.003031	−3.99914	Down	carbohydrate sulfotransferase 15	
UBN1	−0.38058	0.000134	0.003031	−3.99765	Down	ubinuclein 1	
NCOA6	−0.32966	0.000148	0.003154	−3.9687	Down	nuclear receptor coactivator 6	
ARSG	−0.27821	0.00017	0.003316	−3.92992	Down	arylsulfatase G	
EIF3CL	−1.35741	0.0002	0.003608	−3.88399	Down	eukaryotic translation initiation factor 3 subunit C like	
POLR2A	−0.61547	0.000153	0.003182	−3.96085	Down	RNA polymerase II subunit A	
AOC2	−0.45479	0.000182	0.003432	−3.91142	Down	amine oxidase copper containing 2	
NLRP12	−0.34542	0.00016	0.003242	−3.94814	Down	NLR family pyrin domain containing 12	
SEC24C	−0.30954	0.000158	0.003221	−3.95154	Down	SEC24 homolog C, COPII coat complex component	
KCNMB1	−0.40521	0.000212	0.003705	−3.86745	Down	potassium calcium-activated channel subfamily M regulatory beta subunit 1	
SLC48A1	−0.36957	0.000185	0.003451	−3.90712	Down	solute carrier family 48 member 1	
ZNF592	−0.30397	0.00016	0.003247	−3.94689	Down	zinc finger protein 592	
MEF2D	−0.35897	0.000165	0.003278	−3.93885	Down	myocyte enhancer factor 2D	
SLC25A44	−0.26274	0.000166	0.003282	−3.93716	Down	solute carrier family 25 member 44	
PPCDC	−0.31808	0.000179	0.003395	−3.91543	Down	phosphopantothenoylcysteine decarboxylase	
PLEKHO2	−0.35445	0.000175	0.003346	−3.92211	Down	pleckstrin homology domain containing O2	
PIGO	−0.35859	0.000198	0.003586	−3.88709	Down	phosphatidylinositol glycan anchor biosynthesis class O	
ZBTB22	−0.28471	0.000214	0.00371	−3.86516	Down	zinc finger and BTB domain containing 22	
ZSWIM1	−0.29063	0.000229	0.003835	−3.84574	Down	zinc finger SWIM-type containing 1	
DUSP18	−0.46032	0.000234	0.003882	−3.83938	Down	dual specificity phosphatase 18	
BAZ2A	−0.41201	0.000191	0.003523	−3.89726	Down	bromodomain adjacent to zinc finger domain 2A	
ARRB1	−0.2686	0.000196	0.003567	−3.89016	Down	arrestin beta 1	
SMG5	−0.37995	0.0002	0.003608	−3.88407	Down	SMG5 nonsense mediated mRNA decay factor	
DAP	−0.26058	0.000204	0.003634	−3.87917	Down	death associated protein	
RIMS3	−0.50216	0.000274	0.004209	−3.79409	Down	regulating synaptic membrane exocytosis 3	
WIPF2	−0.31686	0.000214	0.00371	−3.86514	Down	WAS/WASL interacting protein family member 2	
NSD1	−0.3405	0.000219	0.003768	−3.85773	Down	nuclear receptor binding SET domain protein 1	
VPS53	−0.37707	0.00024	0.003926	−3.83213	Down	VPS53 subunit of GARP complex	
RASGRP4	−0.33615	0.000225	0.00382	−3.85013	Down	RAS guanyl releasing protein 4	
FAM168A	−0.26973	0.000246	0.003969	−3.82519	Down	family with sequence similarity 168 member A	
ZBTB3	−0.43164	0.000328	0.004639	−3.74095	Down	zinc finger and BTB domain containing 3	
PHC2	−0.37839	0.000246	0.003969	−3.82531	Down	polyhomeotic homolog 2	
RGL2	−0.40321	0.000252	0.004016	−3.81807	Down	ral guanine nucleotide dissociation stimulator like 2	
SPINT1	−0.42854	0.00029	0.004307	−3.77766	Down	serine peptidase inhibitor, Kunitz type 1	
CBX7	−0.28076	0.000267	0.004169	−3.80078	Down	chromobox 7	
ADAM19	−0.30366	0.000284	0.004279	−3.78351	Down	ADAM metallopeptidase domain 19	
PRR16	−0.99695	0.000343	0.004771	−3.72824	Down	proline rich 16	
SP110	−0.27632	0.00029	0.004307	−3.77759	Down	SP110 nuclear body protein	
SIGLEC14	−0.5177	0.000302	0.004436	−3.76517	Down	sialic acid binding Ig like lectin 14	
RNPEP	−0.35393	0.000304	0.004441	−3.76372	Down	arginylaminopeptidase	
ABAT	−0.30955	0.000326	0.004625	−3.74264	Down	4-aminobutyrate aminotransferase	
ITGAX	−0.51435	0.000313	0.004529	−3.75521	Down	integrin subunit alpha X	
ARHGEF11	−0.37753	0.000329	0.00464	−3.73998	Down	Rho guanine nucleotide exchange factor 11	
SLC35F6	−0.29772	0.000368	0.004997	−3.70727	Down	solute carrier family 35 member F6	
ACP2	−0.30411	0.00039	0.005185	−3.69061	Down	acid phosphatase 2, lysosomal	
SYVN1	−0.3935	0.000362	0.004927	−3.71249	Down	synoviolin 1	
TMEM229B	−0.33641	0.000404	0.005292	−3.68022	Down	transmembrane protein 229B	
C7orf26	−0.29812	0.000413	0.005347	−3.67329	Down	chromosome 7 open reading frame 26	
ALKBH6	−0.40327	0.000526	0.006123	−3.6017	Down	alkB homolog 6	
MAST3	−0.42877	0.000386	0.005167	−3.69332	Down	microtubule associated serine/threonine kinase 3	
ADAR	−0.28407	0.000388	0.005173	−3.69224	Down	adenosine deaminase RNA specific	
MAP3K3	−0.26552	0.000395	0.005231	−3.68687	Down	mitogen-activated protein kinase kinasekinase 3	
MFSD14C	−0.51982	0.000578	0.006457	−3.57334	Down	major facilitator superfamily domain containing 14C	
PVR	−0.35231	0.000528	0.006129	−3.6006	Down	PVR cell adhesion molecule	
STIM1	−0.32389	0.000409	0.005319	−3.67666	Down	stromal interaction molecule 1	
STK35	−0.26227	0.000425	0.005423	−3.66486	Down	serine/threonine kinase 35	
PILRA	−0.29576	0.000415	0.005359	−3.67194	Down	paired immunoglobin like type 2 receptor alpha	
PRKCD	−0.27159	0.000419	0.005389	−3.66952	Down	protein kinase C delta	
PFKFB4	−0.29916	0.00043	0.005461	−3.6616	Down	6-phosphofructo-2-kinase/fructose-2,6-biphosphatase 4	
RAB3D	−0.28769	0.000427	0.005423	−3.6641	Down	RAB3D, member RAS oncogene family	
NAGK	−0.26431	0.000445	0.005535	−3.65121	Down	N-acetylglucosamine kinase	
RELL1	−0.38387	0.000562	0.006383	−3.58164	Down	RELT like 1	
LCNL1	−1.04762	0.000615	0.006715	−3.55463	Down	lipocalin like 1	
ASPRV1	−0.51579	0.000567	0.006394	−3.57928	Down	aspartic peptidase retroviral like 1	
TBC1D13	−0.31201	0.000496	0.005933	−3.61904	Down	TBC1 domain family member 13	
CANT1	−0.26414	0.000469	0.005723	−3.63568	Down	calcium activated nucleotidase 1	
GPR107	−0.30509	0.000479	0.005804	−3.62991	Down	G protein-coupled receptor 107	
ATXN1L	−0.28945	0.000492	0.0059	−3.62183	Down	ataxin 1 like	
CHST14	−0.31667	0.000614	0.00671	−3.55538	Down	carbohydrate sulfotransferase 14	
SIGLEC9	−0.26149	0.0005	0.005946	−3.61695	Down	sialic acid binding Ig like lectin 9	
FLCN	−0.3041	0.000527	0.006129	−3.60096	Down	folliculin	
WRAP53	−0.28624	0.000632	0.006817	−3.54662	Down	WD repeat containing antisense to TP53	
RIPK3	−0.28326	0.000527	0.006129	−3.60102	Down	receptor interacting serine/threonine kinase 3	
FAM53C	−0.26774	0.000505	0.005976	−3.61408	Down	family with sequence similarity 53 member C	
WDFY4	−0.42866	0.000513	0.006018	−3.60918	Down	WDFY family member 4	
GALNT6	−0.30319	0.000593	0.006558	−3.56589	Down	polypeptide N-acetylgalactosaminyltransferase 6	
IQCN	−0.59607	0.000676	0.00706	−3.52611	Down	IQ motif containing N	
KCNIP2	−0.4831	0.00077	0.007639	−3.48639	Down	potassium voltage-gated channel interacting protein 2	
USP22	−0.28086	0.000539	0.006218	−3.59461	Down	ubiquitin specific peptidase 22	
APOM	−0.36363	0.0008	0.007857	−3.47507	Down	apolipoprotein M	
MAFF	−0.33734	0.000758	0.007573	−3.49137	Down	MAF bZIP transcription factor F	
TNFAIP2	−0.43598	0.000547	0.006267	−3.58999	Down	TNF alpha induced protein 2	
CRISPLD2	−0.44839	0.000554	0.006323	−3.58616	Down	cysteine rich secretory protein LCCL domain containing 2	
SMURF1	−0.28252	0.000613	0.006707	−3.55597	Down	SMAD specific E3 ubiquitin protein ligase 1	
SLC35E2A	−0.65437	0.000832	0.008038	−3.46296	Down	solute carrier family 35 member E2A	
ADAMTSL4-AS1	−0.62083	0.00084	0.008094	−3.4601	Down	ADAMTSL4 antisense RNA 1	
C17orf49	−0.35504	0.000843	0.008114	−3.45892	Down	chromosome 17 open reading frame 49	
MAPK13	−0.34929	0.00064	0.006828	−3.54262	Down	mitogen-activated protein kinase 13	
MAPRE3	−0.41136	0.000774	0.007663	−3.48489	Down	microtubule associated protein RP/EB family member 3	
RIPOR1	−0.45424	0.000623	0.00676	−3.55065	Down	RHO family interacting cell polarization regulator 1	
EPS15L1	−0.26529	0.000638	0.006823	−3.54359	Down	epidermal growth factor receptor pathway substrate 15 like 1	
RERE	−0.31584	0.000638	0.006823	−3.5437	Down	arginine-glutamic acid dipeptide repeats	
DGLUCY	−0.2802	0.000649	0.006868	−3.53869	Down	D-glutamate cyclase	
CSF3R	−0.37028	0.000656	0.006907	−3.53531	Down	colony stimulating factor 3 receptor	
HCG27	−0.41468	0.000665	0.006981	−3.53133	Down	HLA complex group 27	
GNAI2	−0.31016	0.000678	0.00706	−3.52531	Down	G protein subunit alpha i2	
HSH2D	−0.28176	0.000684	0.00711	−3.52276	Down	hematopoietic SH2 domain containing	
KIAA0513	−0.37074	0.000709	0.007246	−3.5118	Down	KIAA0513	
SEMA4A	−0.32944	0.000725	0.007356	−3.50486	Down	semaphorin 4A	
PLBD2	−0.42359	0.000941	0.008696	−3.42516	Down	phospholipase B domain containing 2	
LRP10	−0.35142	0.000757	0.007571	−3.49174	Down	LDL receptor related protein 10	
GATAD2B	−0.29403	0.000784	0.007727	−3.48124	Down	GATA zinc finger domain containing 2B	
NICN1	−0.31258	0.000918	0.008552	−3.43258	Down	nicolin 1	
DIAPH1	−0.31756	0.000808	0.007898	−3.47192	Down	diaphanous related formin 1	
ITGAL	−0.35006	0.000857	0.008169	−3.45376	Down	integrin subunit alpha L	
ZNF784	−0.32453	0.001326	0.010881	−3.31793	Down	zinc finger protein 784	
ARHGAP9	−0.32141	0.000888	0.00836	−3.44285	Down	Rho GTPase activating protein 9	
WWP2	−0.3584	0.000901	0.008438	−3.43845	Down	WW domain containing E3 ubiquitin protein ligase 2	
MICALL1	−0.44326	0.001108	0.009644	−3.37439	Down	MICAL like 1	
TP53	−0.28135	0.000961	0.008834	−3.4185	Down	tumor protein p53	
WDTC1	−0.41259	0.000934	0.008664	−3.4274	Down	WD and tetratricopeptide repeats 1	
UBE2C	−0.37342	0.001447	0.011521	−3.29045	Down	ubiquitin conjugating enzyme E2 C	
ORAI2	−0.26578	0.000971	0.008867	−3.41533	Down	ORAI calcium release-activated calcium modulator 2	
TMEM63C	−0.66345	0.001471	0.011659	−3.28516	Down	transmembrane protein 63C	
NAPA	−0.30533	0.00099	0.008953	−3.40941	Down	NSF attachment protein alpha	
SCAMP2	−0.27934	0.000995	0.008979	−3.40775	Down	secretory carrier membrane protein 2	
PSAP	−0.26071	0.001008	0.009037	−3.40361	Down	prosaposin	
ZNF385A	−0.44893	0.001055	0.009343	−3.38947	Down	zinc finger protein 385A	
XPO6	−0.28059	0.001023	0.009125	−3.3991	Down	exportin 6	
TREML2	−0.29456	0.001038	0.009222	−3.39466	Down	triggering receptor expressed on myeloid cells like 2	
TMEM106A	−0.35251	0.001317	0.010844	−3.32008	Down	transmembrane protein 106A	
CNNM4	−0.30466	0.001368	0.011101	−3.30823	Down	cyclin and CBS domain divalent metal cation transport mediator 4	
ADA2	−0.32201	0.001086	0.009543	−3.38059	Down	adenosine deaminase 2	
MPEG1	−0.26839	0.001092	0.009571	−3.37885	Down	macrophage expressed 1	
SLC9A1	−0.40045	0.001164	0.009954	−3.3589	Down	solute carrier family 9 member A1	
INTS3	−0.34455	0.001156	0.00991	−3.36104	Down	integrator complex subunit 3	
APOBEC3D	−0.30439	0.001328	0.010888	−3.31758	Down	apolipoprotein B mRNA editing enzyme catalytic subunit 3D	
ZNF324	−0.29262	0.001606	0.012416	−3.2573	Down	zinc finger protein 324	
SIDT2	−0.35226	0.001217	0.010314	−3.34508	Down	SID1 transmembrane family member 2	
TMEM214	−0.29447	0.001256	0.010538	−3.33517	Down	transmembrane protein 214	
ZNF687	−0.33153	0.001277	0.010659	−3.32985	Down	zinc finger protein 687	
MFN2	−0.28625	0.001236	0.01042	−3.34017	Down	mitofusin 2	
RAB36	−0.43526	0.001925	0.014054	−3.19916	Down	RAB36, member RAS oncogene family	
PLPPR2	−0.40458	0.001273	0.010644	−3.33082	Down	phospholipid phosphatase related 2	
DYSF	−0.45611	0.00128	0.010665	−3.32913	Down	dysferlin	
FIZ1	−0.28749	0.001808	0.013394	−3.21933	Down	FLT3 interacting zinc finger 1	
STK10	−0.26283	0.001306	0.010781	−3.3227	Down	serine/threonine kinase 10	
MAML3	−0.28886	0.001556	0.012181	−3.26737	Down	mastermind like transcriptional coactivator 3	
KSR1	−0.37381	0.001394	0.011257	−3.30217	Down	kinase suppressor of ras 1	
TRIM62	−0.30433	0.001646	0.012623	−3.2494	Down	tripartite motif containing 62	
SESN2	−0.3122	0.00169	0.012795	−3.24092	Down	sestrin 2	
MARK2	−0.31865	0.00141	0.011332	−3.29873	Down	microtubule affinity regulating kinase 2	
CDIP1	−0.31975	0.00157	0.012243	−3.26456	Down	cell death inducing p53 target 1	
PAQR6	−0.61509	0.002215	0.015442	−3.15361	Down	progestin and adipoQ receptor family member 6	
PISD	−0.27421	0.001455	0.011557	−3.28858	Down	phosphatidylserine decarboxylase	
TNFSF12	−0.28316	0.001722	0.012942	−3.2349	Down	TNF superfamily member 12	
MAP11	−0.31678	0.001495	0.011825	−3.2801	Down	microtubule associated protein 11	
IQCE	−0.31488	0.001787	0.013302	−3.22309	Down	IQ motif containing E	
ADGRE5	−0.39217	0.001542	0.012114	−3.27021	Down	adhesion G protein-coupled receptor E5	
SCAP	−0.32181	0.001592	0.012352	−3.26013	Down	SREBF chaperone	
NECTIN1	−0.4398	0.002047	0.014673	−3.17924	Down	nectin cell adhesion molecule 1	
CDK5RAP3	−0.34828	0.001582	0.01231	−3.26209	Down	CDK5 regulatory subunit associated protein 3	
DAPK2	−0.30512	0.001699	0.012832	−3.23936	Down	death associated protein kinase 2	
PLAGL2	−0.27659	0.001719	0.012929	−3.23551	Down	PLAG1 like zinc finger 2	
ZDHHC18	−0.29438	0.001667	0.012699	−3.24539	Down	zinc finger DHHC-type containing 18	
CD4	−0.29252	0.001675	0.012725	−3.24392	Down	CD4 molecule	
ATG16L2	−0.47049	0.001688	0.012792	−3.24146	Down	autophagy related 16 like 2	
GBGT1	−0.30319	0.002212	0.015431	−3.15412	Down	globoside alpha-1,3-N-acetylgalactosaminyltransferase 1 (FORS blood group)	
SKIV2L	−0.32579	0.001807	0.013394	−3.2196	Down	Ski2 like RNA helicase	
TTC4	−0.34101	0.003031	0.019228	−3.05037	Down	tetratricopeptide repeat domain 4	
RAD54L2	−0.37561	0.002087	0.014855	−3.17303	Down	RAD54 like 2	
PI4K2A	−0.26735	0.00229	0.015792	−3.14278	Down	phosphatidylinositol 4-kinase type 2 alpha	
JAK3	−0.30717	0.001914	0.013982	−3.20098	Down	Janus kinase 3	
FLT3	−0.35297	0.002633	0.017514	−3.09708	Down	fms related tyrosine kinase 3	
RNF31	−0.33297	0.002109	0.014969	−3.16959	Down	ring finger protein 31	
POMZP3	−0.4664	0.002875	0.018477	−3.06791	Down	POM121 and ZP3 fusion	
LMTK2	−0.26618	0.002202	0.015375	−3.15558	Down	lemur tyrosine kinase 2	
AOC3	−0.44883	0.002455	0.016657	−3.12003	Down	amine oxidase copper containing 3	
FANCA	−0.35504	0.002422	0.016499	−3.12447	Down	FA complementation group A	
WBP2	−0.31533	0.002039	0.014631	−3.1806	Down	WW domain binding protein 2	
OGFOD2	−0.38737	0.003433	0.020952	−3.00876	Down	2-oxoglutarate and iron dependent oxygenase domain containing 2	
ITIH4	−0.7173	0.003584	0.021602	−2.99422	Down	inter-alpha-trypsin inhibitor heavy chain 4	
ITGAM	−0.28014	0.002099	0.01493	−3.17112	Down	integrin subunit alpha M	
CHD8	−0.28304	0.002143	0.015097	−3.16436	Down	chromodomain helicase DNA binding protein 8	
DENND1A	−0.31755	0.002347	0.016118	−3.13479	Down	DENN domain containing 1A	
TRIM41	−0.26057	0.00236	0.016189	−3.13294	Down	tripartite motif containing 41	
NLRP1	−0.3423	0.002242	0.01557	−3.14971	Down	NLR family pyrin domain containing 1	
MMP25	−0.48039	0.002249	0.01561	−3.1487	Down	matrix metallopeptidase 25	
POPDC2	−0.32941	0.003559	0.021496	−2.99664	Down	popeye domain containing 2	
CPNE9	−0.66459	0.003856	0.022604	−2.96956	Down	copine family member 9	
LAG3	−0.46043	0.003252	0.020199	−3.02693	Down	lymphocyte activating 3	
NDRG2	−0.27747	0.003018	0.019177	−3.05186	Down	NDRG family member 2	
INPP5B	−0.31274	0.002511	0.016952	−3.11262	Down	inositol polyphosphate-5-phosphatase B	
BMF	−0.26451	0.002523	0.017008	−3.11107	Down	Bcl2 modifying factor	
INPP5D	−0.26393	0.002376	0.016265	−3.13076	Down	inositol polyphosphate-5-phosphatase D	
PAPLN	−0.52955	0.004162	0.023765	−2.94356	Down	papilin, proteoglycan like sulfated glycoprotein	
SLC27A3	−0.31556	0.002609	0.017417	−3.10008	Down	solute carrier family 27 member 3	
XKR8	−0.26542	0.002565	0.017192	−3.10567	Down	XK related 8	
DOP1B	−0.26995	0.002714	0.017839	−3.08698	Down	DOP1 leucine zipper like protein B	
GMIP	−0.3929	0.002527	0.017026	−3.11057	Down	GEM interacting protein	
PNKD	−0.29826	0.002802	0.018154	−3.07643	Down	PNKD metallo-beta-lactamase domain containing	
ZNF264	−0.26952	0.003525	0.021391	−2.99983	Down	zinc finger protein 264	
PLXNA2	−0.4063	0.003836	0.022539	−2.97125	Down	plexin A2	
TAF8	−0.30568	0.00284	0.018306	−3.07201	Down	TATA-box binding protein associated factor 8	
GYS1	−0.30134	0.002951	0.018854	−3.05932	Down	glycogen synthase 1	
SLC1A4	−0.36474	0.003534	0.021412	−2.99901	Down	solute carrier family 1 member 4	
SLC15A3	−0.28569	0.002848	0.01835	−3.07109	Down	solute carrier family 15 member 3	
TLE3	−0.32824	0.002748	0.017961	−3.08292	Down	TLE family member 3, transcriptional corepressor	
SLC16A5	−0.31497	0.003097	0.01951	−3.04325	Down	solute carrier family 16 member 5	
OGDH	−0.32269	0.002818	0.018207	−3.07457	Down	oxoglutarate dehydrogenase	
RTN1	−0.33417	0.003191	0.019886	−3.03327	Down	reticulon 1	
THBS3	−0.34867	0.003237	0.020126	−3.0284	Down	thrombospondin 3	
BCL3	−0.36376	0.003035	0.019247	−3.04991	Down	BCL3 transcription coactivator	
ADCY4	−0.36448	0.003609	0.021672	−2.99194	Down	adenylatecyclase 4	
ZNF530	−0.32785	0.004519	0.025209	−2.91541	Down	zinc finger protein 530	
ZMYND15	−0.48374	0.004583	0.02545	−2.9106	Down	zinc finger MYND-type containing 15	
AMY1B	−0.72088	0.005292	0.028186	−2.86091	Down	amylase alpha 1B	
ZNF417	−0.30434	0.003838	0.022543	−2.97107	Down	zinc finger protein 417	
GTF2IRD2	−0.3548	0.004795	0.02631	−2.89503	Down	GTF2I repeat domain containing 2	
PRSS21	−0.78843	0.005521	0.02903	−2.84617	Down	serine protease 21	
CADM4	−0.3036	0.005273	0.028121	−2.86213	Down	cell adhesion molecule 4	
HIP1	−0.45352	0.003301	0.020406	−3.02192	Down	huntingtin interacting protein 1	
STIMATE-MUSTN1	−0.54123	0.005515	0.029012	−2.8465	Down	STIMATE-MUSTN1 readthrough	
KCTD11	−0.2781	0.004312	0.024369	−2.93148	Down	potassium channel tetramerization domain containing 11	
ZNF70	−0.57012	0.005544	0.029142	−2.84472	Down	zinc finger protein 70	
HIVEP3	−0.39006	0.004369	0.024627	−2.92696	Down	HIVEP zinc finger 3	
LOC100129697	−0.60899	0.005981	0.030664	−2.81815	Down	uncharacterized LOC100129697	
CIITA	−0.43886	0.003378	0.020711	−3.01412	Down	class II major histocompatibility complex transactivator	
EPOR	−0.27146	0.004472	0.025006	−2.91898	Down	erythropoietin receptor	
SHISA4	−0.64173	0.005489	0.028917	−2.84818	Down	shisa family member 4	
STIMATE	−0.30689	0.006148	0.031273	−2.80846	Down	STIM activating enhancer	
ADAT1	−0.27445	0.004127	0.023668	−2.94646	Down	adenosine deaminasetRNA specific 1	
TTLL11	−0.30463	0.006288	0.031761	−2.80055	Down	tubulin tyrosine ligase like 11	
H6PD	−0.40411	0.00368	0.021976	−2.9853	Down	hexose-6-phosphate dehydrogenase/glucose 1-dehydrogenase	
RAPGEFL1	−0.46135	0.004927	0.02685	−2.88567	Down	Rap guanine nucleotide exchange factor like 1	
TMEM63B	−0.31929	0.004159	0.023761	−2.94384	Down	transmembrane protein 63B	
PSPN	−0.32479	0.006167	0.031326	−2.80738	Down	persephin	
HLA-DQA1	−1.05471	0.004016	0.023213	−2.95571	Down	major histocompatibility complex, class II, DQ alpha 1	
TRAPPC9	−0.31907	0.004124	0.023668	−2.94672	Down	trafficking protein particle complex 9	
CCNJL	−0.30896	0.00384	0.022544	−2.97093	Down	cyclin J like	
MAP1A	−0.48755	0.005854	0.030316	−2.8257	Down	microtubule associated protein 1A	
PPP1R12B	−0.32586	0.003774	0.022328	−2.9768	Down	protein phosphatase 1 regulatory subunit 12B	
DNMBP	−0.36414	0.004698	0.025936	−2.9021	Down	dynamin binding protein	
CLEC17A	−0.48511	0.005274	0.028121	−2.86207	Down	C-type lectin domain containing 17A	
PACS1	−0.35178	0.003745	0.022242	−2.97938	Down	phosphofurin acidic cluster sorting protein 1	
TRIM25	−0.26502	0.00377	0.022316	−2.97712	Down	tripartite motif containing 25	
DSP	−1.74666	0.007097	0.034701	−2.75765	Down	desmoplakin	
PHF7	−0.33686	0.006229	0.031548	−2.80388	Down	PHD finger protein 7	
ADGRG3	−0.36957	0.003871	0.022639	−2.96821	Down	adhesion G protein-coupled receptor G3	
SULT1A2	−0.76588	0.007036	0.034482	−2.76074	Down	sulfotransferase family 1A member 2	
SPIB	−0.38425	0.004951	0.026919	−2.884	Down	Spi-B transcription factor	
DRICH1	−0.66714	0.007466	0.036148	−2.73956	Down	aspartate rich 1	
ZNF445	−0.34371	0.004446	0.024908	−2.921	Down	zinc finger protein 445	
USP19	−0.27666	0.004041	0.023332	−2.95362	Down	ubiquitin specific peptidase 19	
STK36	−0.29142	0.005018	0.027206	−2.87931	Down	serine/threonine kinase 36	
CDKN2A	−0.69258	0.007729	0.037039	−2.72716	Down	cyclin dependent kinase inhibitor 2A	
METTL7A	−0.27873	0.004291	0.024292	−2.93314	Down	methyltransferase like 7A	
TTLL3	−0.40446	0.004358	0.024578	−2.92787	Down	tubulin tyrosine ligase like 3	
C19orf84	−0.73685	0.007933	0.037785	−2.71782	Down	chromosome 19 open reading frame 84	
ARHGAP31	−0.30623	0.005156	0.02773	−2.86992	Down	Rho GTPase activating protein 31	
C2CD2L	−0.26804	0.004428	0.024824	−2.9224	Down	C2CD2 like	
PLEKHG3	−0.35043	0.00427	0.024204	−2.93484	Down	pleckstrin homology and RhoGEF domain containing G3	
GBF1	−0.35336	0.004328	0.02444	−2.93017	Down	golgibrefeldin A resistant guanine nucleotide exchange factor 1	
SLC6A16	−0.36797	0.006412	0.032156	−2.79366	Down	solute carrier family 6 member 16	
VARS2	−0.38504	0.005076	0.027413	−2.87536	Down	valyl-tRNAsynthetase 2, mitochondrial	
PLEKHM1	−0.31734	0.004414	0.024784	−2.92345	Down	pleckstrin homology and RUN domain containing M1	
SMIM34A	−0.72037	0.008217	0.038787	−2.70514	Down	small integral membrane protein 34A	
MARK4	−0.3283	0.005433	0.028678	−2.85174	Down	microtubule affinity regulating kinase 4	
SLC38A7	−0.28303	0.005751	0.029924	−2.83191	Down	solute carrier family 38 member 7	
SLC25A35	−0.2993	0.006192	0.031428	−2.80596	Down	solute carrier family 25 member 35	
VAT1	−0.27067	0.004901	0.026728	−2.88746	Down	vesicle amine transport 1	
C15orf39	−0.40438	0.004391	0.024697	−2.92529	Down	chromosome 15 open reading frame 39	
NF2	−0.28694	0.005349	0.028401	−2.8572	Down	neurofibromin 2	
BSCL2	−0.26614	0.007512	0.036312	−2.73739	Down	BSCL2 lipid droplet biogenesis associated, seipin	
VDR	−0.2684	0.005304	0.028225	−2.86013	Down	vitamin D receptor	
PADI4	−0.44486	0.004656	0.025758	−2.90517	Down	peptidyl arginine deiminase 4	
ABCG1	−0.29576	0.005169	0.027735	−2.86904	Down	ATP binding cassette subfamily G member 1	
ABTB2	−0.44053	0.008044	0.038142	−2.71281	Down	ankyrin repeat and BTB domain containing 2	
SLC16A13	−0.28493	0.007676	0.036885	−2.72966	Down	solute carrier family 16 member 13	
FHOD1	−0.31983	0.00504	0.027298	−2.87781	Down	formin homology 2 domain containing 1	
ARID1A	−0.31182	0.004979	0.027052	−2.88206	Down	AT-rich interaction domain 1A	
GPR157	−0.33012	0.007318	0.03555	−2.74675	Down	G protein-coupled receptor 157	
DAAM2	−1.17025	0.008639	0.040251	−2.68706	Down	dishevelled associated activator of morphogenesis 2	
INCA1	−0.60309	0.009868	0.04423	−2.63853	Down	inhibitor of CDK, cyclin A1 interacting protein 1	
TIGD3	−0.368	0.006217	0.031506	−2.80456	Down	tigger transposable element derived 3	
ENTPD2	−0.85818	0.009711	0.043759	−2.64442	Down	ectonucleoside triphosphate diphosphohydrolase 2	
ARHGEF5	−0.39012	0.009339	0.042517	−2.6587	Down	Rho guanine nucleotide exchange factor 5	
PLD2	−0.28888	0.005989	0.030695	−2.81768	Down	phospholipase D2	
POLR1A	−0.3791	0.006226	0.031544	−2.80404	Down	RNA polymerase I subunit A	
KIAA0556	−0.31343	0.005811	0.030142	−2.82826	Down	KIAA0556	
RAP1GAP2	−0.32685	0.005276	0.028122	−2.86195	Down	RAP1 GTPase activating protein 2	
ADD1	−0.26923	0.005269	0.02812	−2.86242	Down	adducin 1	
EHBP1L1	−0.35615	0.005468	0.028814	−2.84954	Down	EH domain binding protein 1 like 1	
FAM160A1	−0.7446	0.010863	0.047396	−2.60316	Down	family with sequence similarity 160 member A1	
SOGA1	−0.42952	0.007824	0.037374	−2.72278	Down	suppressor of glucose, autophagy associated 1	
TNFRSF21	−0.61534	0.01054	0.046315	−2.61431	Down	TNF receptor superfamily member 21	
NFAM1	−0.29308	0.005706	0.029771	−2.83465	Down	NFAT activating protein with ITAM motif 1	
CNOT3	−0.30586	0.005959	0.030608	−2.81943	Down	CCR4-NOT transcription complex subunit 3	
RSKR	−0.56514	0.010578	0.046421	−2.61296	Down	ribosomal protein S6 kinase related	
PLXNA4	−0.50001	0.010531	0.04631	−2.61461	Down	plexin A4	
CNTROB	−0.29089	0.006896	0.033945	−2.76788	Down	centrobin, centriole duplication and spindle assembly protein	
TBC1D10B	−0.26939	0.006094	0.031079	−2.81158	Down	TBC1 domain family member 10B	
NOXRED1	−0.34558	0.011102	0.048163	−2.59509	Down	NADP dependent oxidoreductase domain containing 1	
HYOU1	−0.30667	0.006028	0.030859	−2.81539	Down	hypoxia up-regulated 1	
SUPT5H	−0.27837	0.005938	0.030542	−2.82067	Down	SPT5 homolog, DSIF elongation factor subunit	
CLPB	−0.26559	0.008773	0.040714	−2.68144	Down	ClpB homolog, mitochondrial AAA ATPase chaperonin	
SIGLEC5	−0.30815	0.006387	0.032082	−2.79502	Down	sialic acid binding Ig like lectin 5	
NCR1	−0.46793	0.008972	0.041383	−2.67332	Down	natural cytotoxicity triggering receptor 1	
LRRC20	−0.28981	0.010509	0.046236	−2.6154	Down	leucine rich repeat containing 20	
MAP7D1	−0.30089	0.006066	0.030993	−2.81322	Down	MAP7 domain containing 1	
GHRL	−0.28653	0.008139	0.038516	−2.70858	Down	ghrelin and obestatinprepropeptide	
KDM6B	−0.4334	0.006275	0.031734	−2.80128	Down	lysine demethylase 6B	
CCDC17	−0.32392	0.009183	0.041985	−2.66487	Down	coiled-coil domain containing 17	
U2AF1L4	−0.26211	0.008081	0.038264	−2.71116	Down	U2 small nuclear RNA auxiliary factor 1 like 4	
CEACAM4	−0.30283	0.006663	0.033138	−2.78007	Down	CEA cell adhesion molecule 4	
TNS3	−0.28418	0.007707	0.036969	−2.72819	Down	tensin 3	
ADAP2	−0.27485	0.00825	0.038875	−2.70371	Down	ArfGAP with dual PH domains 2	
NLRX1	−0.2759	0.007971	0.037933	−2.71612	Down	NLR family member X1	
RABL2A	−0.30042	0.009208	0.042068	−2.66386	Down	RAB, member of RAS oncogene family like 2A	
PROSER3	−0.47732	0.009247	0.042198	−2.66234	Down	proline and serine rich 3	
MIDN	−0.29956	0.006943	0.034146	−2.76547	Down	midnolin	
DENND4B	−0.31441	0.006859	0.033811	−2.76981	Down	DENN domain containing 4B	
SORBS3	−0.40358	0.00832	0.039149	−2.70067	Down	sorbin and SH3 domain containing 3	
PLB1	−0.36701	0.007831	0.037384	−2.72248	Down	phospholipase B1	
DPEP3	−0.34095	0.009581	0.043304	−2.64937	Down	dipeptidase 3	
SEMA4B	−0.31138	0.007518	0.036312	−2.73711	Down	semaphorin 4B	
WASF2	−0.28093	0.007388	0.035832	−2.74331	Down	WASP family member 2	
NUCB1	−0.34217	0.007574	0.036492	−2.73442	Down	nucleobindin 1	
SH3BP2	−0.28179	0.007608	0.036623	−2.73283	Down	SH3 domain binding protein 2	
P2RX5	−0.37743	0.008788	0.040771	−2.68084	Down	purinergic receptor P2X 5	
SPN	−0.3575	0.007702	0.036969	−2.72842	Down	sialophorin	
KRTCAP2	−0.27178	0.010587	0.046448	−2.61265	Down	keratinocyte associated protein 2	
MRTFA	−0.32285	0.007914	0.037715	−2.7187	Down	myocardin related transcription factor A	
MBOAT7	−0.29593	0.007972	0.037933	−2.71605	Down	membrane bound O-acyltransferase domain containing 7	
PREX1	−0.30088	0.008158	0.038573	−2.70775	Down	phosphatidylinositol-3,4,5-trisphosphate dependent Rac exchange factor 1	
YLPM1	−0.28757	0.008714	0.040515	−2.68392	Down	YLP motif containing 1	
STK40	−0.26465	0.008277	0.038982	−2.70252	Down	serine/threonine kinase 40	
ARHGAP17	−0.33169	0.009266	0.04223	−2.66157	Down	Rho GTPase activating protein 17	
VNN3	−0.30084	0.008574	0.040052	−2.68977	Down	vanin 3	
SLC9A8	−0.26299	0.008652	0.040285	−2.68649	Down	solute carrier family 9 member A8	
RPGRIP1	−0.27294	0.010455	0.046081	−2.6173	Down	RPGR interacting protein 1	
LRRC4	−0.27126	0.009351	0.042557	−2.65825	Down	leucine rich repeat containing 4	
MAVS	−0.37027	0.009896	0.04429	−2.63751	Down	mitochondrial antiviral signaling protein	
DENND3	−0.29167	0.008663	0.040323	−2.68605	Down	DENN domain containing 3	
BAG6	−0.29939	0.008921	0.041228	−2.67539	Down	BCL2 associated athanogene 6	
STAT2	−0.3148	0.008975	0.041387	−2.67319	Down	signal transducer and activator of transcription 2	
TAGLN	−0.47191	0.01024	0.045397	−2.62496	Down	transgelin	
ARHGEF40	−0.30666	0.009153	0.041943	−2.66604	Down	Rho guanine nucleotide exchange factor 40	
HEATR6	−0.28169	0.011601	0.049803	−2.57873	Down	HEAT repeat containing 6	
DGAT2	−0.26818	0.009139	0.041901	−2.6666	Down	diacylglycerol O-acyltransferase 2	
PRKD2	−0.30467	0.009203	0.042065	−2.66408	Down	protein kinase D2	
AGER	−0.3384	0.009421	0.042727	−2.65554	Down	advanced glycosylation end-product specific receptor	
UBA7	−0.26693	0.009299	0.042344	−2.66029	Down	ubiquitin like modifier activating enzyme 7	
CAMKK1	−0.29228	0.011032	0.047934	−2.59743	Down	calcium/calmodulin dependent protein kinase kinase 1	
DLGAP4	−0.29263	0.009963	0.044477	−2.63502	Down	DLG associated protein 4	
REC8	−0.3437	0.010365	0.04582	−2.62047	Down	REC8 meiotic recombination protein	
TLN1	−0.4416	0.009401	0.042661	−2.65628	Down	talin 1	
QSOX1	−0.28813	0.009808	0.044044	−2.64077	Down	quiescin sulfhydryl oxidase 1	
ZNF692	−0.27196	0.010424	0.045995	−2.6184	Down	zinc finger protein 692	
CSF1R	−0.29204	0.00998	0.044538	−2.63442	Down	colony stimulating factor 1 receptor	
ABCC10	−0.3171	0.01149	0.04946	−2.58231	Down	ATP binding cassette subfamily C member 10	
ABTB1	−0.33918	0.010031	0.044708	−2.63253	Down	ankyrin repeat and BTB domain containing 1	
GALNS	−0.2628	0.011209	0.048514	−2.59152	Down	galactosamine (N-acetyl)-6-sulfatase	
PRRC2A	−0.42124	0.010301	0.045643	−2.62278	Down	proline rich coiled-coil 2A	
SLC11A1	−0.32785	0.010591	0.046449	−2.61254	Down	solute carrier family 11 member 1	
TEP1	−0.27091	0.011336	0.048899	−2.58735	Down	telomerase associated protein 1	
P2RX1	−0.33856	0.011621	0.049837	−2.57809	Down	purinergic receptor P2X 1

### Gene Ontology (GO) and pathway enrichment analyses of DEGs

To identify the pathways which had the most significant involvement with the genes identified, up regulated and down regulated genes are listed in Table 3 and Table 4. DEGs were submitted into ToppGene for GO and REACTOME pathway enrichment analysis. GO enrichment analysis revealed that in BP terms, the up regulated genes were mainly enriched in cell-cell signaling and reproductive process, whereas down regulated genes were mainly enriched in vesicle fusion and biological adhesion. In CC terms, up regulated genes were mainly enriched in integral component of plasma membrane and supramolecularfiber, whereas down regulated genes were mainly enriched in whole membrane and cell junction. In MF terms, up regulated genes were mainly enriched in signaling receptor activity and structural molecule activity, whereas down regulated genes were mainly enriched in lipid binding and GTPase binding. REACTOME pathway analysis demonstrated that up regulated genes were significantly enriched in signaling by GPCR and muscle contraction, whereas down regulated genes were mainly enriched in innate immune system and cytokine signaling in immune system.

**Table 3:** The enriched pathway terms of the up and down regulated differentially expressed genes

Pathway ID	Pathway Name	***P***-value	FDR B&H	FDR B&Y	Bonferroni	Gene Count	Gene
**Up regulated genes**							
1,269,543	Signaling by GPCR	3.16E-03	1.94E-01	1.00E+00	7.89E-01	16	OR2T29,NPBWR1,TRHR,AVP,GPR37,OR10J3,GRIN2B, RGS4,PTHLH,CRHR2, OR51E2,OR51E1,EGFR,OR6C70, CCL13,CCL19
1,269,868	Muscle contraction	5.98E-03	1.94E-01	1.00E+00	1.00E+00	5	MYH6,NPPC,KCNK4,ATP1A4,PLN
1,269,958	Digestion of dietary carbohydrate	5.00E-02	2.98E-01	1.00E+00	1.00E+00	1	AMY1A
1,457,790	Keratinization	1.22E-01	1.00E+00	3.53E-01	1.00E+00	3	KRT39,KRT20,KRT38
1,270,189	Biological oxidations	1.41E-01	3.67E-01	1.00E+00	1.00E+00	3	CYP2F1,AS3MT,UGT2A3
1,269,907	SLC-mediated transmembrane transport	4.87E-01	6.38E-01	1.00E+00	1.00E+00	2	AVP,G6PC
**Down regulated genes**							
1,269,203	Innate Immune System	1.03E-02	3.35E-01	1.00E+00	1.00E+00	16	CRISPLD2,DSP,OSCAR,C5AR2,SIGLEC14,ITGAX,MEFV,GAB2,MMP25,IGF2R,ATP6V0D1, DTX4,TLN1,PRKACA,ADGRE5,CLEC4C
1,269,310	Cytokine Signaling in Immune system	1.26E-01	5.91E-01	1.00E+00	1.00E+00	8	HLA-DQA1,ITGAX,GAB2,LGALS9,CIITA,TLN1,PRKACA,IL17RA
1,269,957	Metabolism of carbohydrates	1.31E-01	5.91E-01	1.00E+00	1.00E+00	4	NDST1,AMY1B,SLC9A1,PRKACA
1,269,876	Vesicle-mediated transport	1.41E-01	5.91E-01	1.00E+00	1.00E+00	7	VPS37C,HIP1,GABARAP,APOL1,ARF3,RAB36,IGF2R
1,269,507	Signaling by Rho GTPases	3.11E-01	6.03E-01	1.00E+00	1.00E+00	4	ARHGEF5,GMIP,ARHGAP1,ARAP3
1,269,649	Gene Expression	9.87E-01	9.89E-01	1.00E+00	1.00E+00	6	CDKN2A,POLR2A,ZNF385A,EIF3CL,BAZ2A,ZNF70

**Table 4:** The enriched GO terms of the up and down regulated differentially expressed genes

GO ID	CATEGORY	GO Name	***P*** Value	FDR B&H	FDR B&Y	Bonferroni	Gene Count	Gene
**Up regulated genes**								
GO:0007267	BP	cell-cell signaling	6.48E-03	1.84E-01	1.00E+00	1.00E+00	22	TRHDE,NPBWR1,MYH6,AVP,PPFIA2,IL17B,PCDHB10,PCDHB2,GRIA4,GRIN2B,DCC,RGS4,PTHLH,CRHR2,GJA1,CSNK1A1L,DKK1,EGFR,SNAP91,RIMS4,LRP2,CCL13
GO:0022414	BP	reproductive process	1.22E-02	1.99E-01	1.00E+00	1.00E+00	19	NPPC,AVP,BTBD18,MEIOB,RAD21L1,TEX15,PTHLH,GJA1,EGFR,SPATA16,MIF,OSR1,LRP2,HFM1,ACSBG2,SPATA22,ATP1A4,PRL,UTF1
GO:0005887	CC	integral component of plasma membrane	1.14E-03	1.17E-01	7.35E-01	3.43E-01	22	TRHDE,SEMA6B,RDH8,NPBWR1,TMC2,TRHR,GPR37,PCDHB10,PCDHB2,TM4SF5,GRIA4,GRIN2B,DCC,KCNK4,PTPRT,CRHR2,ADGRL4,GJA1,EGFR,LRFN5,UPK1B,ATP1A4
GO:0099512	CC	supramolecularfiber	1.65E-02	1.84E-01	1.00E+00	1.00E+00	17	ABRA,DNAH3,MYH6,MYOT,NRAP,TMC2,MYH13,KIF1A,GRIN2B,GJA1,KRT39,MFAP2,TRIM55,LRP2,KRT20,KRT38,PTPN20
GO:0038023	MF	signaling receptor activity	1.59E-03	1.30E-01	8.79E-01	7.75E-01	22	OR2T29,NPBWR1,MYOT,TRHR,GPR37,OR10J3,GRIA4,GRIN2B,DCC,PTPRT,CRHR2,ADGRL4,MTRNR2L1,OR51E2,MTRNR2L6,OR51E1,DKK1,EGFR,OR6C70,LRP2,ADGRF4,FCAMR
GO:0005198	MF	structural molecule activity	3.67E-02	2.51E-01	1.00E+00	1.00E+00	10	MYOT,EPB41L4B,MRPL12,PPFIA2,EMILIN3,KRT39,MFAP2,UPK1B,KRT20,KRT38
**Down regulated genes**								
GO:0006906	BP	vesicle fusion	1.55E-04	7.94E-02	6.71E-01	4.06E-01	17	CRISPLD2,DSP,RIMS3,OSCAR,SIGLEC14,DYSF,ITGAX,ITIH4,GAB2,LGALS9,MMP25,IGF2R,SCAMP5,TLN1,ADGRE5,TNFAIP2,CLEC4C
GO:0022610	BP	biological adhesion	2.52E-03	2.18E-01	1.00E+00	1.00E+00	19	CDKN2A,MYADM,DSP,HLA-DQA1,LAG3,SIGLEC14,DYSF,ITGAX,TNFRSF21,LGALS9,SLC9A1,TLN1,NECTIN1,SORBS3,AOC3,PLXNA4,PXN,ADGRE5,PLXNA2
GO:0098805	CC	whole membrane	4.42E-03	1.66E-01	1.00E+00	1.00E+00	21	VPS37C,MYADM,HIP1,TPCN2,DSP,HLA-DQA1,SIGLEC14,GABARAP,DYSF,ITGAX,MICALL1,MMP25,ARHGAP1,SLC9A1,IGF2R,ATP6V0D1,SCAMP5,PRKACA,ADGRE5,CLEC4C,ATG16L2
GO:0030054	CC	cell junction	2.83E-02	3.05E-01	1.00E+00	1.00E+00	15	MYADM,DSP,ARHGEF5,RIMS3,LASP1,SLC9A1,IGF2R,SCAMP5,TLN1,NECTIN1,SORBS3,PXN,ADGRE5,ALKBH6,PDZD3
GO:0008289	MF	lipid binding	2.91E-03	1.08E-01	7.31E-01	1.00E+00	13	HIP1,CPNE9,ARHGEF5,PAQR6,APOL1,DYSF,GAB2,MICALL1,SLC9A1,IGF2R,ARAP3,TLN1,APOL2
GO:0051020	MF	GTPase binding	5.51E-03	1.08E-01	7.31E-01	1.00E+00	10	ARHGEF5,RGL3,RIMS3,MICALL1,ARHGAP1,PRKACA,DAAM2,RIPOR1,RGL2,RAPGEFL1

*BP* Biological Process, *CC* Cellular Component and *MF* Molecular Functions

### PPI network construction and module analysis

**Fig. 3:**
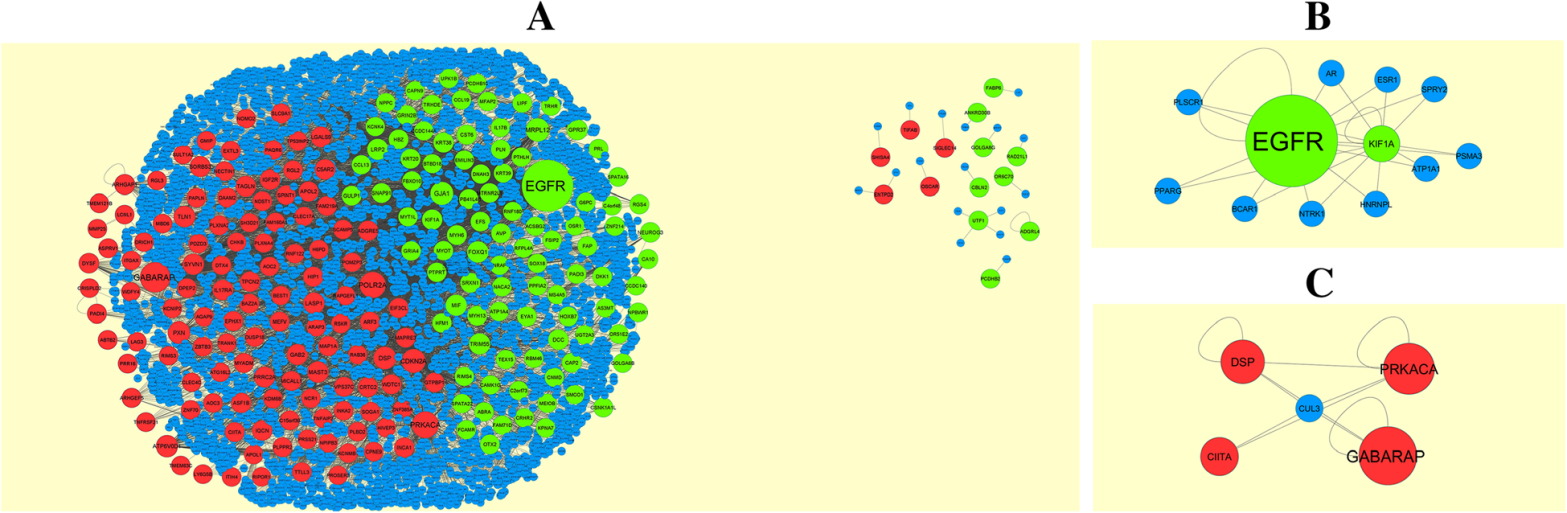
PPI network and the most significant modules of DEGs. **a** The PPI network of DEGs was constructed using Cytoscape. **b** The most significant module was obtained from PPI network with 12 nodes and 23 edges for up regulated genes. **c** The most significant module was obtained from PPI network with 5 nodes and 10 edges for down regulated genes. Up regulated genes are marked in green; Down regulated genes are marked in red

Interactions between the identified up and down regulated genes were reported by constructing a PPI network. In total, there were 5017 nodes and 8133 edges in the network (Fig.3a). According to node degree, betweenness centrality, stress centrality and closeness centrality levels, the top four hub nodes were: EGFR (degree, 1343; betweenness, 0.514227; stress, 4.35E+08; closeness, 0.441893), GRIN2B (degree, 195; betweenness, 0.008701; stress, 14,535,992; closeness, GJA1 (degree,170 0.03151; betweenness, 64,939,702; stress, 0.31364; closeness, 0.282164), CAP2 (degree, 108; betweenness, 0.004218; stress, 888,128; closeness, 0.325945), MIF (degree, 99; betweenness, 0.025028; stress, 7,627,912; closeness, 0.362124), POLR2A (degree, 512; betweenness, 0.095104; stress, 2E+08; closeness, 0.3738), PRKACA (degree, 350; betweenness, 0.104658; stress, 44,280,174; closeness, 0.378052), GABARAP (degree, 322; betweenness, 0.144708; stress, 36,234,824; closeness, 0.358401), TLN1 (degree, 223; betweenness, 0.027406; stress, 67,578,124; closeness, 0.35998), and PXN (degree, 173; betweenness, 0.029203; stress, 29,047,866; closeness,0.360318) and are listed in Table 5. Significant modules were subsequently constructed with 12 nodes and 23 edges for up regulated genes (Fig.3b) and 5 nodes and 10 edges for down regulated genes, which gained the highest PEWCC1 score (Fig.3c). Subsequent functional enrichment analysis revealed that the genes in these modules were mainly enriched in cell-cell signaling and cytokine signaling in immune system.

**Table 5:** Topology table for up and down regulated genes

Regulation	Node	Degree	Betweenness	Stress	Closeness
Up	EGFR	1343	0.514227	4.35E+08	0.441893
Up	GRIN2B	195	0.008701	14,535,992	0.282164
Up	GJA1	170	0.03151	64,939,702	0.31364
Up	CAP2	108	0.004218	888,128	0.325945
Up	MIF	99	0.025028	7,627,912	0.362124
Up	DCC	98	0.009465	15,166,420	0.286956
Up	PRL	89	0.001208	656,482	0.239894
Up	EYA1	72	0.003864	4,335,792	0.271944
Up	RGS4	63	0.003414	1,243,420	0.31129
Up	GRIA4	55	0.001352	431,502	0.292547
Up	CCL19	50	0.001236	1,457,894	0.1951
Up	DKK1	50	0.003569	4,044,234	0.25252
Up	PLN	43	0.003324	524,170	0.286083
Up	GPR37	41	0.021208	9,337,328	0.303261
Up	LRP2	39	0.023544	9,018,868	0.307583
Up	PTHLH	38	0.001269	1,103,574	0.257729
Up	PPFIA2	33	0.002618	3,942,042	0.269956
Up	KIF1A	31	0.011317	2,986,868	0.345485
Up	HOXB7	31	0.002165	2,106,446	0.268893
Up	MYOT	28	0.003103	475,758	0.275841
Up	OTX2	28	0.003016	868,076	0.271263
Up	NPPC	27	0.003211	986,326	0.247419
Up	KRT20	26	0.005296	5,544,830	0.279444
Up	MRPL12	25	0.050618	70,486,194	0.310399
Up	NPBWR1	23	4.01E-04	207,170	0.174777
Up	TRHR	22	4.10E-04	210,106	0.238152
Up	CCL13	20	0.003552	1,650,164	0.263645
Up	MFAP2	19	0.002005	451,990	0.228053
Up	TRIM55	18	0.021705	17,474,648	0.300609
Up	NEUROG3	18	0.011467	9,544,416	0.269256
Up	NRAP	18	0.001974	406,892	0.285085
Up	PTPRT	18	0.00104	208,946	0.324523
Up	OSR1	17	4.56E-04	195,068	0.226447
Up	EFS	17	0.003557	2,975,070	0.285232
Up	HBZ	17	0.006623	4,433,868	0.287818
Up	KCNK4	16	4.01E-04	117,736	0.247125
Up	AVP	16	0.006488	3,769,102	0.26391
Up	SOX18	16	8.04E-04	761,526	0.244028
Up	NACA2	15	0.002639	3,304,692	0.274822
Up	SRXN1	15	0.005715	9,265,020	0.276852
Up	UTF1	13	1	12	1
Up	RNF180	13	4.01E-04	186,722	0.235697
Up	KRT38	12	0.011531	15,515,646	0.273676
Up	FAP	12	0.005985	10,442,836	0.259379
Up	GULP1	12	0.001542	1,584,446	0.281416
Up	IL17B	12	4.02E-04	151,558	0.24843
Up	CRHR2	11	0.002005	1,044,220	0.245978
Up	RFPL4A	10	9.09E-06	2638	0.1894
Up	SNAP91	10	0.001514	2,227,728	0.265994
Up	ADGRL4	10	0	0	0
Up	FBXO10	10	0.002727	598,280	0.287801
Up	CST6	9	0.009382	6,670,420	0.28234
Up	PADI3	9	0.003682	1,684,994	0.267321
Up	LIPF	9	0.002462	1,222,458	0.239008
Up	CA10	8	0.00882	3,036,648	0.251628
Up	EPB41L4B	8	0.00328	3,044,266	0.280229
Up	OR51E2	8	0.002481	1,735,460	0.249674
Up	RIMS4	8	4.46E-04	187,102	0.255497
Up	MYT1L	8	1.71E-04	156,968	0.255523
Up	C4orf48	7	4.02E-04	410,864	0.238654
Up	EMILIN3	7	0.002789	890,368	0.2335
Up	CSNK1A1L	6	0.002373	3,707,900	0.264526
Up	ATP1A4	6	0.001778	1,343,858	0.269795
Up	OR6C70	6	0	0	1
Up	MEIOB	6	1.19E-04	55,294	0.237857
Up	UPK1B	5	0.001266	639,316	0.238883
Up	TEX15	5	8.47E-04	384,696	0.261104
Up	CAMK1G	5	0.001061	955,066	0.263353
Up	FOXQ1	5	0.02441	34,422,300	0.29497
Up	MYH13	4	0.007248	6,307,480	0.276193
Up	MYH6	4	0.006387	8,432,142	0.276223
Up	SPATA22	4	0.001497	616,922	0.255628
Up	TRHDE	4	0.004478	568,880	0.325562
Up	CCDC144A	4	0.001246	906,138	0.212789
Up	PCDHB10	4	8.03E-04	313,938	0.212698
Up	CNMD	4	0.001108	411,030	0.251134
Up	ABRA	4	0.001639	940,076	0.263019
Up	MS4A5	4	0	0	0.246088
Up	DNAH3	3	5.51E-04	333,004	0.269839
Up	MTRNR2L1	3	9.66E-04	646,606	0.269168
Up	KPNA7	3	4.63E-04	356,676	0.259541
Up	CBLN2	3	0	0	1
Up	FSIP2	3	4.14E-04	158,112	0.250276
Up	SPATA16	3	0	0	0.237426
Up	ZNF214	2	0.004409	2,022,086	0.235753
Up	CCDC140	2	4.01E-04	96,422	0.215353
Up	UGT2A3	2	8.95E-04	518,942	0.239952
Up	FAM71D	2	4.04E-04	236,600	0.24016
Up	GOLGA6B	2	0	0	0.221585
Up	C2orf73	2	8.02E-04	393,150	0.236301
Up	GOLGA8G	2	0	0	1
Up	SMCO1	1	4.28E-04	175,370	0.225341
Up	ANKRD30B	1	0	0	1
Up	FCAMR	1	0	0	0.225719
Up	HFM1	1	8.96E-05	46,366	0.226673
Up	BTBD18	1	0	0	0.306486
Up	ACSBG2	1	4.02E-04	116,692	0.247358
Up	KRT39	1	0	0	0.251806
Up	G6PC	1	0.001203	289,260	0.215428
Up	RBM46	1	1.99E-06	1374	0.252879
Up	PCDHB2	1	0	0	1
Up	AS3MT	1	0	0	0.210364
Up	RAD21L1	1	0	0	1
Up	CAPN9	1	2.61E-06	2714	0.211893
Up	FABP6	1	0	0	1
Down	POLR2A	512	0.095104	2E+08	0.3738
Down	PRKACA	350	0.104658	44,280,174	0.378052
Down	GABARAP	322	0.144708	36,234,824	0.358401
Down	TLN1	223	0.027406	67,578,124	0.35998
Down	PXN	173	0.029203	29,047,866	0.360318
Down	HIP1	146	0.007469	12,022,620	0.347944
Down	GAB2	118	0.014217	38,718,180	0.340041
Down	SYVN1	114	0.025941	12,050,404	0.307887
Down	KCNIP2	110	0.002849	14,186,360	0.253084
Down	ARHGAP1	106	0.011263	27,047,958	0.348406
Down	NECTIN1	102	0.003189	17,978,038	0.252469
Down	RGL2	88	0.003069	3,605,218	0.263534
Down	ASF1B	85	0.00926	21,237,598	0.2861
Down	IGF2R	78	0.010589	26,691,414	0.303021
Down	SLC9A1	75	0.009561	24,396,634	0.292461
Down	VPS37C	75	0.006908	5,646,832	0.29327
Down	MEFV	72	0.001361	31,003,730	0.301081
Down	CDKN2A	72	0.051693	14,128,370	0.321613
Down	MAP1A	63	0.008662	3,403,950	0.291879
Down	ARF3	60	0.004127	11,523,596	0.287784
Down	CIITA	59	0.006319	10,301,788	0.302892
Down	WDTC1	57	0.005909	19,161,622	0.278569
Down	MICALL1	53	0.005986	7,902,576	0.285919
Down	ITGAX	53	0.001212	13,088,030	0.238757
Down	SORBS3	51	0.016308	10,468,808	0.314749
Down	DSP	47	0.039735	2,314,856	0.347581
Down	BAZ2A	45	0.004013	13,101,300	0.289472
Down	MAPRE3	44	0.006813	4,554,084	0.28533
Down	CRTC2	44	0.004812	11,994,716	0.296709
Down	PADI4	43	0.002174	7,728,076	0.275765
Down	SPINT1	42	0.001717	6,257,478	0.263492
Down	ARHGEF5	40	0.003561	7,025,544	0.336483
Down	LASP1	40	0.021943	9,410,452	0.31303
Down	PDZD3	39	0.005273	6,150,510	0.249549
Down	HIVEP3	39	0.001859	4,355,942	0.26672
Down	TNFRSF21	36	0.001781	3,131,524	0.274883
Down	SOGA1	36	0.005248	10,217,032	0.291623
Down	MBD6	34	0.002017	1,737,436	0.231979
Down	ATP6V0D1	33	0.026324	1,957,202	0.303649
Down	IQCN	32	0.015047	5,650,026	0.283239
Down	IL17RA	30	0.020601	4,651,714	0.286807
Down	ABTB2	29	0.001793	4,772,698	0.279695
Down	INCA1	29	0.011463	2,924,018	0.276668
Down	MAST3	28	0.019115	4,567,048	0.308592
Down	BEST1	28	4.42E-04	3,684,990	0.260082
Down	PRSS21	28	0.001117	5,817,460	0.25749
Down	TPCN2	27	0.014228	4,144,602	0.284792
Down	LAG3	26	4.41E-06	6,746,636	0.238038
Down	GMIP	24	8.12E-04	3,636,310	0.263896
Down	CLEC4C	24	4.01E-04	725,910	0.175956
Down	TAGLN	23	0.016019	4,532,938	0.283771
Down	FAM219A	20	0.008105	916,962	0.287867
Down	NCR1	20	0.002234	349,872	0.271381
Down	PRRC2A	18	0.015874	2,767,592	0.307261
Down	KCNMB1	18	8.42E-04	2,984,594	0.257118
Down	EIF3CL	17	0.003808	1,129,844	0.284272
Down	H6PD	17	5.55E-04	3,133,402	0.259123
Down	MMP25	17	4.01E-04	2,598,252	0.238837
Down	ENTPD2	16	1	2,170,770	1
Down	DYSF	16	0.009592	704,606	0.292839
Down	LGALS9	16	0.01981	1,417,206	0.288134
Down	DPEP2	16	0.007617	1,500,838	0.260885
Down	POMZP3	14	8.35E-05	836,800	0.254857
Down	EXTL3	13	0.004948	611,924	0.27899
Down	EPHX1	13	0.006221	2,003,020	0.279491
Down	C15orf39	12	0.00794	610,110	0.291793
Down	PLXNA2	12	0.009415	1,162,970	0.277268
Down	PLPPR2	12	0.006567	1,448,934	0.253961
Down	MYADM	11	0.007222	952,256	0.283771
Down	GTPBP1	11	0.005922	1,877,862	0.299976
Down	ADGRE5	10	0.014659	888,184	0.289203
Down	SULT1A2	10	0.00172	1,783,464	0.247973
Down	INKA2	10	0.00214	1,184,052	0.279616
Down	NDST1	10	0.001942	1,716,840	0.257383
Down	TTLL3	9	0.002348	134,132	0.261694
Down	DRICH1	9	0.002408	1,799,342	0.237302
Down	APOL2	9	0.007736	489,254	0.289489
Down	ZNF70	9	0.001286	3,985,632	0.223763
Down	ASPRV1	9	0.00395	1,038,668	0.290552
Down	PLXNA4	8	0.001243	473,354	0.262534
Down	FAM160A1	8	4.10E-04	882,848	0.241043
Down	PLBD2	8	0.002764	1,265,098	0.278057
Down	RNF122	8	0.001044	1,049,362	0.265329
Down	PAQR6	8	4.10E-04	868,456	0.23693
Down	CHKB	7	0	602,254	0.217363
Down	CRISPLD2	7	3.80E-06	943,864	0.256153
Down	DUSP18	7	0.002971	325,170	0.315506
Down	TRANK1	7	4.02E-04	801,248	0.245821
Down	TP53INP2	6	1.70E-07	703,508	0.247198
Down	ARAP3	6	4.92E-04	286,916	0.268169
Down	TNFAIP2	6	0.001439	633,692	0.254324
Down	APOL1	5	6.25E-04	48,364	0.233895
Down	PRR16	5	0.001298	158,878	0.26692
Down	ITIH4	5	4.46E-04	356,728	0.266806
Down	SIGLEC14	5	0	273,222	1
Down	C5AR2	5	0.019084	54,700	0.270983
Down	DTX4	5	8.06E-04	445,430	0.256483
Down	SCAMP5	5	0.002526	469,900	0.229016
Down	SH3D21	5	1.03E-04	675,458	0.314928
Down	LY6G5B	4	6.87E-04	43,440	0.259947
Down	TMEM121B	4	0	217,638	0.1804
Down	AOC3	4	0.002207	251,106	0.268748
Down	DAAM2	3	0.00264	78,488	0.259838
Down	ZBTB3	3	0.006264	225,562	0.267838
Down	LCNL1	3	9.23E-05	198,214	0.235286
Down	RIMS3	2	4.16E-04	11,332	0.223944
Down	ZNF385A	2	6.60E-04	3498	0.264948
Down	RIPOR1	2	6.43E-04	1512	0.277964
Down	KDM6B	2	0.003441	321,702	0.271411
Down	PAPLN	2	0.001857	53,928	0.244759
Down	RAB36	2	4.01E-04	4276	0.188797
Down	NOMO2	2	5.94E-04	78	0.280182
Down	AOC2	2	5.51E-04	3886	0.250113
Down	PROSER3	2	4.60E-05	2	0.23941
Down	WDFY4	2	2.38E-06	184,474	0.236975
Down	ATG16L2	2	0.001016	322,434	0.299651
Down	RAPGEFL1	2	7.68E-05	9928	0.260599
Down	CPNE9	1	9.19E-05	0	0.23639
Down	RSKR	1	4.69E-06	0	0.247739
Down	RGL3	1	0	0	0.234621
Down	SHISA4	1	0	0	1
Down	TMEM63C	1	0	0	0.24945
Down	NPIPB3	1	8.84E-06	0	0.229639
Down	CLEC17A	1	0	0	0.209331
Down	TIFAB	1	0	0	1
Down	OSCAR	1	0	0	1
Down	AGAP9	1	0	0	0.236256

### Prediction of key miRNAs

**Fig. 4:**
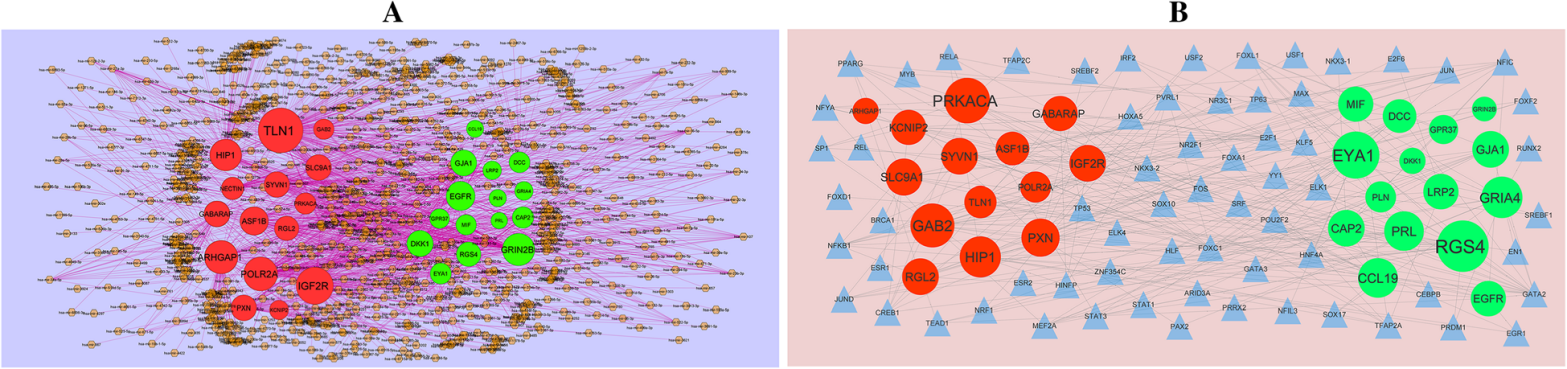
**a** Target gene - miRNA regulatory network between target genes and miRNAs. **b** Target gene - TF regulatory network between target genes and TFs. Up regulated genes are marked in green; down regulated genes are marked in red; The chocolate color triangle nodes represent the key miRNAs; the blue color triangle nodes represent the key miRNAs

The regulatory relationships between the target genes and their miRNAs were established using Cytoscape, which showed that the single gene was regulated by multiple miRNAs is shown in Fig.4a. Subsequently, 106 miRNAs (ex, hsa-mir-4257) targeting GRIN2B, 83 miRNAs (ex, hsa-mir-564) targeting EGFR, 66 miRNAs (ex, hsa-mir-587) targeting DKK1, 64 miRNAs (ex, hsa-mir-941) targeting GJA1, 54 miRNAs (ex, hsa-mir-561-3p) targeting RGS4, 190 miRNAs (ex, hsa-mir-4300) targeting TLN1, 139 miRNAs (ex, hsa-mir-5694) targeting IGF2R, 117 miRNAs (ex, hsa-mir-378b) targeting POLR2A, 113 miRNAs (ex, hsa-mir-3918) targeting ARHGAP1 and 102 miRNAs (ex, hsa-mir-6719-3p) targeting HIP1, and were verified in miRNet database are listed in Table 6. Integrating with the result of REACTOME pathway analysis, it was indicated that these key target genes - miRNA network was mainly involved in the signaling by GPCR and innate immune system.

**Table 6:** miRNA - target gene and TF - target gene interaction

Regulation	Target Genes	Degree	MicroRNA	Regulation	Target Genes	Degree	TF
UP	COL12A1	110	hsa-mir-526b-5p	UP	SUZ12	164	PAX3
UP	COL1A2	81	hsa-mir-1254	UP	AR	162	ANKRD18B
UP	SIX4	73	hsa-mir-1909-5p	UP	STAT3	147	WT1
UP	GNG12	69	hsa-mir-3194-3p	UP	TP53	138	ADCYAP1R1
UP	HAS2	63	hsa-mir-30e-5p	UP	EGR1	137	KIF1A
UP	COL3A1	58	hsa-mir-4691-3p	UP	SMAD4	130	WFDC1
UP	PRKAA2	57	hsa-mir-3619-5p	UP	NANOG	129	MAGI1
UP	DKK1	56	hsa-mir-579-3p	UP	REST	123	LHX2
UP	EGFR	56	hsa-mir-494-3p	UP	TP63	121	CYR61
UP	RGS4	50	hsa-mir-526a	UP	POU5F1	121	PDE10A
UP	CSRNP3	50	hsa-mir-338-3p	UP	MTF2	120	ESRP2
UP	SPRY4	50	hsa-mir-21-5p	UP	TCF4	115	EBF3
UP	GJA1	50	hsa-mir-185-5p	UP	MYC	114	DDC
UP	PRRX1	49	hsa-mir-888-3p	UP	HNF4A	114	EGFR
UP	GRIN2B	48	hsa-let-7a-5p	UP	RUNX2	92	CELF4
UP	GDA	44	hsa-mir-548d-5p	UP	RUNX1	87	BCAR1
UP	BDKRB2	40	hsa-let-7f-5p	UP	SETDB1	87	LMO1
UP	HOXB8	38	hsa-mir-30d-3p	UP	RNF2	85	SIX1
UP	EFEMP1	37	hsa-let-7a-2-3p	UP	SPI1	84	PTHLH
UP	PGR	35	hsa-mir-320b	UP	EZH2	81	BARX2
UP	KIF1A	33	hsa-mir-125a-5p	UP	PPARD	79	EXOC3L4
UP	CAP2	33	hsa-let-7c-5p	UP	SIN3B	79	SPRY4
UP	EYA1	30	hsa-mir-133a-3p	UP	SMARCA4	78	FOXQ1
UP	NPNT	30	hsa-mir-1304-3p	UP	MITF	76	GNG12
UP	MAGI1	29	hsa-let-7f-1-3p	UP	BMI1	76	LRFN5
UP	MICU3	28	hsa-mir-182-5p	UP	GATA2	75	ZSCAN10
UP	PDE10A	28	hsa-mir-1288-3p	UP	TCF3	74	IGSF21
UP	SYT4	27	hsa-mir-320a	UP	SALL4	74	SULF1
UP	GULP1	26	hsa-mir-532-5p	UP	PPARG	73	PRRX1
UP	HOXB7	26	hsa-mir-2277-3p	UP	JARID2	72	DMRTA2
UP	AMER2	25	hsa-mir-369-3p	UP	SOX9	72	PTPRT
UP	MTRNR2L1	25	hsa-mir-4793-3p	UP	TRIM28	71	RGS20
UP	SLC9A2	25	hsa-mir-15b-5p	UP	FLI1	69	MRO
UP	EEF1G	25	hsa-mir-106b-5p	UP	ESR1	67	RERG
UP	FBXO10	24	hsa-mir-23c	UP	KLF4	67	RFX4
UP	FAXC	24	hsa-mir-135a-5p	UP	SRY	67	SRXN1
UP	ADAMTS9	24	hsa-mir-181a-2-3p	UP	RCOR3	64	C8orf4
UP	FOXQ1	24	hsa-mir-503-5p	UP	GATA1	64	COL12A1
UP	EPB41L4B	23	hsa-mir-141-5p	UP	E2F1	64	GJA1
UP	SH3GL2	23	hsa-mir-548 am-5p	UP	YAP1	63	HAS2
UP	CLVS2	23	hsa-mir-32-3p	UP	POU3F2	63	HOXD8
UP	GPR158	22	hsa-mir-302d-3p	UP	CREM	62	OSR1
UP	SOX2	22	hsa-mir-361-5p	UP	PBX1	62	OTX2
UP	FSTL5	21	hsa-mir-5100	UP	RAD21	62	PKNOX2
UP	LRRTM2	21	hsa-mir-125b-5p	UP	FOXP1	62	TRAPPC5
UP	FAP	21	hsa-mir-30a-5p	UP	EP300	61	EN1
UP	MIF	21	hsa-mir-139-3p	UP	TET1	58	HES5
UP	GABRA4	21	hsa-mir-23a-3p	UP	SMAD3	56	OVOL1
UP	TMEM121	21	hsa-mir-3065-3p	UP	NR3C1	55	DKK1
UP	GPR37	21	hsa-mir-192-5p	UP	EED	55	TBX20
UP	SNAP91	21	hsa-mir-29a-3p	UP	OLIG2	53	DPP10
UP	BCAR1	21	hsa-mir-1296-5p	UP	ERG	50	MYRIP
UP	PPFIA2	20	hsa-mir-140-3p	UP	FOXA2	48	MNX1
UP	LAMA4	20	hsa-mir-30e-3p	UP	PHC1	48	ZFPM2
UP	AREG	20	hsa-mir-449b-5p	UP	KDM5B	46	EEF1G
UP	PAX3	20	hsa-mir-5690	UP	TFAP2A	46	NOS1
UP	PRSS12	19	hsa-mir-1260a	UP	TFAP2C	44	EYA1
UP	HHIP	19	hsa-mir-488-3p	UP	PRDM14	43	TAC4
UP	SULF1	19	hsa-mir-130a-3p	UP	JUN	42	PRSS12
UP	PTPRT	19	hsa-mir-518c-5p	UP	ZNF281	41	PDLIM4
UP	PTHLH	18	hsa-mir-376a-5p	UP	CREB1	40	ASCL1
UP	TEAD4	18	hsa-mir-520c-3p	UP	RBPJ	39	CAMK1G
UP	URGCP-MRPS24	16	hsa-mir-933	UP	CUX1	39	TRIM55
UP	CBLN2	16	hsa-mir-3065-3p	UP	NFE2L2	38	LRP2
UP	TRHDE	16	hsa-let-7i-5p	UP	ASH2L	37	RPE65
UP	SLC30A3	16	hsa-let-7 g-5p	UP	SCLY	36	KCNIP1
UP	NPPC	16	hsa-mir-497-5p	UP	BACH1	35	EFEMP1
UP	PPP2R2C	16	hsa-mir-219a-5p	UP	RCOR1	34	OR51E2
UP	ZFPM2	16	hsa-mir-4705	UP	CTNNB1	33	KRT39
UP	SIX1	16	hsa-mir-139-5p	UP	EWSR1	32	BDKRB2
UP	GABRB3	16	hsa-mir-582-3p	UP	SMAD2	32	CAP2
UP	SEMA6B	15	hsa-mir-221-5p	UP	ZNF217	31	THSD4
UP	EBF3	15	hsa-mir-629-5p	UP	SOX17	31	UTF1
UP	THSD4	15	hsa-mir-612	UP	DMRT1	30	DCC
UP	MFAP2	15	hsa-mir-503-5p	UP	DNAJC2	30	MEGF10
UP	ESRP2	15	hsa-mir-98-5p	UP	RELA	30	SIX4
UP	RASSF9	15	hsa-mir-588	UP	FOXP2	28	CBLN2
UP	TIMP4	14	hsa-mir-1343-3p	UP	ATF3	28	PDPN
UP	PDE1A	14	hsa-mir-369-3p	UP	NR0B1	28	SLC30A3
UP	RERG	14	hsa-mir-671-5p	UP	EOMES	28	WIF1
UP	MNX1	14	hsa-mir-548z	UP	TAL1	27	AS3MT
UP	MYT1L	14	hsa-mir-199a-3p	UP	TFCP2L1	27	CACNA1G
UP	RFX6	14	hsa-mir-33a-3p	UP	KLF1	27	CCL17
UP	TRAPPC5	13	hsa-mir-1269b	UP	TBX3	27	HHIP
UP	TMEM151A	13	hsa-let-7e-5p	UP	ARNT	26	PADI3
UP	LRP2	13	hsa-mir-1305	UP	ELF5	24	HFM1
UP	GRM5	13	hsa-mir-22-3p	UP	CEBPB	24	NPY
UP	ITGBL1	13	hsa-mir-16-2-3p	UP	MYCN	21	EPB41L4B
UP	LHX2	13	hsa-mir-3661	UP	YY1	21	RIMS4
UP	RGS20	13	hsa-mir-4659a-3p	UP	ESRRB	21	SMTNL2
UP	CDH18	13	hsa-mir-550a-3p	UP	ELF1	20	SPDEF
UP	C4orf48	12	hsa-mir-497-5p	UP	NR1I2	19	CBLC
UP	LARP6	12	hsa-mir-126-3p	UP	HSF1	19	HOXB7
UP	GRIA4	12	hsa-mir-377-3p	UP	ELK1	19	NEUROG3
UP	TEX15	12	hsa-mir-1304-5p	UP	NACC1	19	REN
UP	SCG3	12	hsa-mir-448	UP	MYBL2	19	SP9
UP	LRRC8E	12	hsa-mir-500a-5p	UP	ZFX	19	TMEM121
UP	SPDEF	12	hsa-mir-939-5p	UP	E2F4	18	AMER2
UP	KCNT2	11	hsa-mir-548o-3p	UP	AHR	18	CNTNAP4
UP	OTOGL	11	hsa-mir-548n	UP	CTCF	18	GRIN2B
UP	TMEM145	11	hsa-mir-1913	UP	FOXO3	17	BTC
UP	RIMS4	11	hsa-mir-326	UP	SIN3A	17	GPR158
UP	SRXN1	11	hsa-mir-195-5p	UP	CDX2	17	HOXB8
UP	TRPA1	11	hsa-mir-320d	UP	MEF2A	17	HSD11B2
UP	SELE	11	hsa-mir-17-5p	UP	CNOT3	17	SCN3B
UP	HES5	11	hsa-mir-4511	UP	SOX11	16	LRRC74A
UP	C3orf80	10	hsa-mir-369-3p	UP	CEBPD	16	PDE1A
UP	PKNOX2	10	hsa-mir-490-3p	UP	TCF7	16	PPP2R2C
UP	EPN3	10	hsa-mir-1825	UP	LYL1	16	SLC9A2
UP	HRCT1	10	hsa-mir-374a-5p	UP	STAT5A	15	CYP39A1
UP	SERPIND1	10	hsa-mir-452-5p	UP	HTT	15	PGR
UP	CRHR2	10	hsa-mir-31-5p	UP	LMO2	15	RNF180
UP	MIPOL1	10	hsa-mir-651-5p	UP	SREBF2	15	TMEM151A
UP	MYH6	9	hsa-mir-301a-3p	UP	PAX6	14	ACSBG2
UP	PMCH	9	hsa-mir-30a-5p	UP	DROSHA	14	ANO4
UP	C8orf34	9	hsa-mir-302c-3p	UP	GFI1B	14	FFAR1
UP	ANO4	9	hsa-mir-29b-3p	UP	TTF2	14	NR0B2
UP	SCEL	9	hsa-mir-671-5p	UP	STAT4	14	PRLHR
UP	RHOJ	9	hsa-mir-103a-3p	UP	GATA3	13	BCO1
UP	PDLIM4	9	hsa-mir-1270	UP	CCND1	13	COL1A2
UP	CYP39A1	9	hsa-mir-1179	UP	ZFP42	13	EFS
UP	SLC26A3	9	hsa-mir-494-3p	UP	GATA4	13	MMP27
UP	OVOL1	9	hsa-mir-15a-5p	UP	MEIS1	11	DNAI1
UP	BTC	9	hsa-mir-194-5p	UP	TCF7L2	11	ELAVL3
UP	CNTFR	9	hsa-mir-708-5p	UP	STAT1	11	FOLH1
UP	MRPL12	9	hsa-mir-98-5p	UP	IRF8	11	GDA
UP	SP9	8	hsa-mir-1343-3p	UP	SMAD1	11	LEMD1
UP	GPC6	8	hsa-mir-1306-5p	UP	ZIC3	11	TIMP4
UP	IRX5	8	hsa-mir-522-5p	UP	FOXO1	10	DAND5
UP	KRT20	8	hsa-mir-429	UP	IRF1	10	RHOJ
UP	LRFN5	8	hsa-mir-369-3p	UP	TBX5	9	FBXO10
UP	TM4SF5	8	hsa-mir-203a-3p	UP	DACH1	9	NKAIN4
UP	ANP32D	8	hsa-mir-375	UP	RCOR2	9	SOX18
UP	BCO1	8	hsa-mir-137	UP	MYB	8	CCDC158
UP	SPINK4	8	hsa-mir-374a-5p	UP	HOXC9	8	RGS4
UP	SCN3B	8	hsa-mir-10b-5p	UP	PDX1	8	SYT4
UP	SOX18	8	hsa-mir-373-3p	UP	SRF	7	ABRA
UP	MTRNR2L6	8	hsa-mir-30b-5p	UP	BCL3	7	ADAM7
UP	EMILIN3	8	hsa-mir-1301-3p	UP	HOXB4	7	BPIFC
UP	ECM2	8	hsa-mir-548e-3p	UP	SREBF1	7	C8ORF34
UP	DKK2	8	hsa-mir-1260b	UP	ZNF274	7	CWH43
UP	CAPN9	8	hsa-mir-20b-5p	UP	VDR	7	ELTD1
UP	CRYAA	8	hsa-mir-185-3p	UP	KLF5	7	GSX2
UP	OSR1	8	hsa-mir-185-3p	UP	PADI4	7	KCNT2
UP	SHISA8	7	hsa-mir-376a-5p	UP	GBX2	7	NELL1
UP	SPINK13	7	hsa-mir-16-5p	UP	KLF2	7	SOX2
UP	LEMD1	7	hsa-mir-147a	UP	NFIB	6	BTBD16
UP	NPBWR1	7	hsa-mir-373-3p	UP	NOTCH1	6	CAPSL
UP	OR51E2	7	hsa-mir-616-5p	UP	PRDM5	6	CYP2W1
UP	PDPN	7	hsa-mir-520c-3p	UP	IKZF1	6	EMILIN3
UP	GPR6	7	hsa-mir-93-5p	UP	MECOM	6	MICU3
UP	ASCL1	7	hsa-mir-302a-3p	UP	ESR2	6	NPNT
UP	BTBD16	7	hsa-mir-671-5p	UP	RARG	6	THRSP
UP	PCDHB2	7	hsa-mir-1343-3p	UP	XRN2	5	C2ORF73
UP	CELF4	7	hsa-mir-4511	UP	ASXL1	5	FNDC8
UP	BARX2	7	hsa-mir-638	UP	TBP	5	FSD2
UP	CA10	7	hsa-mir-219a-2-3p	UP	THRA	5	GNAT1
UP	PLA2G5	7	hsa-mir-122-5p	UP	CHD1	5	MIF
UP	HOXD8	7	hsa-mir-340-5p	UP	CEBPA	5	SERPIND1
UP	INHA	7	hsa-mir-346	UP	HIF1A	5	UPK1B
UP	ALPI	7	hsa-mir-148b-3p	UP	HOXD13	4	ANGPT4
UP	PLN	6	hsa-mir-205-5p	UP	NUCKS1	4	CRP
UP	SLC51B	6	hsa-mir-194-5p	UP	PHF8	4	TEAD4
UP	WT1	6	hsa-mir-766-5p	UP	ZNF652	3	CNTN2
UP	DAND5	6	hsa-mir-122-5p	UP	DCP1A	3	GULP1
UP	REG1B	6	hsa-mir-224-5p	UP	TAF7L	3	IRX5
UP	RNF180	6	hsa-mir-103a-2-5p	UP	KDM5A	3	MRPL12
UP	MEGF10	6	hsa-mir-369-3p	UP	TFEB	3	SPINK4
UP	DMRTA2	6	hsa-mir-588	UP	SALL1	2	GRIA4
UP	KCNH5	6	hsa-mir-4786-3p	UP	ETS1	2	IZUMO1
UP	ACSBG2	6	hsa-mir-940	UP	BP1	2	SELE
UP	IL17B	6	hsa-mir-23b-3p	UP	ZNF322	2	TRHDE
UP	AMBP	6	hsa-mir-224-3p	UP	GLI1	2	ZSWIM2
UP	HECW1	6	hsa-mir-4701-3p	UP	MYBL1	1	CSHL1
UP	AS3MT	6	hsa-mir-2277-3p	UP	KDM6A	1	DRD2
UP	EFS	6	hsa-mir-210-3p	UP	ETS2	1	GPC6
UP	COL6A5	6	hsa-mir-590-3p	UP	CHD7	1	INHA
UP	DNAH3	6	hsa-mir-605-5p	UP	AP1S2	1	IZUMO1
UP	ADCYAP1R1	6	hsa-mir-365b-3p	UP	NR1H3	1	MRPL12
UP	NACA2	6	hsa-mir-500a-3p	UP	NR4A2	1	MYT1L
UP	CCDC144A	6	hsa-mir-4789-5p	UP	STAT6	1	PLN
UP	SI	6	hsa-mir-203a-3p	UP	HCFC1	1	SCG3
UP	ELAVL3	6	hsa-mir-151a-5p	UP	PRDM16	1	TRHDE
UP	PDF	6	hsa-mir-20a-5p	Down	SPI1	316	STAT3
UP	ZNF728	5	hsa-mir-550a-3p	Down	RUNX1	235	SETDB1
UP	MYH4	5	hsa-mir-99b-5p	Down	MYC	235	TP53
UP	CCDC169	5	hsa-mir-125b-2-3p	Down	EGR1	227	TLE3
UP	SLCO6A1	5	hsa-mir-1180-3p	Down	FLI1	222	IRF1
UP	TUBA3C	5	hsa-mir-3619-5p	Down	SOX2	216	VDR
UP	SLC22A25	5	hsa-mir-200b-3p	Down	HNF4A	210	BCL3
UP	HSD11B2	5	hsa-mir-769-3p	Down	NANOG	194	KIAA0247
UP	MRGPRF	5	hsa-mir-2467-5p	Down	MITF	180	ABTB2
UP	HSPB2	5	hsa-mir-100-5p	Down	POU5F1	179	CNOT3
UP	MYRIP	5	hsa-mir-4708-3p	Down	TP63	175	MEF2D
UP	LMO1	5	hsa-mir-1224-3p	Down	CREM	173	SEC14L1
UP	GPR15	5	hsa-mir-138-5p	Down	E2F1	167	STAT6
UP	CAPSL	5	hsa-mir-155-5p	Down	GATA2	161	ARID1A
UP	HBZ	5	hsa-mir-146a-5p	Down	CREB1	160	EHBP1L1
UP	IGSF21	5	hsa-mir-182-5p	Down	GATA1	156	TNS3
UP	NT5C1A	5	hsa-mir-373-3p	Down	TAL1	139	TRAPPC9
UP	PLA2G10	5	hsa-mir-34a-5p	Down	KLF4	138	PRCC
UP	CST6	5	hsa-mir-522-5p	Down	AR	138	SLC9A8
UP	AVP	5	hsa-mir-302a-3p	Down	REST	134	PADI4
UP	CCL19	5	hsa-mir-148b-3p	Down	FOXP1	132	BMF
UP	PHF21B	5	hsa-mir-4482-5p	Down	TFAP2C	124	PLEKHG3
UP	RHBDL2	5	hsa-mir-210-3p	Down	TET1	124	STK40
UP	DCC	5	hsa-mir-542-3p	Down	PPARG	124	TRIM25
UP	CBLC	5	hsa-mir-195-5p	Down	SOX9	120	DLGAP4
UP	CDKL2	5	hsa-mir-135a-5p	Down	SIN3B	119	LASP1
UP	ISY1-RAB43	4	hsa-mir-378 g	Down	SRY	107	MIDN
UP	BTBD18	4	hsa-mir-941	Down	MECOM	102	CD97
UP	ANKRD18B	4	hsa-mir-374a-5p	Down	SUZ12	102	PLXNA2
UP	C17orf102	4	hsa-mir-376c-3p	Down	FOXA2	101	CLPB
UP	FSIP2	4	hsa-mir-22-5p	Down	E2F4	101	POLR2A
UP	PERM1	4	hsa-mir-345-5p	Down	TCF3	101	SLC1A4
UP	CYP4X1	4	hsa-mir-210-3p	Down	TCF4	97	STK10
UP	KPNA7	4	hsa-mir-191-5p	Down	ERG	96	ARRB1
UP	OR51E1	4	hsa-mir-145-5p	Down	PPARD	96	PVRL1
UP	ANKRD30B	4	hsa-mir-27b-3p	Down	TRIM28	92	ADAR
UP	LRRC3B	4	hsa-let-7 g-3p	Down	SMAD4	91	DAP
UP	C2orf73	4	hsa-mir-605-5p	Down	MYCN	91	ZNFX1
UP	MAP3K19	4	hsa-mir-520f-3p	Down	ZFX	89	TAF8
UP	GLYATL1	4	hsa-mir-1343-3p	Down	CCND1	88	KDM6B
UP	NELL1	4	hsa-mir-20a-5p	Down	KDM5B	84	WASF2
UP	SLCO1C1	4	hsa-mir-128-3p	Down	ASH2L	82	ATP6V0A1
UP	IL21	4	hsa-mir-372-3p	Down	YY1	82	HIVEP3
UP	ZSCAN10	4	hsa-mir-302a-3p	Down	EOMES	81	RERE
UP	NEUROG3	4	hsa-mir-373-3p	Down	ZNF281	80	TBC1D13
UP	ASPA	4	hsa-mir-1271-5p	Down	ATF3	79	WBP2
UP	WFDC1	4	hsa-mir-941	Down	TBX5	79	WRAP53
UP	SLC10A1	4	hsa-mir-449a	Down	DMRT1	75	F11R
UP	SLC16A8	4	hsa-mir-550a-3-5p	Down	SCLY	74	PISD
UP	PDE6C	4	hsa-mir-18a-3p	Down	FOXO3	73	ADAM19
UP	ABCC12	4	hsa-mir-3611	Down	LMO2	73	SEC24C
UP	G6PC	4	hsa-mir-33b-5p	Down	TFCP2L1	72	FAM168A
UP	FAM131C	4	hsa-mir-1343-3p	Down	HOXB4	72	GBF1
UP	SMTNL2	4	hsa-mir-373-3p	Down	MYBL2	71	SEMA4B
UP	NLRP14	4	hsa-mir-941	Down	SALL4	70	BAG6
UP	PCDHB10	4	hsa-mir-374a-5p	Down	TCF7	69	DOPEY2
UP	ANGPT4	4	hsa-mir-107	Down	TFAP2A	69	WWP2
UP	REN	4	hsa-mir-200c-3p	Down	RCOR3	68	KSR1
UP	APOH	4	hsa-mir-136-5p	Down	SMAD3	67	SIPA1L1
UP	EN1	4	hsa-mir-381-3p	Down	ESR1	66	PPCDC
UP	CCL17	4	hsa-mir-148b-3p	Down	RUNX2	66	PREX1
UP	C14orf39	4	hsa-mir-330-5p	Down	SMARCA4	65	GATAD2B
UP	HFM1	4	hsa-mir-490-5p	Down	RAD21	64	MKL1
UP	OTX2	4	hsa-mir-181a-5p	Down	GATA4	63	GABARAP
UP	PDE11A	4	hsa-mir-1343-3p	Down	GFI1B	63	NDE1
UP	B3GALT1	4	hsa-mir-4496	Down	EP300	62	MAP7D1
UP	NR0B2	4	hsa-mir-24-3p	Down	PRDM14	62	TNFRSF21
UP	CTAGE6	3	hsa-mir-99b-5p	Down	MEIS1	61	CSNK2B
UP	PMF1-BGLAP	3	hsa-let-7b-5p	Down	NR0B1	61	IP6K1
UP	C19orf81	3	hsa-mir-130b-5p	Down	PBX1	61	LY6G5B
UP	C1orf141	3	hsa-mir-3685	Down	CUX1	60	ATP6V0D1
UP	LHFPL3	3	hsa-mir-30d-5p	Down	YAP1	59	CBX7
UP	OR51B4	3	hsa-mir-214-3p	Down	KLF1	58	GAB2
UP	KCNIP1	3	hsa-mir-10b-5p	Down	TEAD4	58	STIM1
UP	DPP10	3	hsa-mir-1301-3p	Down	CEBPB	57	AGER
UP	FAM71D	3	hsa-mir-301a-5p	Down	KDM5A	57	SYVN1
UP	GYPA	3	hsa-mir-31-5p	Down	CTCF	57	YLPM1
UP	SERPINA9	3	hsa-mir-212-3p	Down	SRF	56	ADD1
UP	KLK4	3	hsa-mir-429	Down	WT1	56	PNKD
UP	SERPINB12	3	hsa-mir-598-3p	Down	STAT4	55	GNAI2
UP	TBX20	3	hsa-mir-34c-5p	Down	MTF2	54	MAPKAPK2
UP	TCHH	3	hsa-mir-147a	Down	XRN2	52	SLC9A1
UP	ZSCAN1	3	hsa-mir-155-5p	Down	HOXC9	50	TRIM41
UP	DUSP15	3	hsa-mir-146a-5p	Down	RBPJ	49	ARHGAP17
UP	ZNF214	3	hsa-mir-224-5p	Down	FOXP2	49	DGAT2
UP	TRIM55	3	hsa-mir-429	Down	EZH2	49	SIRPA
UP	PADI3	3	hsa-mir-129-2-3p	Down	IRF8	49	XPO6
UP	DDC	3	hsa-mir-517a-3p	Down	CTNNB1	48	TLN1
UP	MAS1	3	hsa-mir-182-5p	Down	TTF2	45	FAM53C
UP	KIF25	3	hsa-mir-101-3p	Down	SMAD2	45	MFN2
UP	CTCFL	3	hsa-mir-100-5p	Down	DACH1	45	POLR1A
UP	KCNJ13	3	hsa-mir-9-5p	Down	SOX17	45	SESN2
UP	REG1A	3	hsa-mir-1270	Down	FOXP3	44	ACP2
UP	UPK1B	3	hsa-mir-29c-3p	Down	RELA	43	DTX4
UP	RFX4	3	hsa-mir-532-3p	Down	ESRRB	41	BAZ2A
UP	NKAIN4	3	hsa-mir-1343-3p	Down	ZNF217	41	NF2
UP	CLUL1	3	hsa-mir-941	Down	MYB	41	RAB37
UP	ADAM7	3	hsa-mir-302a-3p	Down	ELK1	41	SNX11
UP	C8B	3	hsa-mir-373-3p	Down	TBP	40	AGPAT1
UP	CACNA1G	3	hsa-mir-1343-3p	Down	ELF1	40	ITGA5
UP	TSPYL6	3	hsa-mir-423-5p	Down	NR3C1	39	DYSF
UP	KCNK4	3	hsa-mir-27a-3p	Down	ETS1	39	KRTCAP2
UP	WIF1	3	hsa-mir-200b-3p	Down	THAP11	38	DENND1A
UP	RPE65	3	hsa-mir-103a-3p	Down	EWSR1	37	KIAA0556
UP	FABP6	3	hsa-mir-214-3p	Down	BMI1	37	MAML3
UP	ZPLD1	3	hsa-mir-148b-3p	Down	SIN3A	37	NDST1
UP	COX8C	3	hsa-mir-132-3p	Down	RNF2	36	RTN1
UP	ALDOB	3	hsa-mir-4690-5p	Down	SOX11	35	C14ORF159
UP	MAS1L	3	hsa-mir-7-5p	Down	ASXL1	35	PACS1
UP	FOLH1	3	hsa-mir-100-5p	Down	CRX	34	SCAP
UP	CYP2F1	3	hsa-mir-34b-5p	Down	JUN	33	CTDSP2
UP	RAD21L1	2	hsa-mir-103a-3p	Down	ZFP42	33	MARK2
UP	CFHR1	2	hsa-mir-671-5p	Down	MEF2A	33	TOP3A
UP	EXOC3L4	2	hsa-mir-133a-3p	Down	PHF8	32	GSK3A
UP	C10orf113	2	hsa-mir-941	Down	TFEB	32	OGDH
UP	DPPA5	2	hsa-mir-182-5p	Down	JARID2	32	RAP1GAP2
UP	LPA	2	hsa-mir-147a	Down	SREBF2	31	SIK3
UP	RD3	2	hsa-mir-744-5p	Down	STAT5A	29	MAFF
UP	NRAP	2	hsa-mir-129-2-3p	Down	DNAJC2	28	C15ORF39
UP	PRELP	2	hsa-mir-382-5p	Down	NFE2L2	28	CHST15
UP	C2orf80	2	hsa-mir-124-3p	Down	TBX3	27	CIITA
UP	TMEM72	2	hsa-mir-103a-3p	Down	PDX1	27	FAM214B
UP	NPAP1	2	hsa-mir-26a-5p	Down	CHD1	27	INTS3
UP	FREM3	2	hsa-mir-3928-3p	Down	NR1I2	27	SETDB1
UP	LIPF	2	hsa-mir-27a-3p	Down	HSF1	26	DNMBP
UP	HEPHL1	2	hsa-mir-15a-3p	Down	OLIG2	26	RNF24
UP	OR52N5	2	hsa-mir-34b-5p	Down	LYL1	26	ZNF592
UP	GSX2	2	hsa-mir-155-5p	Down	CDX2	25	CDK5RAP3
UP	WFDC5	2	hsa-mir-191-5p	Down	NR1H3	25	SMG5
UP	TRHR	2	hsa-mir-182-5p	Down	BACH1	23	JAK3
UP	OR1L3	2	hsa-mir-200a-3p	Down	GATA3	23	RNPEP
UP	OR2K2	2	hsa-mir-146a-5p	Down	PRDM5	23	SMURF1
UP	KRT78	2	hsa-mir-126-3p	Down	ELF5	22	CRTC2
UP	A2ML1	2	hsa-mir-449a	Down	TAF7L	22	PRKACA
UP	CCDC158	2	hsa-mir-375	Down	NACC1	22	SLC44A2
UP	CCDC140	2	hsa-mir-520f-3p	Down	NUCKS1	22	VAMP2
UP	CCDC27	2	hsa-mir-942-5p	Down	ESR2	21	EPS15L1
UP	LRRC71	2	hsa-mir-23b-3p	Down	DCP1A	21	MICALL1
UP	FUT6	2	hsa-mir-330-3p	Down	AP1S2	21	TRPC4AP
UP	CNTNAP4	2	hsa-mir-15a-5p	Down	TCF7L2	20	HIP1
UP	UGT2A3	2	hsa-mir-210-3p	Down	PAX3	20	SPN
UP	CRP	2	hsa-mir-378 g	Down	ARNT	20	TMEM229B
UP	CDH15	2	hsa-mir-1343-3p	Down	RCOR1	20	ZNF692
UP	CST8	2	hsa-mir-27a-3p	Down	NFIB	19	RELL1
UP	MYH7	2	hsa-mir-155-5p	Down	STAT1	19	TK2
UP	CYP2W1	2	hsa-mir-124-3p	Down	PAX6	18	CRISPLD2
UP	CAMK1G	2	hsa-mir-3661	Down	CEBPA	18	VPS37C
UP	TMPRSS12	2	hsa-mir-26a-5p	Down	DROSHA	14	ABCG1
UP	RBM46	2	hsa-mir-27a-3p	Down	EED	14	CAMKK1
UP	MYOT	2	hsa-mir-26a-5p	Down	CLOCK	14	FLCN
UP	LRAT	2	hsa-mir-375	Down	PHC1	14	KIAA0319L
UP	KRT38	2	hsa-mir-16-5p	Down	SREBF1	14	QSOX1
UP	FFAR1	2	hsa-mir-146a-5p	Down	AHR	14	SYK
UP	LHCGR	2	hsa-mir-7-5p	Down	SMAD1	13	PIK3R5
UP	CSNK1A1L	2	hsa-mir-374a-5p	Down	HCFC1	13	PPP1R12
UP	ZSCAN4	2	hsa-mir-122-5p	Down	THRA	13	RNF31
UP	ABCB5	2	hsa-mir-1-3p	Down	ZIC3	13	SMARCD1
UP	BOLA2B	2	hsa-mir-603	Down	IKZF1	12	PHC2
UP	S100A7A	2	hsa-mir-26b-5p	Down	FOXO1	11	TNFRSF1B
UP	IRGC	2	hsa-mir-205-5p	Down	NOTCH1	10	ZNF324
UP	P2RX2	2	hsa-mir-146a-5p	Down	HIF1A	9	DAPK2
UP	MYOG	2	hsa-mir-200b-3p	Down	RARG	9	MBD6
UP	GHSR	2	hsa-mir-212-3p	Down	RCOR2	9	NSD1
UP	CNTN2	2	hsa-mir-1908-5p	Down	KDM6A	8	GALNS
UP	RBAK-RBAKDN	1	hsa-mir-378a-5p	Down	FOXM1	8	PI4K2A
UP	HSPE1-MOB4	1	hsa-mir-1-3p	Down	ETS2	8	ZSWIM1
UP	KRTAP10–5	1	hsa-mir-20a-5p	Down	HTT	7	SCAMP5
UP	AMY1A	1	hsa-mir-335-5p	Down	POU3F2	6	GPSM3
UP	MKRN2OS	1	hsa-mir-766-3p	Down	TCF21	5	GYS1
UP	RFPL4A	1	hsa-mir-101-3p	Down	CEBPD	5	POPDC2
UP	TDRD15	1	hsa-mir-455-5p	Down	ZNF274	5	SUPT5H
UP	GCGR	1	hsa-mir-101-3p	Down	MYBL1	4	CNTROB
UP	CSHL1	1	hsa-mir-146a-5p	Down	CDKN2AIP	4	MAP3K3
UP	KRT39	1	hsa-mir-1-3p	Down	CHD7	4	SNX27
UP	FSD2	1	hsa-mir-5682	Down	SALL1	3	DIAPH1
UP	TMPRSS11B	1	hsa-mir-27a-3p	Down	PRDM16	3	GALNT6
UP	SPATA8	1	hsa-mir-182-5p	Down	ZNF322	3	KDELR1
UP	BPIFC	1	hsa-mir-27a-3p	Down	GBX2	3	PLXNA4
UP	MRGPRE	1	hsa-mir-31-5p	Down	NR4A2	3	PPP1R10
UP	TMIGD1	1	hsa-mir-124-3p	Down	HOXD13	3	RAF1
UP	IZUMO1	1	hsa-mir-146a-5p	Down	ZNF652	3	U2AF1L4
UP	CCL13	1	hsa-mir-27a-3p	Down	ZNF263	1	PRSS21
UP	GPR62	1	hsa-mir-520f-3p	Down	KLF2	1	PXN
UP	RPRML	1	hsa-mir-146a-5p	Down	KLF5	1	PXN
UP	ABRA	1	hsa-mir-520f-3p	Down	E2F7	1	ZNF687
UP	LIPM	1	hsa-mir-188-3p	Down	BCL11B	1	SP110
UP	EFCAB3	1	hsa-mir-135b-5p				
UP	PRL	1	hsa-mir-27a-3p				
UP	UTF1	1	hsa-mir-302a-3p				
UP	LKAAEAR1	1	hsa-mir-146a-5p				
UP	FRMPD2	1	hsa-mir-3929				
UP	TM4SF20	1	hsa-mir-128-3p				
UP	TBX10	1	hsa-mir-429				
UP	MS4A5	1	hsa-mir-146a-5p				
UP	MOGAT2	1	hsa-mir-210-3p				
UP	ZSWIM2	1	hsa-mir-27a-3p				
UP	MEIOB	1	hsa-mir-302a-3p				
UP	TMPRSS15	1	hsa-mir-34b-5p				
UP	THRSP	1	hsa-mir-27a-3p				
UP	TMC2	1	hsa-mir-27a-3p				
UP	DRD2	1	hsa-mir-101-3p				
UP	SPATA16	1	hsa-mir-27a-3p				
UP	THSD7B	1	hsa-mir-129-2-3p				
UP	CELA2A	1	hsa-mir-450b-5p				
UP	EGR4	1	hsa-mir-129-2-3p				
UP	MRO	1	hsa-mir-27a-3p				
UP	NPBWR2	1	hsa-mir-146a-5				
UP	PRLHR	1	hsa-mir-26a-5p				
UP	OPRM1	1	hsa-mir-590-3p				
UP	CRX	1	hsa-mir-766-3p				
UP	GGTLC2	1	hsa-mir-107				
UP	PLA2G3	1	hsa-mir-27a-3p				
UP	NXPE1	1	hsa-mir-34a-5p				
UP	RDH8	1	hsa-mir-27a-3p				
UP	NPFFR2	1	hsa-mir-26a-5p				
UP	MYH13	1	hsa-mir-26a-5p				
UP	RASA4B	1	hsa-mir-335-5p				
UP	GOLGA6L9	1	hsa-mir-335-5p				
UP	KRTAP10–6	1	hsa-mir-335-5p				
UP	TAC4	1	hsa-mir-335-5p				
UP	OR3A1	1	hsa-mir-335-5p				
UP	NPY	1	hsa-mir-335-5p				
UP	HSD3B2	1	hsa-mir-335-5p				
UP	UGT2A1	1	hsa-mir-26b-5p				
Down	MIDN	182	hsa-mir-4705				
Down	NSD1	163	hsa-mir-1271-3p				
Down	ZNF264	153	hsa-mir-4517				
Down	BAZ2A	153	hsa-mir-137				
Down	ZNFX1	141	hsa-mir-150-3p				
Down	ARID1A	138	hsa-mir-3929				
Down	GATAD2B	126	hsa-mir-4803				
Down	SOGA1	123	hsa-mir-1304-3p				
Down	PLAGL2	116	hsa-mir-196a-5p				
Down	PRR14L	113	hsa-mir-3691-5p				
Down	POLR2A	111	hsa-mir-3187-3p				
Down	SESN2	110	hsa-mir-1825				
Down	ARF3	109	hsa-mir-2278				
Down	PSAP	108	hsa-mir-7-1-3p				
Down	ATXN1L	107	hsa-mir-1915-5p				
Down	PRRC2A	105	hsa-mir-760				
Down	ADAR	105	hsa-mir-3157-3p				
Down	MEF2D	104	hsa-mir-642b-3p				
Down	TLN1	102	hsa-mir-148b-3p				
Down	USP22	100	hsa-mir-4511				
Down	WASF2	94	hsa-mir-34c-3p				
Down	KDM6B	90	hsa-mir-519b-3p				
Down	MTF1	90	hsa-mir-516b-5p				
Down	STAT3	85	hsa-mir-544a				
Down	DIAPH1	85	hsa-mir-518c-5p				
Down	STK35	82	hsa-mir-3177-5p				
Down	DENND4B	81	hsa-mir-1304-5p				
Down	TMEM127	80	hsa-mir-1277-5p				
Down	GLYR1	80	hsa-mir-188-5p				
Down	PPP1R10	80	hsa-mir-296-5p				
Down	MAVS	79	hsa-mir-490-3p				
Down	RAB11FIP1	79	hsa-mir-199a-3p				
Down	MBD6	78	hsa-mir-1913				
Down	DSP	77	hsa-mir-217				
Down	IGF2R	77	hsa-mir-519d-3p				
Down	CHD8	77	hsa-mir-520a-3p				
Down	ASF1B	75	hsa-mir-196b-3p				
Down	HYOU1	74	hsa-mir-18b-3p				
Down	SCAMP2	74	hsa-mir-1287-5p				
Down	LASP1	73	hsa-mir-106b-3p				
Down	RAD54L2	73	hsa-mir-141-5p				
Down	IRF1	73	hsa-mir-1296-5p				
Down	SEC24C	73	hsa-mir-181c-5p				
Down	CPSF7	69	hsa-mir-3115				
Down	TNFRSF21	68	hsa-mir-129-1-3p				
Down	XPO6	66	hsa-mir-139-5p				
Down	SMARCD1	65	hsa-mir-10a-5p				
Down	TRIM25	65	hsa-mir-19a-5p				
Down	GBF1	63	hsa-mir-148a-3p				
Down	TBC1D13	63	hsa-mir-301b-3p				
Down	MAP1A	62	hsa-let-7f-2-3p				
Down	NCOA6	61	hsa-mir-23a-3p				
Down	CTDSP2	60	hsa-mir-21-5p				
Down	FAM168A	59	hsa-mir-331-3p				
Down	LMBR1L	59	hsa-mir-30e-3p				
Down	TP53	58	hsa-mir-1246				
Down	GNAI2	58	hsa-mir-5009-5p				
Down	TAPBP	57	hsa-mir-566				
Down	SORT1	57	hsa-mir-1301-3p				
Down	MYADM	56	hsa-mir-506-3p				
Down	STK40	55	hsa-mir-4458				
Down	NF2	54	hsa-mir-188-3p				
Down	KDELR1	54	hsa-mir-196a-5p				
Down	MAPKAPK2	54	hsa-mir-1976				
Down	MFN2	54	hsa-mir-1288-3p				
Down	RAF1	53	hsa-mir-744-3p				
Down	WBP2	53	hsa-mir-7-1-3p				
Down	RNF24	53	hsa-mir-512-3p				
Down	TLE3	53	hsa-mir-744-5p				
Down	TRIM41	53	hsa-mir-939-5p				
Down	YLPM1	52	hsa-mir-99b-3p				
Down	SMURF1	52	hsa-mir-548d-3p				
Down	SLC25A44	52	hsa-mir-5582-3p				
Down	F11R	52	hsa-mir-1299				
Down	C15orf39	51	hsa-mir-649				
Down	SIPA1L1	51	hsa-mir-922				
Down	CRTC2	51	hsa-mir-4690-5p				
Down	PI4KB	50	hsa-mir-4487				
Down	ARHGAP1	49	hsa-mir-3188				
Down	NDST1	49	hsa-mir-551a				
Down	LMTK2	49	hsa-mir-548d-5p				
Down	SNX27	49	hsa-mir-624-3p				
Down	MAP7D1	48	hsa-mir-5094				
Down	IP6K1	48	hsa-mir-107				
Down	ZDHHC18	48	hsa-mir-708-5p				
Down	VAT1	48	hsa-mir-573				
Down	CANT1	48	hsa-mir-615-3p				
Down	RERE	48	hsa-mir-1914-3p				
Down	MTMR3	47	hsa-mir-378d				
Down	PIGS	47	hsa-mir-628-5p				
Down	PHC2	47	hsa-mir-765				
Down	SEC14L1	47	hsa-mir-769-5p				
Down	POLR1A	46	hsa-mir-1281				
Down	MAPRE3	46	hsa-mir-3180-3p				
Down	ITGA5	46	hsa-mir-876-3p				
Down	PACS1	46	hsa-mir-30c-1-3p				
Down	ZNF445	46	hsa-mir-548j-5p				
Down	GPR107	45	hsa-mir-708-5p				
Down	PREX1	45	hsa-mir-608				
Down	ZNF592	45	hsa-mir-760				
Down	PBX2	44	hsa-mir-548ar-3p				
Down	SYVN1	44	hsa-let-7f-1-3p				
Down	SCAMP5	43	hsa-mir-618				
Down	H6PD	43	hsa-mir-1285-5p				
Down	TP53INP2	43	hsa-mir-365a-3p				
Down	PISD	42	hsa-mir-664a-5p				
Down	BAG6	42	hsa-mir-450a-1-3p				
Down	ADD1	42	hsa-mir-18b-5p				
Down	MAP3K3	42	hsa-mir-4802-3p				
Down	TBC1D10B	41	hsa-mir-301a-3p				
Down	RNF122	40	hsa-mir-455-5p				
Down	SLC9A1	39	hsa-let-7 g-3p				
Down	UBE2C	39	hsa-mir-4664-5p				
Down	BMF	39	hsa-mir-1910–5p				
Down	PLEKHG3	39	hsa-mir-3689f				
Down	TMEM214	39	hsa-mir-1468-5p				
Down	PLBD2	39	hsa-mir-377-3p				
Down	FAM160A1	39	hsa-mir-616-5p				
Down	EGLN2	38	hsa-mir-3687				
Down	ZNF385A	38	hsa-mir-2114-5p				
Down	WIPF2	38	hsa-mir-181d-5p				
Down	EXTL3	38	hsa-mir-30d-3p				
Down	VAMP2	38	hsa-mir-135a-5p				
Down	PVR	37	hsa-mir-200c-3p				
Down	SLC1A4	37	hsa-mir-3065-3p				
Down	GPR157	37	hsa-mir-520a-3p				
Down	TNS3	37	hsa-mir-543				
Down	AGPAT1	37	hsa-mir-873-5p				
Down	ARHGAP17	36	hsa-mir-199b-5p				
Down	SLC48A1	36	hsa-mir-320d				
Down	CPT2	36	hsa-mir-634				
Down	FLCN	36	hsa-mir-33b-5p				
Down	PXN	36	hsa-mir-421				
Down	SLC44A2	36	hsa-mir-320a				
Down	SMG5	36	hsa-mir-501-3p				
Down	SLC35F6	35	hsa-mir-29a-3p				
Down	NDE1	35	hsa-mir-629-5p				
Down	CYTH2	35	hsa-mir-342-5p				
Down	KCTD2	35	hsa-mir-204-3p				
Down	MBOAT7	34	hsa-mir-522-5p				
Down	DLGAP4	34	hsa-mir-379-5p				
Down	TMBIM1	34	hsa-mir-3689e				
Down	MARK2	34	hsa-mir-548q				
Down	STAT2	33	hsa-mir-509-3-5p				
Down	SEMA4B	33	hsa-mir-766-5p				
Down	OGDH	33	hsa-mir-494-3p				
Down	CNNM4	33	hsa-mir-520c-3p				
Down	UBN1	33	hsa-mir-30b-3p				
Down	KIAA0319L	33	hsa-mir-4999-5p				
Down	HIP1	33	hsa-mir-302d-3p				
Down	TRPC4AP	33	hsa-mir-4487				
Down	PPP1R12B	32	hsa-mir-3127-5p				
Down	PNKD	32	hsa-mir-638				
Down	ABAT	32	hsa-mir-378a-3p				
Down	CNOT3	32	hsa-mir-548n				
Down	MOB3A	31	hsa-mir-4443				
Down	LRP10	31	hsa-mir-922				
Down	C6orf89	31	hsa-mir-181c-5p				
Down	CYB561D1	31	hsa-mir-2114-5p				
Down	CBX7	30	hsa-mir-519a-3p				
Down	GBA2	30	hsa-mir-4518				
Down	PLXNA2	30	hsa-mir-3619-5p				
Down	TAGLN	30	hsa-mir-3200-3p				
Down	RGL2	30	hsa-mir-3943				
Down	METTL7A	30	hsa-mir-3613-3p				
Down	PIGO	30	hsa-mir-29c-3p				
Down	PROSER3	30	hsa-mir-328-3p				
Down	C6orf136	30	hsa-mir-532-3p				
Down	TRANK1	29	hsa-mir-671-5p				
Down	PRKACA	29	hsa-mir-3179				
Down	INTS3	29	hsa-mir-5699-3p				
Down	ARHGEF40	29	hsa-mir-548ax				
Down	GYS1	28	hsa-mir-4437				
Down	ACP2	28	hsa-mir-4521				
Down	QSOX1	28	hsa-mir-3180-3p				
Down	VPS37C	28	hsa-mir-15b-3p				
Down	SIK3	28	hsa-mir-137				
Down	MAFF	28	hsa-mir-423-5p				
Down	PRCC	28	hsa-mir-590-5p				
Down	GSK3A	27	hsa-mir-378d				
Down	DNMBP	27	hsa-mir-4733-5p				
Down	BCL3	27	hsa-mir-548y				
Down	LY6G5B	27	hsa-mir-497-5p				
Down	SLC38A7	26	hsa-mir-522-5p				
Down	FANCA	26	hsa-mir-2355-3p				
Down	ATP6V0D1	26	hsa-mir-582-3p				
Down	GABARAP	26	hsa-mir-888-3p				
Down	FAM53C	26	hsa-mir-548b-3p				
Down	PFKFB4	26	hsa-mir-550a-3-5p				
Down	SCAP	26	hsa-mir-365b-3p				
Down	ZNF692	26	hsa-mir-3127-3p				
Down	SIRPA	25	hsa-mir-3685				
Down	WDTC1	25	hsa-mir-361-3p				
Down	RAB3D	25	hsa-mir-744-3p				
Down	STIM1	24	hsa-mir-939-5p				
Down	PRKCD	24	hsa-mir-383-5p				
Down	STAT6	24	hsa-mir-520c-3p				
Down	SUPT5H	24	hsa-mir-188-3p				
Down	ZNF687	24	hsa-mir-579-3p				
Down	SH3BP2	24	hsa-mir-5008-5p				
Down	CASC3	24	hsa-mir-3934-5p				
Down	SF3A1	24	hsa-mir-583				
Down	RAPGEFL1	24	hsa-mir-320c				
Down	ABCC10	24	hsa-mir-31-5p				
Down	ADAM19	24	hsa-mir-29c-3p				
Down	EPHX1	23	hsa-mir-376a-5p				
Down	PAQR6	23	hsa-mir-424-5p				
Down	RAP1GAP2	23	hsa-mir-362-5p				
Down	KSR1	23	hsa-mir-150-3p				
Down	VPS53	23	hsa-mir-3662				
Down	HEATR6	23	hsa-mir-4803				
Down	BET1L	23	hsa-mir-500a-5p				
Down	PLEKHO2	22	hsa-mir-484				
Down	NUCB1	22	hsa-mir-335-5p				
Down	RIMS3	22	hsa-mir-455-3p				
Down	SNX11	22	hsa-mir-624-5p				
Down	SORBS3	22	hsa-mir-3619-5p				
Down	ZNF324	22	hsa-mir-361-3p				
Down	PRKD2	22	hsa-mir-22-3p				
Down	FAM219A	22	hsa-mir-4726-3p				
Down	CHST15	21	hsa-mir-29b-1-5p				
Down	MAML3	21	hsa-mir-221-5p				
Down	ARRB2	21	hsa-mir-181c-5p				
Down	CD4	21	hsa-mir-140-3p				
Down	HLA-DQA1	21	hsa-mir-330-3p				
Down	FAM214B	21	hsa-mir-3176				
Down	ADAT1	21	hsa-mir-191-5p				
Down	ARHGEF11	21	hsa-mir-744-5p				
Down	TTLL11	21	hsa-mir-302c-3p				
Down	CDKN2A	20	hsa-mir-24-1-5p				
Down	DUSP18	20	hsa-mir-106b-5p				
Down	SP110	20	hsa-mir-148a-5p				
Down	SPINT1	20	hsa-mir-452-5p				
Down	ATP6V0A1	19	hsa-mir-377-3p				
Down	SIDT2	19	hsa-mir-301a-5p				
Down	RXRB	19	hsa-mir-32-5p				
Down	APOL2	19	hsa-mir-103a-3p				
Down	USP19	19	hsa-mir-3200-3p				
Down	IQCE	19	hsa-mir-146b-5p				
Down	CHST14	19	hsa-mir-671-5p				
Down	TMEM179B	19	hsa-mir-631				
Down	CXCL16	19	hsa-mir-200c-3p				
Down	RNF19B	19	hsa-mir-99b-5p				
Down	NAPA	19	hsa-mir-103a-3p				
Down	ARRB1	18	hsa-mir-16-2-3p				
Down	CASP9	18	hsa-mir-1291				
Down	SMAP2	18	hsa-mir-629-3p				
Down	DENND1A	18	hsa-mir-361-5p				
Down	LRRC4	18	hsa-let-7c-5p				
Down	NLRX1	18	hsa-mir-1255a				
Down	TNFRSF1B	18	hsa-mir-125b-5p				
Down	CDK5RAP3	18	hsa-mir-342-3p				
Down	TK2	18	hsa-mir-302c-3p				
Down	NLRP1	18	hsa-mir-15b-5p				
Down	TAF8	18	hsa-mir-548 h-3p				
Down	GALNT6	18	hsa-mir-331-5p				
Down	RNPEP	18	hsa-mir-4664-5p				
Down	ORAI2	17	hsa-mir-5699-3p				
Down	PLEKHM1	17	hsa-mir-589-5p				
Down	PLXNA4	17	hsa-mir-17-3p				
Down	KIAA0513	17	hsa-mir-3065-3p				
Down	P2RX5	17	hsa-let-7f-5p				
Down	ZSWIM1	17	hsa-mir-196b-5p				
Down	MICALL1	17	hsa-mir-650				
Down	DENND3	17	hsa-mir-450b-5p				
Down	WWP2	17	hsa-mir-1202				
Down	LRRC20	16	hsa-let-7 g-5p				
Down	TEP1	16	hsa-mir-1281				
Down	TNFAIP2	16	hsa-mir-522-5p				
Down	HIVEP3	16	hsa-mir-29b-3p				
Down	DAP	16	hsa-mir-339-5p				
Down	HSH2D	16	hsa-mir-449a				
Down	CDIP1	16	hsa-let-7i-5p				
Down	MAPK13	16	hsa-mir-520 h				
Down	KCTD21	15	hsa-mir-1910–5p				
Down	NAGK	15	hsa-mir-92a-1-5p				
Down	DTX4	15	hsa-mir-561-5p				
Down	GAB2	15	hsa-mir-378 g				
Down	ABCG1	15	hsa-mir-588				
Down	CCNJL	15	hsa-let-7a-5p				
Down	DYSF	15	hsa-mir-520c-3p				
Down	RTN1	15	hsa-mir-9-5p				
Down	RELL1	15	hsa-mir-320b				
Down	ARHGAP30	15	hsa-mir-30e-5p				
Down	SETDB1	14	hsa-mir-3928-3p				
Down	BSCL2	14	hsa-mir-106a-5p				
Down	EPS15L1	14	hsa-mir-4448				
Down	MAST3	14	hsa-mir-518a-3p				
Down	APOL1	14	hsa-mir-133a-3p				
Down	FHOD1	14	hsa-mir-199a-5p				
Down	VARS2	14	hsa-mir-1260a				
Down	SLC9A8	14	hsa-mir-1468-5p				
Down	SKIV2L	14	hsa-mir-939-5p				
Down	C17orf49	13	hsa-mir-4804-5p				
Down	INPP5B	13	hsa-mir-301a-5p				
Down	CLN3	13	hsa-mir-29b-2-5p				
Down	TPCN2	13	hsa-mir-532-3p				
Down	NDRG2	13	hsa-mir-2355-5p				
Down	IL17RA	13	hsa-mir-769-3p				
Down	ABTB2	13	hsa-mir-3685				
Down	TMEM63B	13	hsa-mir-2110				
Down	ARHGAP31	13	hsa-mir-5690				
Down	STK10	13	hsa-mir-138-5p				
Down	RNF31	13	hsa-mir-103a-2-5p				
Down	GTPBP1	13	hsa-mir-548an				
Down	SLC15A3	13	hsa-mir-373-3p				
Down	VDR	13	hsa-mir-449c-5p				
Down	DAAM2	13	hsa-mir-940				
Down	FIZ1	12	hsa-mir-5100				
Down	TMEM106A	12	hsa-mir-933				
Down	POMZP3	12	hsa-mir-203a-3p				
Down	CADM4	12	hsa-mir-4726-3p				
Down	TRAPPC9	12	hsa-mir-330-3p				
Down	ZNF530	12	hsa-mir-181b-5p				
Down	RAB36	11	hsa-mir-374a-5p				
Down	LGALS9	11	hsa-mir-133a-3p				
Down	PI4K2A	11	hsa-mir-346				
Down	ST6GALNAC2	11	hsa-mir-212-3p				
Down	AOC2	11	hsa-mir-3199				
Down	ARSG	11	hsa-mir-183-5p				
Down	SLC27A3	11	hsa-mir-503-5p				
Down	C7orf26	11	hsa-mir-320e				
Down	SLC16A5	11	hsa-mir-885-5p				
Down	RNF135	11	hsa-mir-217				
Down	TMEM229B	11	hsa-mir-2467-5p				
Down	XKR8	10	hsa-mir-125a-5p				
Down	CRISPLD2	10	hsa-mir-365b-5p				
Down	ARHGEF5	10	hsa-mir-302d-3p				
Down	PRSS21	10	hsa-let-7d-5p				
Down	JAK3	10	hsa-mir-221-3p				
Down	MARK4	10	hsa-mir-2277-3p				
Down	PRR16	10	hsa-let-7b-5p				
Down	ZNF70	10	hsa-mir-589-3p				
Down	TTLL3	10	hsa-mir-1179				
Down	ZBTB3	9	hsa-mir-29c-3p				
Down	EHBP1L1	9	hsa-mir-548a-3p				
Down	ZBTB22	9	hsa-mir-642a-5p				
Down	CHKB	9	hsa-mir-1229-3p				
Down	STK36	9	hsa-mir-5581-3p				
Down	ABTB1	9	hsa-mir-21-3p				
Down	PTPN18	9	hsa-mir-647				
Down	ARAP3	9	hsa-mir-616-5p				
Down	PPCDC	9	hsa-mir-500a-5p				
Down	PIK3R5	9	hsa-mir-3064-5p				
Down	ZNF417	9	hsa-mir-1271-5p				
Down	SLC16A13	9	hsa-mir-873-3p				
Down	TOP3A	9	hsa-mir-181a-2-3p				
Down	CSNK2B	9	hsa-mir-3619-5p				
Down	INPP5D	8	hsa-mir-589-3p				
Down	THBS3	8	hsa-mir-933				
Down	ZNF784	8	hsa-mir-432-3p				
Down	SYK	8	hsa-mir-3158-3p				
Down	TRIM62	8	hsa-mir-522-5p				
Down	CLPB	8	hsa-mir-1180-3p				
Down	TMEM63C	8	hsa-mir-4999-5p				
Down	SEMA4A	8	hsa-mir-628-5p				
Down	GPSM3	8	hsa-mir-664b-5p				
Down	ALKBH6	8	hsa-mir-766-3p				
Down	SPN	7	hsa-let-7e-5p				
Down	CSF1R	7	hsa-mir-3065-3p				
Down	NICN1	7	hsa-mir-24-3p				
Down	SLC6A16	7	hsa-mir-4254				
Down	C2CD2L	7	hsa-mir-125a-5p				
Down	EPOR	7	hsa-mir-3611				
Down	CIITA	7	hsa-mir-142-3p				
Down	DGKG	7	hsa-mir-574-5p				
Down	PAPLN	7	hsa-mir-133a-3p				
Down	KCNIP2	7	hsa-mir-548e-3p				
Down	DPEP3	7	hsa-mir-30a-5p				
Down	CCDC17	7	hsa-mir-1908-5p				
Down	TTC4	7	hsa-mir-23b-3p				
Down	ADAP2	6	hsa-mir-26b-5p				
Down	RPGRIP1	6	hsa-mir-374a-5p				
Down	DGAT2	6	hsa-mir-218-5p				
Down	ASPRV1	6	hsa-mir-520c-3p				
Down	FLT3	6	hsa-mir-212-3p				
Down	SHISA4	6	hsa-mir-22-5p				
Down	RAB37	6	hsa-mir-214-3p				
Down	CAMKK1	6	hsa-mir-5008-5p				
Down	GMIP	6	hsa-mir-1303				
Down	REC8	6	hsa-mir-5680				
Down	PLD2	6	hsa-mir-200b-3p				
Down	GALNS	6	hsa-mir-874-3p				
Down	CNTROB	6	hsa-mir-1254				
Down	APOM	6	hsa-mir-10b-5p				
Down	KCTD11	6	hsa-mir-140-3p				
Down	ITGAX	5	hsa-mir-126-3p				
Down	OSCAR	5	hsa-mir-7-5p				
Down	PTAFR	5	hsa-mir-497-5p				
Down	DAPK2	5	hsa-mir-520c-3p				
Down	WDFY4	5	hsa-mir-338-3p				
Down	ATG16L2	5	hsa-mir-362-3p				
Down	C19orf54	5	hsa-mir-296-3p				
Down	SIGLEC9	5	hsa-mir-147a				
Down	AOC3	5	hsa-mir-103a-3p				
Down	U2AF1L4	5	hsa-mir-660-5p				
Down	RGL3	5	hsa-mir-676-3p				
Down	PHF7	4	hsa-mir-147a				
Down	WRAP53	4	hsa-mir-205-5p				
Down	P2RX1	4	hsa-mir-191-5p				
Down	PSPN	4	hsa-mir-335-5p				
Down	SIGLEC5	4	hsa-mir-941				
Down	POPDC2	4	hsa-mir-10b-5p				
Down	SH3D21	4	hsa-mir-615-5p				
Down	TIGD3	4	hsa-mir-548o-3p				
Down	KIAA0556	4	hsa-mir-101-3p				
Down	LAG3	4	hsa-mir-146a-5p				
Down	ARHGAP9	4	hsa-mir-372-3p				
Down	SMPD2	4	hsa-mir-27b-5p				
Down	ZMYND15	4	hsa-mir-194-5p				
Down	PLB1	4	hsa-mir-205-5p				
Down	NOMO2	4	hsa-mir-15a-3p				
Down	SULT1A2	4	hsa-mir-210-3p				
Down	MPEG1	4	hsa-mir-28-5p				
Down	SPIB	4	hsa-mir-200b-5p				
Down	EIF3CL	3	hsa-mir-194-5p				
Down	TNFSF12	3	hsa-mir-29c-3p				
Down	GTF2IRD2	3	hsa-mir-522-5p				
Down	OGFOD2	3	hsa-mir-3613-5p				
Down	C16orf54	3	hsa-mir-27a-3p				
Down	CLEC17A	3	hsa-mir-30c-1-3p				
Down	ITGAL	3	hsa-mir-4651				
Down	VNN3	3	hsa-mir-671-5p				
Down	TREML2	3	hsa-mir-214-3p				
Down	RIPK3	3	hsa-mir-212-3p				
Down	ADCY4	3	hsa-mir-1290				
Down	CPNE9	3	hsa-mir-301a-5p				
Down	KRTCAP2	3	hsa-mir-603				
Down	BEST1	3	hsa-mir-144-3p				
Down	ITGAM	3	hsa-mir-372-3p				
Down	RASGRP4	3	hsa-mir-34c-5p				
Down	HCG27	3	hsa-mir-941				
Down	APOBEC3D	3	hsa-mir-449b-5p				
Down	RABL2A	2	hsa-mir-1-3p				
Down	ITIH4	2	hsa-mir-101-3p				
Down	MMP25	2	hsa-mir-212-3p				
Down	PILRA	2	hsa-mir-16-5p				
Down	CSF3R	2	hsa-mir-941				
Down	SLC25A35	2	hsa-mir-1343-3p				
Down	C5AR2	2	hsa-mir-34a-5p				
Down	KCNMB1	2	hsa-mir-195-5p				
Down	NOXRED1	2	hsa-mir-520f-3p				
Down	NPIPB3	2	hsa-mir-154-5p				
Down	PDZD3	2	hsa-mir-210-3p				
Down	UBA7	2	hsa-mir-130a-3p				
Down	BLOC1S3	2	hsa-mir-34c-5p				
Down	NFAM1	2	hsa-mir-1293				
Down	C19orf84	2	hsa-mir-182-5p				
Down	AMY1B	1	hsa-mir-335-5p				
Down	PADI4	1	hsa-mir-335-5p				
Down	DRICH1	1	hsa-mir-335-5p				
Down	LCNL1	1	hsa-mir-335-5p				
Down	CEACAM4	1	hsa-mir-31-5p				
Down	GBGT1	1	hsa-mir-429				
Down	GHRL	1	hsa-mir-941				
Down	TMEM234	1	hsa-mir-1-3p				
Down	DPEP2	1	hsa-mir-129-2-3p				
Down	SIRPB2	1	hsa-mir-302a-3p				
Down	CLEC4C	1	hsa-mir-27a-3p				
Down	AGER	1	hsa-mir-191-5p				
Down	TIFAB	1	hsa-mir-146a-5p				

### Prediction of key TFs

The regulatory relationships between the target genes and their TFs were established using Cytoscape, which showed that the single gene was regulated by multiple TFs is shown in Fig.4b. Subsequently, 19 TFs (ex, FOXD1) targeting RGS4, 16 TFs (ex, GATA2) targeting EYA1, 12 TFs (ex, FOXL1) targeting GRIA4, 11 TFs (ex, TP53) targeting CCL19, 11 TFs (ex, JUND) targeting PRL, 15 TFs (ex, STAT3) targeting PRKACA, 14 TFs (ex, TFAP2A) targeting GAB2 , 12 TFs (ex, KLF5) targeting HIP1, 10 TFs (ex, PPARG) targeting PXN and 9 TFs (ex, HINFP) targeting RGL2 were verified in NetworkAnalyst database are listed in Table 6. Integrating with the result of REACTOME pathway analysis, it was indicated that these key target genes - TF network was mainly involved in the signaling by GPCR and innate immune system.

### Validation of hub genes

**Fig. 5:**
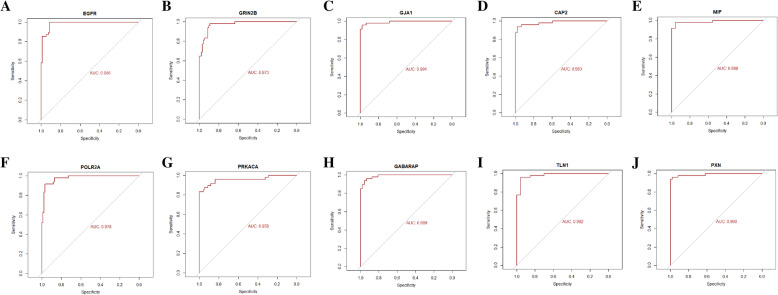
ROC curve analyses of hub genes. **a** EGFR. **b** GRIN2B. **c** GJA1. **d** CAP2. **e** MIF. **f** POLR2A. **g** PRKACA. **h** GABARAP. **i** TLN1. **j** PXN

**Fig. 6:**
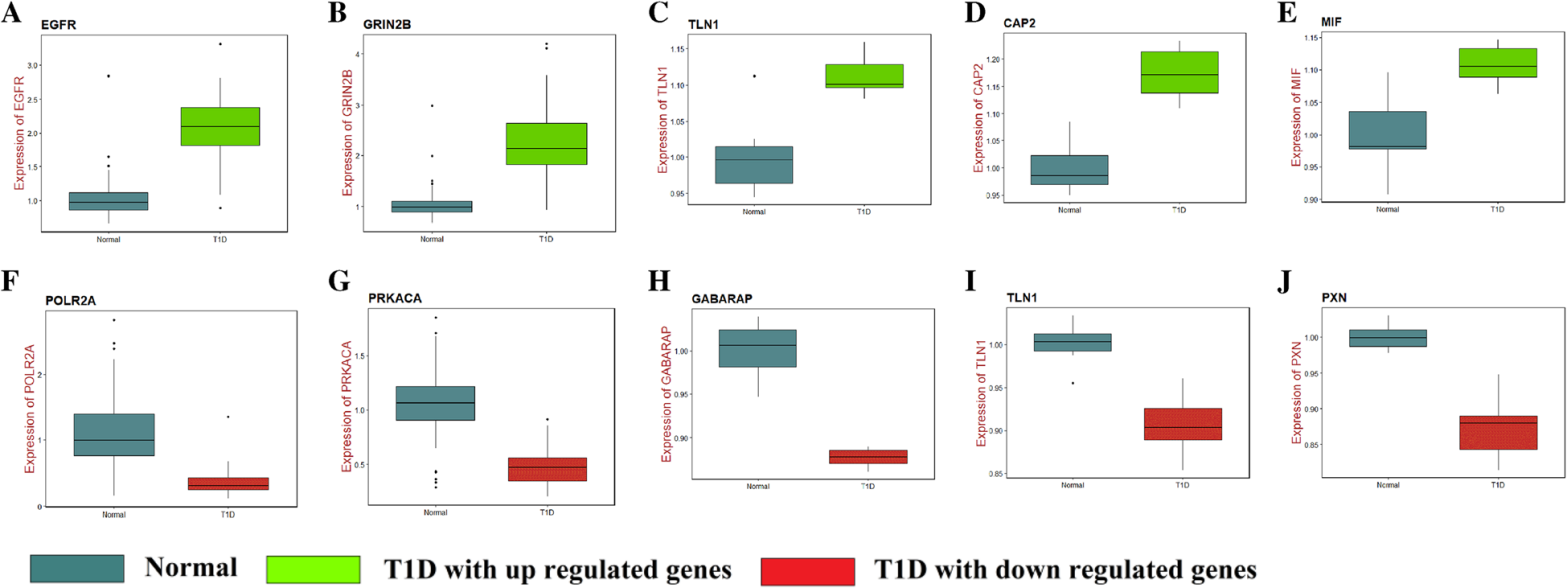
RT-PCR analyses of hub genes. **a** EGFR. **b** GRIN2B. **c** GJA1. **d** CAP2. **e** MIF. **f** POLR2A. **g** PRKACA. **h** GABARAP. **i** TLN1. **j** PXN

As these 4 genes are remarkably expressed in T1D, we executed a ROC curve analysis to calculate their sensitivity and specificity for the diagnosis of T1D. As shown in Fig. 5 EGFR, GRIN2B, GJA1, CAP2, MIF, POLR2A, PRKACA, GABARAP, TLN1 and PXN achieved an AUC value of >0.982, demonstrating that these genes have high sensitivity and specificity for T1D diagnosis. We further used RT-PCR to detect the mRNA expression of the hub gene. The 10 hub genes contain two up regulated genes (EGFR, GRIN2B, GJA1, CAP2 and MIF) and two down regulated gene (POLR2A, PRKACA, GABARAP, TLN1 and PXN). The RT-PCR data showed that although the trend of expression patterns of these 10 hub genes were consistent, among these up regulated genes, only EGFR, GRIN2B, GJA1, CAP2 and MIF were significantly up regulated in T1D. In addition, the expression of POLR2A, PRKACA, GABARAP, TLN1 and PXN were reduced in T1D (Fig. 6).

### Molecular Docking studies

The docking simulations are conducted in the present study is to identify the active site conformation and major interactions responsible for complex stability with the receptor binding sites. In T1D over expression of genes are identified and their proteins of x-ray crystallographic structure are selected from PDB for docking studies. The drugs containing thiazolidindione ring pioglitazone are most commonly used either alone or in combination with other antidibetic drugs. The docking studies of pioglitazone (as standard) and designed molecules containing the heterocyclic ring of thiazolidinedione have been carried out using Sybyl X 2.1 drug design software. The docking studies were performed to know the biding interaction of pioglitazone standard and designed molecules on identified over expressed genes of protein. The X- RAY crystallographic structure of two proteins from each over expressedgenes of epidermal growth factor receptor (EGFR), cyclase associated actin aytoskeleton aegulatory arotein 2 (CAP2), glutamate inotropic receptor NMDA type subunit 2B (GRIN2B), gap junction protein plpha 1 (GJA1) and macrophage migration inhibitory factor (MIF) and one protein from each of their co-crystallised PDB code 2XYJ, 4 K41, 5EWL, 2ZW3 and 4WR8 respectively were selected for the docking studies to identify and predict the potential molecule based on the binding score with the protein and effective against type 1 diabetes mellitus. A total of 54 molecules were designed and the binding score greater than six are believed to be good, few molecules obtained excellent binding score (C-score) with particular protein greater than 10The designed molecules obtained binding or score c- score less than 5 are TSPZP19, TBPZ38, TBPZ41 and TSIO4, TSPZ12, TBIO32, TBPZ40 TBPZ41 TBPZ42 TBPZ43 TBPZ44 TBPZ45 TBPZP46 and TSIO2, TSIO3, TSIO5, TSPZP20, TSPZP23, TBIO32, TBPZP48, TBPZP53 and TSIO1, TSIO9, TSPZ11, TSPZ12, TSPZ13, TSPZ14, TSPZ18, TSPZP19, TSPZP20, TSPZP23, TSPZP26, TSPZP27, TBIO28, TBIO30, TBIO31 TBIO32, TBIO35, TBIO36, TBPZ37, TBPZ39, TBPZ40, TBPZ41, TBPZ44, TBPZ45, TBPZP47, TBPZP49, TBPZP50 with PDB code of protein 2XYJ and 5EWL and 2ZW3 and 4WR8 respectively. The molecules obtained binding score 5 to 7 are TSIO1, TSIO2, TSIO3, TSIO4, TSIO5, TSIO7, TSIO8, TSIO9, TSPZ10, TSPZ11, TSPZ12, TSPZ13, TSPZ14, TSPZ16, TSPZ17, TSPZ18, TSPZP20, TSPZP21, TSPZP24, TSPZP25, TSPZP26, TBIO28, TBIO29, TBIO31, TBIO32, TBIO33, TBIO35, TBIO36, TBPZ39, TBPZ40, TBPZ42, TBPZ43, TBPZ40, TBPZ42, TBPZ43, TBPZ44, TBPZ45, TBPZP47, TBPZP48, TBPZP49, TBPZP50, TBPZP52, TBPZP53, TBPZP54 and TSIO1, TSIO2, TSIO3, TSIO5, TSIO6, TSIO7 TSIO8, TSIO9, TSPZ10, TSPZ11, TSPZ13, TSPZ14, TSPZ15, TSPZ16, TSPZ17, TSPZ18, TSPZP19, TSPZP20, TSPZP21 TSPZP22, TSPZP23, TSPZP24, TSPZP25, TSPZP26, TSPZP27, TBIO28, TBIO29, TBIO30, TBIO31, TBIO33, TBIO34, TBIO35, TBIO36, TBPZ37, TBPZ38, TBPZ39, TBPZ40, TBPZ42, TBPZ43, TBPZ44, TBPZ45, TBPZP46, TBPZP47, TBPZP49, TBPZP50, TBPZP51, TBPZP52, TBPZP53 and TSIO1, TSIO4, TSIO7, TSIO8, TSIO9, TSPZ10, TSPZ11, TSPZ12, TSPZ13, TSPZ14, TSPZ15, TSPZ16, TSPZ17, TSPZ18, TSPZP19, TSPZP21, TSPZP22, TSPZP24, TSPZP25, TSPZP26, TSPZP27, TBIO28, TBIO29, TBIO30, TBIO31, TBIO34, TBIO35, TBIO36, TBPZ37, TBPZ38, TBPZ39, TBPZ40, TBPZ41, TBPZ42, TBPZ43, TBPZ44, TBPZ45, TBPZP46 TBPZP47, TBPZP49, TBPZP50, TBPZP52 and TSIO2, TSIO3, TSIO4, TSIO5, TSIO6, TSIO7, TSIO8, TSPZ10, TSPZ15, TSPZ16, TSPZ17, TSPZP21, TSPZP22, TSPZP25, TBIO29, TBIO33, TBIO34, TBPZ38 TBPZ42, TBPZ43, TBPZP46, TBPZP48 with PDB protein 2XYJ and 5EWL and 2ZW3 and 4WR8 respectively. The molecules obtained c-score greater than 7 and less than 10 are TSIO6, TSPZ15, TSPZP22, TSPZP23, TSPZP27, TBIO30, TBIO34, TBPZ37, TBPZP46, TBPZP51, PIO (STD) and the molecules TSIO1, TSIO2, TSIO3, TSIO4, TSIO5, TSIO6, TSIO7, TSIO9, TSPZ10, TSPZ11, TSPZ12, TSPZ13, TSPZ14,TSPZ16, TSPZ17, TSPZ18, TSPZP19, TSPZP20, TSPZP21, TSPZP22, TSPZP23, TSPZP25, TSPZP26, TSPZP27, TBIO28, TBIO29, TBIO31, TBIO32, TBIO33, TBIO34, TBIO36, TBPZ37, TBPZ38, TBPZ39, TBPZ40, TBPZ41, TBPZ42, TBPZ43, TBPZ44, TBPZ45, TBPZP46 TBPZP47, TBPZP48, TBPZP49, TBPZP50, TBPZP51, TBPZP52, TBPZP53, TBPZP54, PIO (STD) and the molecules TSIO6, TBIO33, TBPZP51 and the molecule TBPZP51 with PDB code of 2XYJ and 4 K41 and 2ZW3 and 4WR8 respectively. Pioglitazone was used as a standard; it obtained good binding score of 8.396 and 7.648with two PDB proteins 2XYJ and 4WR8 respectively.

## Discussion

Pathological complications of T1D remains undetermined, hyperglycemia develops to play a key role [47]. Although T1D is rare compared to type 2 diabetics, it might affect in any part of the body, such as the eyes, kidneys, heart, peripheral and autonomic nervous systems [48]. Considering the poor prognosis of T1D, understanding the specific molecular biomarkers of the disease is important for initial diagnosis and therapy to increase survival rates. In this investigation, we examined the expression profiling by high throughput sequencing dataset GSE123658 including T1D group and healthy donors group to identify the molecular mechanism of T1D and seek some molecular biomarkers. Bioinformatics analysis of these biological factors is applied to explore genes that are favorable to treatment.

In total 284 DEGs, 142 up regulated and 142 down regulated genes were identified. Polymorphic gene ARMS2 has an important role in the advancement of T1D [49]. Polymorphic gene AS3MT was reported to be associated with progression of T1D [50]. Wang et al [51] also reported that CRHR2 are abnormally expressed in hypertension patients, but this gene might be induces hypertension in T1D patients. Investigation has indicated that RNF122 decrease expression is associated with hyperactivity disorder [52]; this finding is consistent with our results and indicates that RNF122 might be involved in the development of T1D. A previous investigation found that autophagy regulating TP53INP2 gene expression was associated with development of T1D [53]. Polymorphic gene CRTC2 is well known for its critical role in type 2 diabetes [54], but this polymorphic gene might be responsible for advancement of T1D.

GO and REACTOME pathway enrichment analyses were performed to explore interactions among the DEGs. These DEGs were mainly enriched in cell-cell signaling, integral component of plasma membrane, signaling receptor activity, signaling by GPCR, vesicle fusion, whole membrane, lipid binding and innate immune system. Altered MYH6 gene was known to play a role in congenital heart defects [55], but this gene might be induces T1D in patients with congenital heart defects. McKenna et al [56] found that AVP (arginine vasopressin) play essential roles in the T1D. Yue et al [57] concluded that high GRIA4 expression was correlated with abdominal aortic aneurysm progression, but this gene might be answerable for advancement of T1D in patients with abdominal aortic aneurysm. Kochetova el at [58] found that GRIN2B is a significant biomarker for type 2 diabetes compared to healthy controls, but it was confirmed for the first time in our study that GRIN2B expression in T1D indicates a good prognosis. Recent investigations demonstrated that DCC (DCC netrin 1 receptor) gene can mediate angiogenesis and plays an important role in diabetic kidney disease [59]. Ruiz de Azua et al [60] indicated that RGS4 was associated progression of type 2 diabetes, but this gene might be essential for T1D progression. A previous investigation demonstrated that GJA1 was associated with the progression of atrial fibrillation [61], but might be important gene signatures of T1D in patients with atrial fibrillation. Faienza et al [62] and Galán et al [63] demonstrated that, GREM1 and EGFR (epidermal growth factor receptor) might be important for predicting treatment response in T1D. TRHR (thyrotropin releasing hormone receptor) is considered a potential biomarker of hypertension [64], but this gene might be associated with development of T1D in patients with hypertension. PTPRT (protein tyrosine phosphatase receptor type T) has been reported to serve a role in obesity associated insulin resistance [65], but this gene might be involved in progression of T1D. Research has demonstrated that FCAMR (Fc fragment of IgA and IgM receptor) gene could contribute to progression of atherosclerosis [66], but this gene might be crucial for progression of T1D in patients with atherosclerosis. Sun et al. [67] revealed that CCL19 was associated with diabetic nephropathy in patients with T1D. DSP (desmoplakin) has been reported to be expressed in cardiomyopathy [68], but this gene might be linked with progression of T1D in patients with cardiomyopathy. Xu et al. [69] have demonstrated that ITIH4 are related with coronary heart disease, but this gene might be responsible for advancement of T1D in patients with coronary heart disease. Miyashita et al [70] demonstrate that GAB2 plays a role in Alzheimer disease progression, but this gene might be crucial for T1D in patients with Alzheimer disease. McCann et al. [71] showed that IGF2R played an important role in T1D. Previous studies reported that the expression of MYADM (myeloid associated differentiation marker) induced hypertension [72], but this gene might be liable for advancement of T1D in patients with hypertension. Fan et al. [73] and Chan et al. [74] revealed that TPCN2 and APOL1 were associated with type 2 diabetes, but these genes might be responsible for development of T1D. Previous investigation had confirmed that MEFV (MEFV innate immuity regulator, pyrin) play critical roles in ischemic heart disease [75], but this gene might be essential for progression of T1D in patients with ischemic heart disease. Wang et al [76] state that the expression of DTX4 is important event in obesity, but this gene might be linked with progression of T1D in patients with obesity.

We analyzed the protein–protein interactions (PPI) and modules of the DEGs involved in T1D. Table 5 summarizes the PPI network hub genes (five up regulated and five down regulated) that were identified in the T1D, which included EGFR, GRIN2B, GJA1, CAP2, MIF, POLR2A, PRKACA, GABARAP, TLN1 and PXN. A recent study showed that protein expression levels of CAP2 were up regulated in cardiomyopathy patients [77], but this gene might be responsible for T1D in patients with cardiomyopathy. MIF (macrophage migration inhibitory factor) [78] and KIF1A [79] were reported to be associated with T1D. Recent research suggested that PXN (paxillin) is involved in hypertension [80], but this gene might be associated with progression of T1D in patients with obesity. POLR2A, PRKACA, GABARAP, TLN1 and CIITA (class II major histocompatibility complex transactivator) might be considered as a novel biomarkers associated with the development of T1D.

Target gene - miRNA regulatory network and target gene - TF regulatory network analysis demonstrated that DEGs interacted directly or indirectly. The more edges associated with genes, miRNAs and TFs indicated the more potential selection for the targets. Table 6 summarizes the target gene - miRNA regulatory network and target gene - TF regulatory network (target genes, miRNAs and TFs) that were identified in the T1D, which included GRIN2B, EGFR, DKK1, GJA1, RGS4, TLN1, IGF2R, POLR2A, ARHGAP1, HIP1, RGS4, EYA1, CCL19, PRL, PRKACA, GAB2, HIP1, PXN , RGL2, hsa-mir-4257, hsa-mir-564, hsa-mir-587, hsa-mir-941, hsa-mir-561-3p, hsa-mir-4300, hsa-mir-5694, hsa-mir-378b, hsa-mir-3918, hsa-mir-6719-3p, FOXD1, GATA2, FOXL1, TP53, JUND, STAT3, TFAP2A, KLF5, PPARG and HINFP. STAT3 had been reported to be involved in the pathogenesis of T1D [81]. Polymorphic gene GATA2 has been reported to be crucial for the progression of coronary artery disease [82], but this gene might be responsible for advancement of T1D in patients with coronary artery disease. PRKACA (protein kinase cAMP-activated catalytic subunit alpha), DSP (desmoplakin), hsa-mir-4257, hsa-mir-564, hsa-mir-4300, hsa-mir-5694, RGS4, FOXD1, EYA1, TFAP2A and GAB2 might be considered as a novel biomarkers associated with the development of T1D.

In addition, we also performed validation of hub genes by ROC analysis and RT-PCR. Results showed that these hub genes differentiated T1D group from healthy donors group, and may be candidates for diagnostic biomarkers and new therapeutic target. Moreover, EGFR, GRIN2B, GJA1, CAP2, MIF, POLR2A, PRKACA, GABARAP, TLN1 and PXN are involved in progression of T1D.

**Fig. 7:**
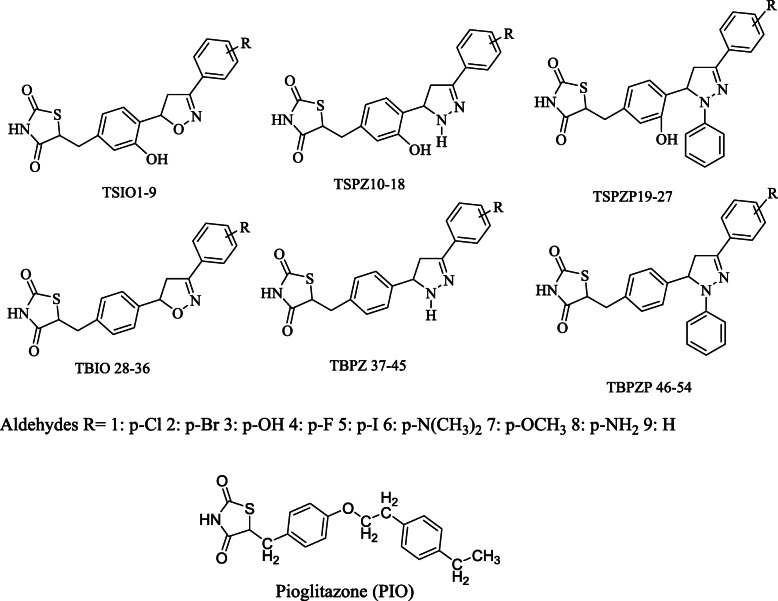
Structures of Designed Molecules

**Fig. 8:**
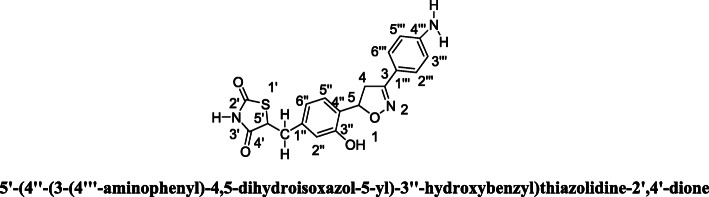
Structure of the Designed Molecule (TSIO8) obtained Highest Binding Score with PDB

**Fig. 9:**
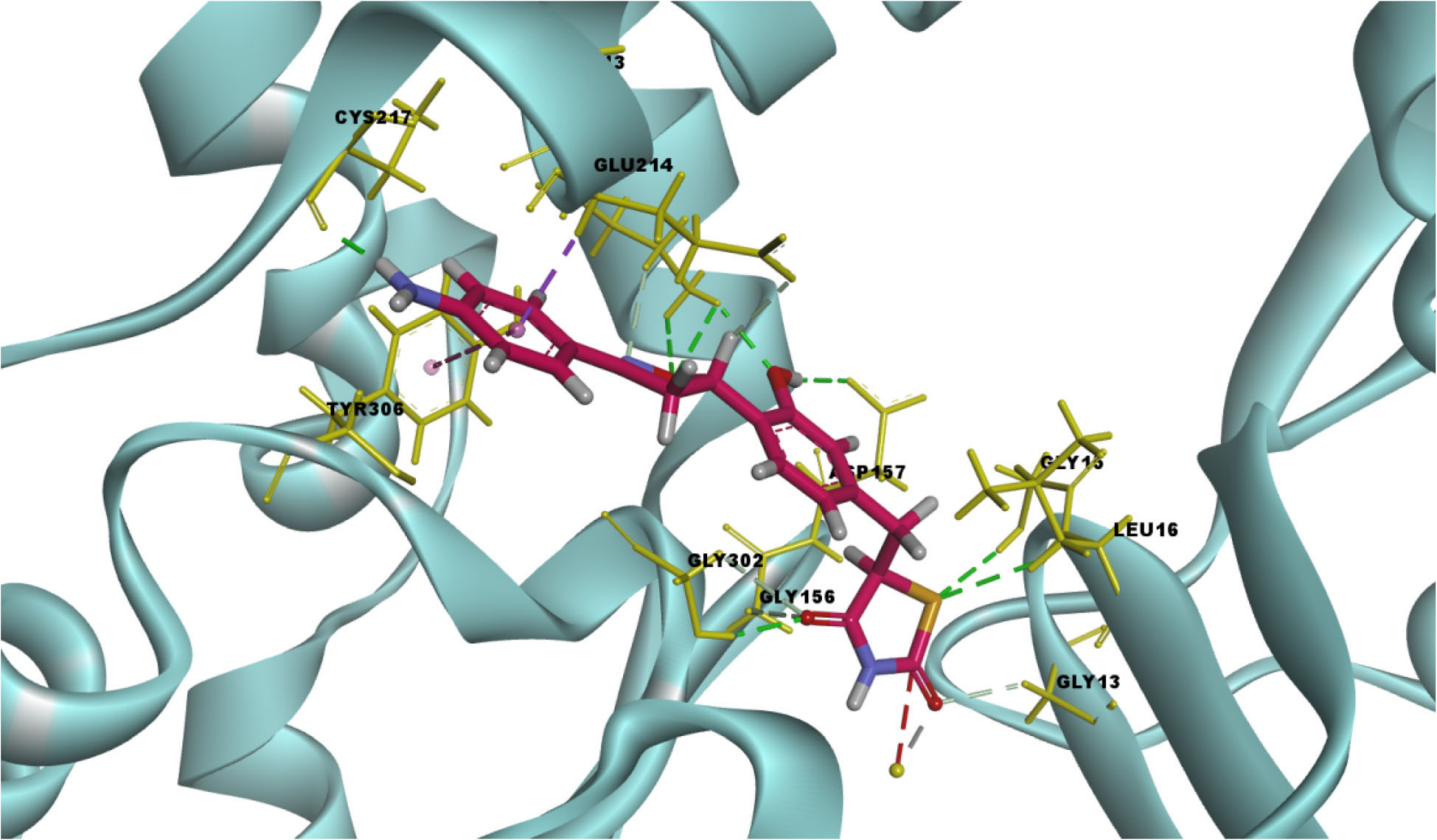
3D Binding of Molecule TSIO8 with 4 K41

**Fig. 10:**
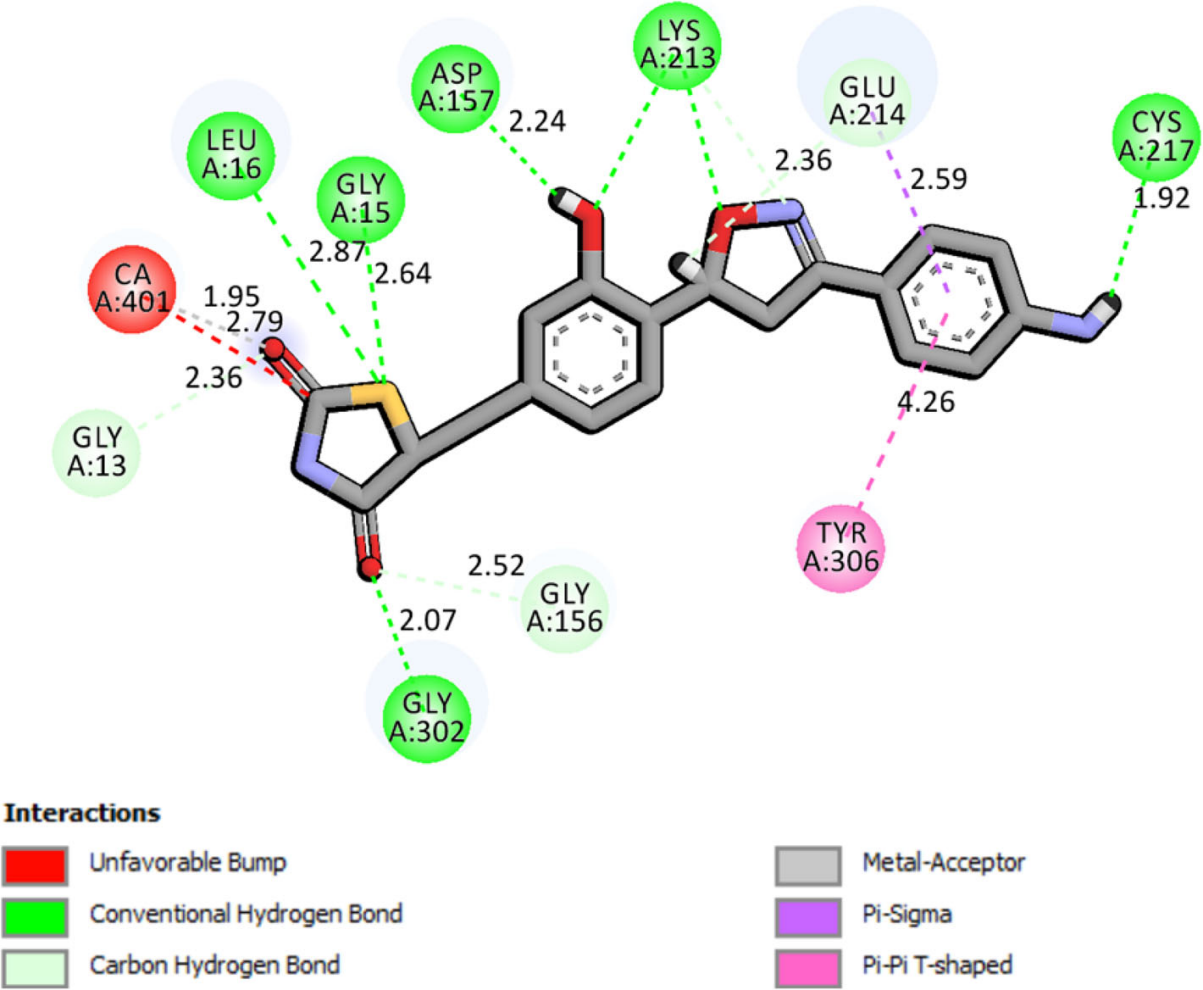
2D Binding of Molecule TSIO8 with 4 K41

Among all few molecules of TSIO8, TSPZ15, TSPZP24, TBIO30, TBIO35 (Fig.7) obtained excellent binding score of 11.084 with PDB code 4 K41, the values are depicted in Table 7. The molecule TSIO8 (Fig. 8) has highest binding score its interaction with protein 4 K41, and formed hydrogen bond interaction of 4″’ –NH_2_ group with CYS-217, the hetero atom 1 oxygen and 3″ aromatic hydroxyl group (−OH) formed hydrogen bonding interactions with same amino acid LYS-213. The 3″ hydroxyl group also farmed hydrogen bond interaction with ASP-157 respectively. The sulphur of thiazolidinone ring 1′-S formed two hydrogen bond interactions with different amino acids LEU-16 & GLY-15 and 4′-C ring carbonyl formed interaction with GLY-302 respectively. The molecule also formed pi-pi interactions of electrons of aromatic ring with TYR-306, and pi-sigma interaction with GLU-214 and 2′ C=O of thiazolidine ring formed unfavourable interaction with calcium 401 (Ca) following 2′ C=O formed carbon-hydrogen interaction with GLY-13, All interactions with amino acids and metal are depicted by 3D (Fig.9) and 2D (Fig.10) figures.

**Table 7:** Docking results of Designed Molecules on Over Expressed Proteins

Sl. No/Code	Over expressed gene: EGFR			Over expressed gene: CAP2			Over expressed gene: GRIN2B			Over expressed gene: GJA1			Over expressed gene: MIF		
	PDB: 2XYJ			PDB:4 K41			PDB:5EWL			PDB: 2ZW3			PDB: 4WR8		
	Total Score	Crash (−Ve)	Polar	Total Score	Crash (−Ve)	Polar	Total Score	Crash (−Ve)	Polar	Total Score	Crash (−Ve)	Polar	Total Score	Crash (−Ve)	Polar
TSIO1	5.708	−1.859	2.223	9.497	−0.989	5.432	5.265	−1.280	4.250	5.286	−1.003	0.800	4.725	−1.368	1.708
TSIO2	5.105	−1.784	2.893	9.728	−1.388	5.353	5.521	−1.228	3.688	4.881	−0.951	2.131	5.419	−2.125	0.841
TSIO3	5.729	−1.113	1.112	9.456	−2.449	4.970	5.195	−2.525	3.514	4.584	−1.042	0.965	5.331	−4.193	3.443
TSIO4	5.842	−0.864	0.598	10.152	−1.938	6.319	4.315	−1.041	3.793	5.266	−2.776	1.233	5.417	−1.767	1.069
TSIO5	5.500	−1.562	1.525	9.432	−1.132	5.363	5.577	−1.238	3.432	4.921	−0.988	2.079	5.590	−1.579	3.093
TSIO6	7.204	−1.628	3.166	10.018	−0.815	5.325	6.381	−2.325	2.695	7.272	−1.458	0	5.970	−1.865	1.184
TSIO7	5.602	−2.721	1.729	9.631	−1.052	5.231	5.244	−2.231	2.943	5.489	−1.284	1.538	5.760	−3.956	3.505
TSIO8	6.934	−1.501	2.102	11.084	−2.034	7.653	5.869	−1.008	3.605	5.258	−3.205	1.973	5.406	−2.204	0.608
TSIO9	6.729	−0.869	1.107	9.449	−1.024	5.437	6.182	−1.013	3.745	6.191	−2.129	1.364	4.990	−1.732	1.135
TSPZ10	5.806	−1.608	2.698	9.531	−1.000	5.404	5.049	−1.424	4.118	5.824	−0.884	2.163	5.158	−0.943	1.423
TSPZ11	5.062	−1.899	1.706	9.691	−1.139	6.313	5.810	−1.269	4.181	6.165	−1.190	0.465	4.706	−1.751	1.18
TSPZ12	6.254	−1.409	2.367	9.527	−2.615	5.096	4.213	−1.174	3.821	5.348	−1.272	2.181	4.378	−2.139	0.396
TSPZ13	6.562	−1.307	5.202	9.499	−1.016	5.274	5.150	−0.841	3.722	5.735	−0.931	2.155	4.606	−2.490	1.565
TSPZ14	5.129	−1.943	1.634	9.654	−0.964	5.326	5.671	−1.154	4.189	5.812	−1.097	0.269	4.928	−2.712	1.038
TSPZ15	7.059	−2.752	2.284	10.263	−1.023	5.207	5.431	−1.739	2.642	6.811	−1.633	1.323	6.368	−2.305	0.113
TSPZ16	6.160	−2.286	0.006	8.689	−2.540	4.568	5.913	−0.710	3.105	6.013	−1.135	2.082	6.021	−2.462	0.222
TSPZ17	6.407	−1.750	0.937	9.812	−2.408	5.061	5.800	−1.002	3.600	6.514	−1.207	0.506	5.130	−2.065	0.440
TSPZ18	6.568	−1.612	1.112	8.767	−2.126	5.501	5.188	−1.351	4.072	5.871	−1.577	2.158	4.798	−1.301	1.228
TSPZP19	4.784	−2.116	0.003	8.602	−1.654	6.396	5.398	−1.745	3.543	5.147	−1.504	1.124	4.275	−2.790	0
TSPZP20	5.781	−2.549	1.063	8.219	−3.381	3.885	5.780	−1.815	3.600	4.684	−1.714	2.044	4.805	−1.447	0.001
TSPZP21	5.031	−5.142	1.128	9.111	−3.271	5.677	5.659	−1.312	2.571	5.204	−1.613	0.919	5.660	−1.490	0.020
TSPZP22	7.624	−1.675	1.109	8.814	−2.974	4.716	5.367	−1.149	3.704	5.641	−0.878	1.196	5.631	−1.579	1.198
TSPZP23	7.198	−1.682	0.115	8.854	−1.658	6.596	5.582	−1.513	2.814	4.523	−0.907	0.033	4.134	−3.073	1.121
TSPZP24	6.026	−2.349	0.116	10.430	10.430	4.262	6.320	−1.665	2.335	5.267	−1.261	1.159	7.992	−1.273	1.256
TSPZP25	5.006	−5.129	1.127	8.972	−3.033	3.770	5.772	−1.268	3.687	5.658	−1.419	1.289	5.515	−1.345	0
TSPZP26	5.267	−5.637	0.954	8.539	−1.481	6.14	6.208	−1.424	2.718	5.394	−2.135	0.914	4.436	−1.363	0
TSPZP27	7.221	−2.321	0.498	8.648	−1.512	6.329	5.863	−1.335	2.652	5.038	−1.501	0.936	4.689	−3.173	1.167
TBIO28	5.387	−2.438	2.608	8.796	−1.210	4.261	5.251	−0.834	1.829	5.187	−0.854	0.744	4.877	−1.324	2.044
TBIO29	5.132	−1.505	1.865	9.195	−1.258	4.255	5.170	−0.793	1.803	5.125	−1.417	1.155	5.035	−1.222	1.403
TBIO30	7.079	−1.923	0.858	10.531	−1.372	4.924	5.586	−1.159	3.148	5.276	−1.395	0.930	4.453	−0.929	0
TBIO31	6.090	−1.967	1.929	8.801	−0.773	4.174	5.224	−0.770	1.840	5.232	−1.215	1.152	4.573	−1.631	1.900
TBIO32	5.157	−2.015	0.004	8.867	−0.924	4.276	4.924	−1.049	2.634	4.916	−1.432	1.125	4.759	−1.525	1.991
TBIO33	5.796	−2.689	0.709	9.892	−0.980	4.358	5.024	−1.110	1.764	7.055	−2.062	0.020	5.620	−1.766	1.242
TBIO34	7.079	−1.923	0.858	9.161	−0.875	4.317	5.143	−0.657	2.822	5.051	−0.845	2.321	5.584	−1.952	1.290
TBIO35	6.542	−2.270	1.959	10.372	−1.124	5.275	5.860	−1.513	2.938	5.191	−1.312	0.875	4.650	−0.957	0
TBIO36	6.164	−1.506	0.996	8.912	−1.847	4.162	5.036	−0.853	1.831	5.431	−1.234	0.002	4.195	−1.850	1.971
TBPZ37	7.202	−1.109	0.108	9.602	−3.041	5.880	5.126	−0.951	1.779	5.859	−1.115	0.010	4.667	−1.195	2.460
TBPZ38	4.596	−1.574	1.121	8.762	−0.815	4.21	5.222	−0.698	1.828	5.400	−0.971	0.001	5.375	−2.176	1.014
TBPZ39	6.070	−1.439	2.219	9.392	−2.213	5.155	5.769	−0.800	3.918	5.772	−0.874	0	4.639	−1.87	1.522
TBPZ40	6.090	−1.967	1.929	8.590	−2.055	4.1	5.214	−0.842	1.778	5.514	−2.298	1.184	4.822	−1.851	1.535
TBPZ41	4.679	−1.469	0.004	8.557	−1.073	4.238	4.997	−0.743	1.812	5.448	−1.147	0.006	4.676	−1.163	1.122
TBPZ42	6.848	−1.124	2.084	9.516	−1.040	4.385	5.476	−1.344	2.358	6.764	1.783	1.317	6.946	−1.384	1.490
TBPZ43	5.582	−2.315	1.677	8.831	−0.898	4.325	5.462	−1.872	3.436	5.171	−0.661	3.177	5.013	−2.468	1.121
TBPZ44	6.645	−2.275	1.936	9.094	−1.764	3.939	5.892	−1.063	2.964	5.503	−1.122	0.004	4.693	−1.324	0
TBPZ45	6.378	−1.607	1.110	8.557	−0.955	4.336	5.230	−0.769	1.783	6.371	−1.899	1.195	4.903	−1.763	1.523
TBPZP46	7.202	−1.109	0.108	6.165	−1.473	1.634	5.585	−0.786	3.893	6.324	−1.314	3.361	5.355	−2.860	2.421
TBPZP47	5.315	−2.731	0.963	8.903	−1.907	5.544	5.410	−0.992	1.830	5.449	−0.886	1.176	3.173	−0.976	0
TBPZP48	5.006	−5.129	1.127	9.231	−3.021	5.149	4.034	−1.612	3.442	4.721	−1.372	0.002	6.037	−1.283	0.845
TBPZP49	6.876	−3.317	0.951	8.730	−2.958	4.111	5.451	−1.136	1.818	5.708	−1.038	0.021	4.074	−3.978	0.251
TBPZP50	6.309	−2.914	0.007	8.787	−2.397	3.629	5.312	−0.998	1.802	5.374	−1.282	0.002	4.069	−2.916	0
TBPZP51	7.331	−1.709	1.128	9.755	−2.543	3.496	5.324	−1.063	1.831	7.841	−1.751	1.304	8.13	−1.286	1.322
TBPZP52	5.470	−3.384	0.063	8.598	−3.204	3.198	5.494	−1.324	3.039	5.632	−1.046	4.503	5.727	−1.639	0.743
TBPZP53	6.402	−4.890	1.693	8.645	−1.982	4.767	5.373	−0.959	1.826	4.790	−2.041	1.079	5.994	−1.661	1.073
TBPZP54	6.925	−2.649	6.032	5.452	−2.612	3.535	4.521	−1.365	3.442	5.732	−1.353	1.328	5.325	−1.551	0.113
PIO (STD)	8.396	−0.983	1.023	6.683	−1.814	2.537	6.261	−0.828	2.212	6.232	−2.171	0	7.648	−1.772	0.325

In conclusion, the present investigation was designed to identify DEGs that may be involved in the progression of T1D. A total of 284 DEGs and 10 hub genes were identified and might be regarded as diagnostic biomarkers and new therapeutic target for T1D. Together, EGFR, GRIN2B, GJA1, CAP2, MIF, POLR2A, PRKACA, GABARAP, TLN1 and PXN might be effective targets in T1D, but more experimental investigations and clinical trials are needed.

## Data Availability

The datasets supporting the conclusions of this article are available in the GEO (Gene Expression Omnibus) (https://www.ncbi.nlm.nih.gov/geo/) repository. [(GSE123658) (https://www.ncbi.nlm.nih.gov/geo/query/acc.cgi?acc=GSE123658)]
